# Supplementary material for: Ultrafast carrier dynamics in terahertz photoconductors and photomixers: beyond short-carrier-lifetime semiconductors
Source: Nanophotonics. 2022 Mar 10;11(11):2661–91. doi: 10.1515/nanoph-2021-0785 (PMC11501130; doi:10.1515/nanoph-2021-0785)
Supplement: Supplementary file 1 — Supplementary Material [file j_nanoph-2021-0785_suppl_001.docx]

**Supplementary Material:**

**Ultrafast Carrier Dynamics in Terahertz Photoconductors and Photomixers: beyond Short-Carrier-Lifetime Semiconductors**

Ping-Keng Lu^1^, Anuar de Jesus Fernandez Olvera^2^, Deniz Turan^1^, Tom Sebastian Seifert^3^, Nezih Tolga Yardimci^1^, Tobias Kampfrath^3^, Sascha Preu^2^, Mona Jarrahi^1,*^

^1^ Electrical and Computer Engineering Department, University of California, Los Angeles, CA, USA.

^2^ Department of Electrical Engineering and Information Technology, Technical University Darmstadt, Darmstadt, Germany.

^3^ Department of Physics, Freie Universität Berlin, 14195 Berlin, Germany.

^4^ Department of Physical Chemistry, Fritz Haber Institute of the Max Planck Society, 14195 Berlin, Germany.

**Detailed calculations for the current generated by photodiodes and photoconductors**

The methodology applied here is basically the generalized version of the one in ref. [1] where the contributions of all partial currents generated in the device are integrated up in order to yield the total current generated within the photomixer/photoconductor.


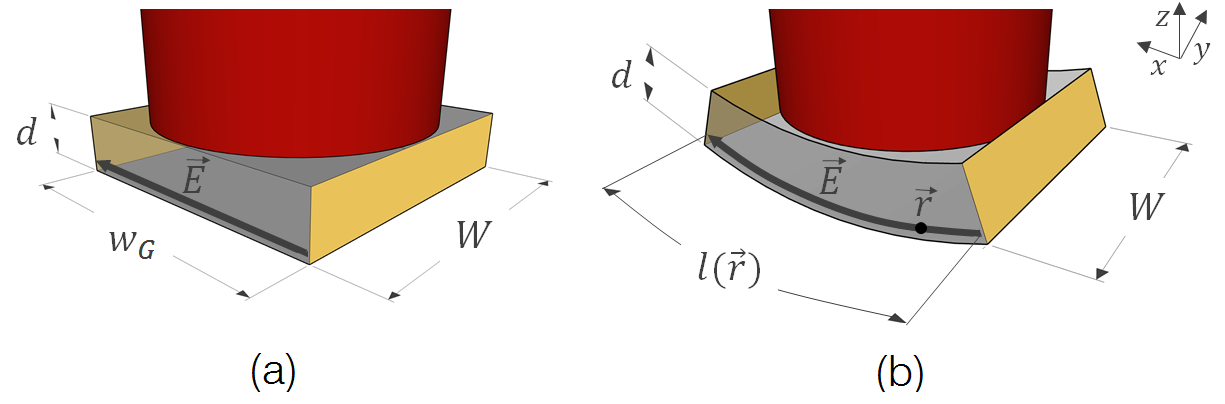


*Fig.1: Integration volume, electrode geometry and field lines. a) case of plane-parallel electrodes (e.g. pin diodes with layered contact materials) and b) a more complicated, generalized version of the electrode structure.*

Let us first discuss the case of plane-parallel contacts as illustrated in Fig. 1 a) and derive the current contribution of a single electron-hole pair. The field lines between the electrodes are all parallel and perpendicular on the electrodes, allowing to reduce the problem to a 1D derivation. We start with a current resulting from an optically generated charge density $n_{e}=n_{h}=n$ in an infinitesimally small volume at a distance $x$ away from the positive electrode. For now, we assume that no charge is trapped, i.e. all charges will eventually be removed from the generation volume by the current. We begin with electrons. For the electrons travelling with a velocity $v_{e}\left( \tau\right)$, the time required for reaching the electrode $\tau_{tr}^{e}\left( x \right)$is defined over the integral

$x=\int_{0}^{\tau_{tr}^{e}(x)} v_{e}\left( \tau\right)d\tau$ (S1)

Expanding Eq (S1) on either side by the charge density yields

$en_{e}x={\int_{0}^{\tau_{tr}^{e}\left( x \right)} en_{e}v_{e}\left( \tau\right)d\tau}$ (S2)

where the integrand on the right-hand side is indeed the current density $j\left( \tau\right)=en_{e}v_{e}\left( \tau\right)$. Integrating the current density over the cross section $A$yields the total current generated by the electrons,

$en_{e}xA={{\int_{0}^{\tau_{tr}^{e}\left( x \right)} I_{e}\left( \tau\right)d\tau}=Q_{e}.}$ (S3)

For the last equality, we have used that the time integral of the current is the total charge provided by the current. We can repeat the formalism for holes that are transported to the opposite electrode, covering a distance of $l-x,$ where $l=w_{G}$ is the distance of the electrodes

$Q_{h}=en_{h}\left( l-x \right)A,$ (S4)

where $n_{e}=n_{h}=n$ as photons always generate electron-hole pairs. The total charge delivered by electrons and holes sums up to $Q_{e}+Q_{h}=enAl=Q$, i.e. the total charge within the volume by the charges generated at position $x$. In other words, one photon generates one net charge that is distributed to a partial electron and a partial hole charge. This is indeed a consequence of particle number conservation. Normalizing Eq. (S3) to the total charge yields the partial charge delivered by one electron,

$q_{e}(x)=\int_{0}^{\tau_{tr}^{e}\left( x \right)} \frac{ev_{e}\left( \tau\right)}{l}d\tau$ (S5)

The integrand $i_{e}\left( \tau\right)=\frac{ev_{e}\left( \tau\right)}{l}$ is the unit current generated by a single electron. The more general case in Fig. 1 b) is only slightly more complicated. The field lines are not constant and not parallel over the whole volume, i.e. the field has to be generally treated as a vectorial quantity instead of a scalar. However, electrons are accelerated by the DC electric field. They will follow the field lines irrespective of how complicated the electrode structure and field distribution might be. An important feature of a field line is that it is characterized by a constant field strength along the field line. Thus, the charge transport can be described by the scalar velocity $v\left( \vec{r},\tau\right)=\pm$|$\vec{v}\left( \vec{r},\tau\right)|$ which can be simply derived from the (scalar!) field strength on the field line, at least approximately. The scalar representation does not consider energy loss due to a change in direction of motion. For photoconductors and pin diodes, however, the direction of the electric field usually changes only slowly and smoothly, therefore, the energy loss by the directional change (in other words, the vectorial character of the terms in Eq. (7) of the main manuscript that may not be parallel to each other) is very small. The scalar description strongly simplifies the derivation as Eq. (6) and (7) are decoupled and can be treated and calculated independently. Eq. (6) describes the current on a macroscopic scale considering the interference of all currents running through the calculation volume, while Eq. (7) describes the microscopic transport kinetics based on semiconductor physics.

The length $l$ in Eq. (S5) will be the length of the respective field line, reducing the problem to scalar quantities only. One complication still remains. For the simple case in Fig. 1 a), all field lines have same length, namely $l=w_{G},$ however, for the case of Fig. 1 b) the length of the field line the charges travel on depends on their generation point, turning the solution more elaborated.

With the elementary current at hand, we can now calculate the currents generated throughout the volume. Next, we calculate the number of charges generated by the optical power. An incident laser signal generates electron-hole pairs via absorption within the photoconductor. The rate at which carriers are generated is given by Eq. (1) of the main text. They are distributed over the absorber (i.e. photoconductor/photomixer) volume according to their generation point $\vec{r}$and their generation time $t^{'}.$The spatial dependence of the laser intensity, $I_{L}\left( \vec{r},t^{'} \right)$ is due to the distribution of the laser power, e.g. by a Gaussian spot or modified by plasmonic effects. The time dependence simply originates from the fact that the optical signal is either a pulse or a heterodyned laser signal. The local absorption coefficient may also add spatial dependence, e.g. if the absorber is composed of different materials, e.g. InGaAs and InP in the case of UTC or p-i-n diodes or by plasmonic enhancement if the absorption takes place close to a metal electrode. Both of these dependencies are expressed by the generation rate $g\left( \vec{r},t^{'} \right)$, as given in the main text. The total current generated by one charge type, e.g. electrons is then

$I_{e}\left( t \right)=\int_{V} \int_{\tau=0}^{\tau=\tau_{tr}^{e}} g\left( \vec{r},t^{'} \right)i_{e}\left( \vec{r},\tau\right)d\tau dV$, with $\tau=t-t^{'}$. (S6)

The latter being the time elapsed between the generation time point of the charge cloud and the actual time. Rewriting Eq. (S6) by substituting the elementary current allows to re-express it in terms of a current density

$I_{e}\left( t \right)=\int_{V} \frac{1}{l}\int_{t-t^{'}=0}^{t-t^{'}=\tau_{tr}^{e}} g\left( \vec{r},t^{'} \right){ev}_{e}\left( \vec{r},t-t^{'} \right)dt'dV$. (S7)

The integrand can be considered as a current density${j_{e}\left( \vec{r},t \right)=\int_{t-t^{'}=0}^{t-t^{'}=\tau_{tr}^{e}} g\left( \vec{r},t^{'} \right)ev}_{e}\left( t-t^{'} \right)dt'$. We remark that a scalar description of the electron velocity as in Eq. (S7) is only correct for the simple case illustrated in Fig. 1 a). For the more general case, the velocity is again a vector. Again, we can use the fact that the electrons will follow the field line. This means, we have to replace the spatial integration by

$v_{e}\left( \vec{r},t-t^{'} \right)dV\to\vec{v}(\vec{r},t-t')\cdot\vec{n}_{E}(\vec{r})dV$. (S8)

In the case of a low lifetime photoconductor, carriers are trapped before reaching their respective electrode. The general trapping process is given by the differential equation

$\frac{\partial n\left( \vec{r},t \right)}{\partial t}=-\frac{n\left( \vec{r},t \right)}{\tau_{rec}}$, (S9)

with the solution of an exponential decay with a time constant $\tau_{rec}$, as stated in the main text. For the velocity of a charge, we take its ensemble average which is deterministic. Therefore, it solely depends on the time difference between the generation time and the actual time, $\tau=t-t'$. It further possesses a spatial dependence as the accelerating electric field may have a spatial dependence, e.g. for the case of inhomogeneous DC field distribution in a photoconductor with planar electrodes as illustrated in Fig. 1 b), or for the case of low fields in the non-depleted absorber of a MUTC diode as compared to the comparatively high fields in a depleted absorber. In summary, the partial currents can be expressed as a current density, as given by Eq. (3) of the main text, where the integration boundaries have been replaced by Heavyside step functions and extended the integration boundaries to infinity. Now the integral only needs to be performed over all generation times, or equivalently, over all time differences $t-t^{'}$. Formally, the integral can be seen as a convolution of the generation rate and the Green’s function of Eq. (5) given the main text, which takes the complete transport kinetics into account. In a final step, we have to integrate the current density over all generation points, as already included in Eq. (S7), where *V* is the photoconductor or photomixer volume. For the case shown in Fig. 1 a), the length $l$ is constant throughout the integration volume, i.e. the distance of the electrodes. It can therefore be taken in front of the integral. In the more general case shown in Fig. 1 b), however, the length $l=l\left( \vec{r} \right)$ depends on the generation point of the charge. The generated current is thus

$I\left( t \right)=\int_{V} \frac{1}{l(\vec{r})}\vec{j}\left( \vec{r},t \right)\cdot\vec{n}_{E}(\vec{r})dV$. (S10)

We remark that Eq. (S10) has to be solved for electrons (${I\left( t \right)=I}_{e}\left( t \right)$) and holes (${I\left( t \right)=I}_{h}\left( t \right)$) separately, yielding the total photocurrent ${I_{tot}\left( t \right)=I}_{e}\left( t \right)+I_{h}(t)$.

For geometries that are not as simple as the one shown in in Fig. 1 a), the average transport length, weighted by the current that travels on itmay be a more intuitive quantity given by

$l_{eff}=\frac{\int_{V} \vec{j}\left( \vec{r},t \right)\cdot\vec{n}_{E}(\vec{r})dV}{\int_{V} \frac{1}{l(\vec{r})}\vec{j}\left( \vec{r},t \right)\cdot\vec{n}_{E}(\vec{r})dV}$. (S11)

In case of plasmonic mixers, $l_{eff}$ can become very short, of the order of a few 100s nm, as most electrons are created close to their respective electrode. The long transport length of holes to the other electrode will then strongly decrease their contribution to the total current such that they can actually be neglected. Electrons, instead, are removed very efficiently, even in a short lifetime material, with a transit-time that can even be shorter than the recombination time.

As an intermediate step, we will calculate the photoconductive gain, being the ratio of the actual generated photocurrent and the ideal photocurrent. The ideal photocurrent is given by the absorbed optical power by simply integrating Eq. (1) multiplied with the elementary charge over the whole volume,

$I_{id}\left( t \right)=\int_{V} \alpha\left( \vec{r},\nu\right)\cdot\frac{eI_{L}\left( \vec{r},t^{'} \right)}{h\nu}dV$ (S12)

The simplest way for calculating the photoconductive gain for a complex structure with spatially dependent transit times is by investigating the DC components only, which can easily be accessed by averaging over one period (CW photomixing) or the time between two subsequent pulses (TDS case)

$g=\frac{{<I}_{tot}\left( t \right)>}{<I_{id}\left( t \right)>}$ (S13)

For CW heterodyning of two lasers, it suffices to just take the DC part of the optical signal.

We now turn to the example cases discussed in the main manuscript, where we assumed plane-parallel electrode structures for simplicity. In such case, the length of all field lines is identical to the spacing of the electrodes $l\left( \vec{r} \right)=w_{G}=const$ as illustrated in Fig. 1a). We also assumed a constant carrier velocity to simplify the temporal integral.

1.) Low lifetime photoconductor, CW excitation: $\tau_{rec}\ll\tau_{tr}(r)=\frac{r}{v}$ for almost all cases except a very small region very close to the target electrode of the charge. We consider that the laser power $P_{L,0}$ decays exponentially upon penetration into an absorbing material as $P_{L,0}\left( z \right)=P_{L,0}exp(-\alpha z)$, according to Lambert-Beer law. Hence $g\left( \vec{r},t^{'} \right)= \alpha\cdot\frac{P_{L,0}(z)\cdot(1+\cos\omega t^{'})}{A\cdot h\nu}.$

Eq. (S10) then turns into

$I\left( t \right)=\frac{1}{w_{G}}\int_{V} \int_{t-t^{'}=0}^{t-t^{'}=\tau_{tr}} e\alpha\cdot\frac{P_{L,0}(z)\cdot(1+\cos\omega t^{'})}{A\cdot h\nu}v\exp\left[ -\frac{t-t^{'}}{\tau_{rec}} \right]dt'dV.$ (S14)

The volume integral decomposes as $dV=dAdz$.The integrals in Eq. (S14) can then be decomposed in two independent integrals, namely

$I\left( t \right)=\int_{0}^{d} e\alpha\frac{P_{L,0}\left( z \right)}{h\nu}dz\cdot\int_{t-t^{'}=0}^{t-t^{'}=\tau_{tr}} \frac{v}{w_{G}}\cdot(1+\cos\omega t^{'})\exp\left[ -\frac{t-t^{'}}{\tau_{rec}} \right]dt'$ , (S15)

where the first term is the ideal photocurrent, given by the amount of absorbed laser power, and the second term considers the decrease of the current due to carrier recombination plus interference effects for the AC component. The first integral yields

$I_{id}=\frac{eP_{L,0}}{h\nu}(1-\exp\left[ -\alpha d \right])$ (S16)

where we have assumed a thickness of the photoconductive layer *d*. For the time integral, represented by $\eta\left( \omega\right)$, the assumption $\tau_{tr}\gg\tau_{rec}$allows to set the upper integration boundary to infinity, yielding

$\eta\left( \omega\right)=\frac{v}{w_{G}}\left( \tau_{rec}+\frac{\tau_{rec}}{\sqrt{1+\left( \omega\tau_{rec} \right)^{2}}}\cdot\cos(\omega t+\varphi) \right)$, (S17)

with $\varphi=\arctan\left( \omega\tau_{rec} \right)$. The prefactor $\frac{v}{w_{G}}=\left( \tau_{tr}^{max} \right)^{-1}$ is the inverse of the transit time. With the photoconductive gain $g=\frac{\tau_{rec}}{\tau_{tr}^{max}}$ we can now rewrite the solution to Eq. (S14) for this case as

$$I\left( t \right)=g\frac{eP_{L,0}}{h\nu}\left( 1-\exp\left[ -\alpha d \right] \right)\cdot\left( 1+\frac{1}{\sqrt{1+\left( \omega\tau_{rec} \right)^{2}}}\cdot\cos\left( \omega t+\varphi\right) \right)=$$

$=gI_{id}\cdot\left( 1+\frac{1}{\sqrt{1+\left( \omega\tau_{rec} \right)^{2}}}\cdot\cos\left( \omega t+\varphi\right) \right)$ (S18)

The key feature is that both AC and DC term are reduced by the photoconductive gain, $g$, strongly reducing the thermal load of the device for short recombination times, while the onset of the lifetime roll-off is shifted towards higher frequencies with a 3 dB frequency of $f_{LT}^{3dB}=\frac{1}{2\pi\tau_{rec}}$. For this reason, most photoconductors employ sub-ps carrier lifetimes, though it is not generally mandatory and as we have shown in the main manuscript. There are also other solutions for achieving a decent performance at terahertz frequencies by minimizing the transit time.

2.) Long lifetime material or p-i-n diode, continuous-wave excitation, $\tau_{rec}\gg\tau_{tr}$. The ideal photocurrent can be calculated the same way as for case 1, except that the depth $d$ can take different roles: for vertical illumination of a photoconductor or a p-i-n diode, $d$ is the thickness of the absorbing layer. For a waveguide-integrated photodiode, $d$ is the propagation direction of the wave along the waveguide, consequently, a reduced absorption coefficient has to be used, corresponding to the effective absorption coefficient experienced by the optical field that touches the absorber layer. Also $w_{G}$ takes different roles, depending on the type of device: for a photoconductor, $w_{G}$ is the electrode distance along to its surface. For p-i-n diodes the electrodes are usually vertical, i.e. along the z-direction. Light propagation direction and charge transport direction are thus parallel. Again, we assume a constant electron or hole charge velocity, $v_{e,h}$. First we discuss the case where the light propagation and the transport direction are orthogonal, e.g. the case of a photoconductor under vertical illumination with horizontal electrodes or the case of a waveguide-integrated UTC- or photodiode where the transport is vertical and the absorption takes place parallel to the surface. For a very thin absorber region, where all carriers roughly feature the same transit time $\tau_{tr}$Eq. (S10) becomes

$I\left( t \right)=\frac{eP_{L,0}}{h\nu}\left( 1-\exp\left[ -\alpha d \right] \right)\cdot\int_{t-t^{'}=0}^{t-t^{'}=\tau_{tr}} \frac{v_{e,h}}{w_{G}}\cdot(1+\cos\omega t^{'})dt^{'}$, (S19)

with the solution

$I_{UTC}\left( t \right)=I_{id}\left( 1+\mathrm{sinc}\left( \frac{\omega\tau_{tr}}{2} \right)\cos\left( \omega t+\frac{\omega\tau_{tr}}{2} \right) \right)$, (S20)

i.e. Eq. (10) of the main text. For an extended absorber region with the same length as the electrode gap (long lifetime photoconductor), the transit-time depends on generation point. For the photoconductor, the optical propagation is perpendicular to the transport direction. Eq. (S10) can again be separated into

$I_{PC;e,h}^{\tau_{rec}\gg}\left( t \right)=\int_{0}^{d} e\alpha\frac{P_{L,0}\left( z \right)}{h\nu}dz\cdot\frac{1}{w_{G}}\int_{0}^{w_{G}} \int_{t-t^{'}=0}^{t-t^{'}=\tau_{tr}(x)} \frac{v_{e,h}}{w_{G}}\cdot(1+\cos\omega t^{'})dt'dx$. (S21)

The term $\frac{1}{w_{G}}$ before the second integral originates from the spatial dependence of the generation rate $g\left( \vec{r},t^{'} \right)$, which in turns is determined by the optical intensity, $I_{L}\left( \vec{r},t \right)=\frac{P_{L}\left( \vec{r},t \right)}{Ww_{G}}$. The width $W$ has vanished as the integral along the y-axis was already carried out. The fist integral is again the ideal photocurrent, and the temporal integral results in Eq. (S20), however, the x-integration has now to be carried out as well, where we use $\tau_{tr,e,h}\left( x \right)=x/v_{e,h}$,

$I_{PC;e,h}^{\tau_{rec}\gg}\left( t \right)=I_{id}\cdot\frac{1}{w_{G}}\int_{0}^{w_{G}} \frac{x}{w_{G}}+\mathrm{sinc}\left( \frac{\omega x}{2v_{e,h}} \right)\cos\left( \omega t+\frac{\omega x}{2v_{e,h}} \right)dx$, (S22)

resulting in

$I_{PC;e,h}^{\tau_{rec}\gg}\left( t \right)=\frac{1}{2}\left( I_{id}+\frac{I_{id}}{\xi}\sqrt{\sin^{4}\left( \xi\right)+\left( \xi-\frac{1}{2}\sin\left( 2\xi\right) \right)^{2}}\cdot\cos\left( \omega t+\varphi\right) \right)$, (S23)

where $\xi=\frac{\omega\tau_{tr,e,h}^{max}}{2}$ and $\tau_{tr,e,h}^{max}=\frac{w_{G}}{v_{e,h}}.$ This is the solution to Eq. (11) in the main text. The DC amplitude is the same for electrons and holes, in agreement with Eqs. (S3) and (S4) as the center of mass of the charges is in the middle of the absorber. That is, electrons and holes contribute equally to the DC current. The situation differs for the AC part, where the roll-off of the slower carrier type, usually the hole, is more severe and the terahertz current is therefore dominated by the faster charge type.

For p-i-n diodes under illumination from the top, the solution is more complex as the optical flux is along the absorber layer parallel to the transport direction, i.e. both in *z*-direction. Therefore, the power is attenuated according to the Lambert-Beer law. Eq. (S10) turns into

$I_{PIN}^{\tau_{rec}\gg}\left( t \right)=\int_{0}^{d} e\alpha\frac{P_{L,0}\left( z \right)}{h\nu}\int_{t-t^{'}=0}^{t-t^{'}=\tau_{tr}(z)} \frac{v}{d^{2}}\cdot(1+\cos\omega t^{'})dt'dz$. (S24)

The explicit z-dependence of the transit time does complicate the calculation. Although we leave the general solution to the reader, an approximate solution can be obtained under the assumption that the absorber layer is comparatively short. This is indeed a decent assumption for terahertz p-i-n diodes as the absorber has to be comparatively short in order to prevent a severe transit-time roll-off. Typical lengths of absorber plus transport layer are of the order of 200-400 nm, yielding an absorption on the order of 20%-30% even if the whole transport layer absorbs. For UTC diodes, the absorber is even smaller, of the order of 100 nm. Therefore, neglecting the spatial variation of the absorbed power due to Lambert-Beer’s law will only have a minor effect on the final result, hence we assume that the absorbed power is given by ${P_{L,abs}=\alpha dP}_{L,0},$ where $d$ is the thickness of the absorber layer. The ideal photocurrent is thus approximately $I_{id}\approx\alpha d\frac{eP_{L,0}}{h\nu}$ and the solution of Eq. (S24) is identical to Eq. (S23) with $w_{G}\equiv d$.

3.) Ultrashort pulsed excitation, $g\left( \vec{r},t^{'} \right)= \alpha\cdot\frac{E_{L,0}(z)\delta(t^{'})}{Ww_{G}\cdot h\nu}$, where $E_{L,0}$ is the laser pulse energy. This final case discusses pulsed photoconductive sources where we assume, for simplicity, an ultrashort laser pulse. The corresponding answer thus solely depends on device features. In a realistic scenario, however, the pulse will have a finite pulse width and a certain repetition rate, resulting in a discrete spectral structure [2]. For the simplistic case treated here, the temporal integral breaks down due to the delta function

$I_{Pls}\left( t \right)=\int_{0}^{d} e\alpha\frac{E_{L,0}\left( z \right)}{h\nu}dz\cdot\frac{1}{w_{G}}\int_{0}^{w_{G}} e^{-\frac{t}{\tau_{rec}}}\cdot\frac{v}{w_{G}}\cdot\Theta\left( t \right)\Theta(\tau_{tr}\left( x \right)-t)dx$ (S25)

where we have taken the geometry of a photoconductor with electrode spacing $w_{G}$ and thickness $d$. The pulse energy decays again according to Lambert-Beer law as $E_{L,0}\left( z \right)=E_{L,0}\exp(-\alpha z).$ The first integral delivers the total (ideal) charge generated by the pulse, $Q_{id}=e\frac{E_{L,0}}{h\nu}\exp\left( -\alpha d \right)$, while the second integral the temporal dynamics, as given by Eq. (13) of the main manuscript.

**References**

1. S. Preu, G. H. Döhler, S. Malzer, L. Wang, and A. C. Gossard, “Tunable, continuous-wave terahertz photomixer sources and applications,” J. Appl. Phys. 109, 061301 (2011).
2. A.D.J. Fernandez Olvera, B.L. Krause, A. Betancur-Perez, U. Nandi, C. de Dios, P. Acedo, and S. Preu “Frequency Selective Optoelectronic Downconversion of a Terahertz Pulse Using ErAs:In(Al)GaAs Photoconductors,” IEEE Access. 9, (2021).
