# Supplementary material for: Ultrafast carrier dynamics in terahertz photoconductors and photomixers: beyond short-carrier-lifetime semiconductors
Source: Nanophotonics. 2022 Mar 10;11(11):2661–91. doi: 10.1515/nanoph-2021-0785 (PMC11501130; doi:10.1515/nanoph-2021-0785)
Supplement: Supplementary file 2 — Supplementary Material [file j_nanoph-2021-0785_suppl_002.pdf]

Ultrafast Carrier Dynamics in Terahertz Photoconductors and Photomixers: beyond Short-Carrier-Lifetime Semiconductors

Ping-Keng Lu[1,*], Anuar de Jesus Fernandez Olvera[2], Deniz Turan[1], Tom Sebastian Seifert[3], Nezih Tolga Yardimci[1], Tobias Kampfrath[3,4], Sascha Preu[2], Mona Jarrahi[1]

[1] Electrical and Computer Engineering Department, University of California, Los Angeles, CA, USA.

[2] Department of Electrical Engineering and Information Technology, Technical University Darmstadt, Darmstadt, Germany.

[3] Department of Physics, Freie Universität Berlin, 14195 Berlin, Germany.

[4] Department of Physical Chemistry, Fritz Haber Institute of the Max Planck Society, 14195 Berlin, Germany.

* Corresponding author: pklu@ucla.edu

Abstract: Efficient terahertz generation and detection are a key prerequisite for high performance terahertz systems. Major advancements in realizing efficient terahertz emitters and detectors were enabled through photonics-driven semiconductor devices, thanks to the extremely wide bandwidth available at optical frequencies. Through the efficient generation and ultrafast transport of charge carriers within a photo-absorbing semiconductor material, terahertz frequency components are created from the mixing products of the optical frequency components that drive the terahertz device – a process usually referred to as photomixing. The created terahertz frequency components, which are in the physical form of oscillating carrier concentrations, can feed a terahertz antenna and ~~radiate~~get radiated in case of a terahertz emitter, or mix with an incoming terahertz wave to down-convert to DC or to a low frequency photocurrent in case of a terahertz detector. Realizing terahertz photoconductors typically ~~rely~~relies on short-carrier-lifetime semiconductors as the photo-absorbing material, where photocarriers are quickly trapped within one picosecond or less after generation, leading to ultrafast carrier dynamics that ~~facilitate~~ facilitates

high-frequency device operation. However, while enabling broadband operation, a sub-picosecond lifetime of the photocarriers results in a substantial loss of photoconductive gain and optical responsivity. In addition, growth of short-carrier-lifetime semiconductors ~~often~~in many cases relies on the use of rare elements and non-standard processes with limited accessibility. Therefore, there is a strong motivation to explore and develop alternative techniques for realizing terahertz ~~photomixer~~photomixers that do not rely on these defect-introduced short-carrier-lifetime semiconductors. This review will provide an overview of several promising approaches to realize terahertz emitters and detectors without short-carrier-lifetime semiconductors. These novel approaches utilize p-i-n diode junctions, plasmonic nanostructures, ultrafast spintronics, and low-dimensional materials to offer ultrafast carrier response. These innovative directions have great potentials for extending the applicability and accessibility of the terahertz spectrum for a wide range of applications.

1. Introduction

The terahertz frequency range is loosely defined as the part of the electromagnetic spectrum between 100 GHz and 10 THz, which is above microwave and below infrared frequencies. Due to the presence of rotational resonances and collective librations of various polar molecules, the terahertz frequency range is widely explored for a plethora of applications including chemical sensing and material characterization, where highly frequency-selective absorption signatures provide ample information about the identity and physical characteristics of the sample under test [1-4]. In addition, terahertz waves can transmit through many optically opaque materials such as paper, plastics, textiles, as well as weakly doped semiconductors [5-9]. Therefore, terahertz radiation is well-suited for imaging and sensing applications in many optically inaccessible environments [10-13]. With photon energies below 40 meV, terahertz waves are non-ionizing and, hence, suited for many biomedical imaging, diagnosis and nondestructive quality inspection [14-22].

Generation and detection of terahertz radiation have been extensively realized through ultrafast photoconductors, which translate the mixing products of the frequency components of an optical pump beam to a terahertz photocurrent. (Fig. 1). A common implementation - the so-called photoconductive antenna - uses a semiconductor-based photoconductor integrated with a metallic terahertz antenna. When photons with an energy above the semiconductor's band gap are absorbed by the semiconductor, mobile electron-hole pairs are generated. The concentration of these photocarriers oscillates at the beat terahertz frequencies that are generated from the mixing of the incident optical frequency components. These photocarriers are accelerated under an electric field and form a photocurrent. This electric field can be supplied through an external bias voltage or a built-in field in the semiconductor. The electric field should be strong enough to drift the photocarriers at high velocities in order to attain a large photocurrent amplitude, which directly translates into strong terahertz radiation that is emitted by the antenna. The scheme can also be reversed to detect terahertz radiation: the antenna attached to a photoconductor receives the terahertz electric field that accelerates the pump-induced photocarriers. The resulting photocurrent is proportional to the convolution of the terahertz field and the laser-induced photoconductivity. Photoconductive terahertz emitters and detectors are utilized in pulsed or continuous-wave (CW) operation. For pulsed operation, a femtosecond laser is typically used as the optical pump source to generate and detect sub-picosecond terahertz pulses with a spectral width of several THz. For CW operation, two CW lasers with the same polarization and a terahertz frequency difference are superimposed to create an optical pump beam with a single-frequency terahertz beatnote that is used for the generation and detection of CW terahertz radiation.

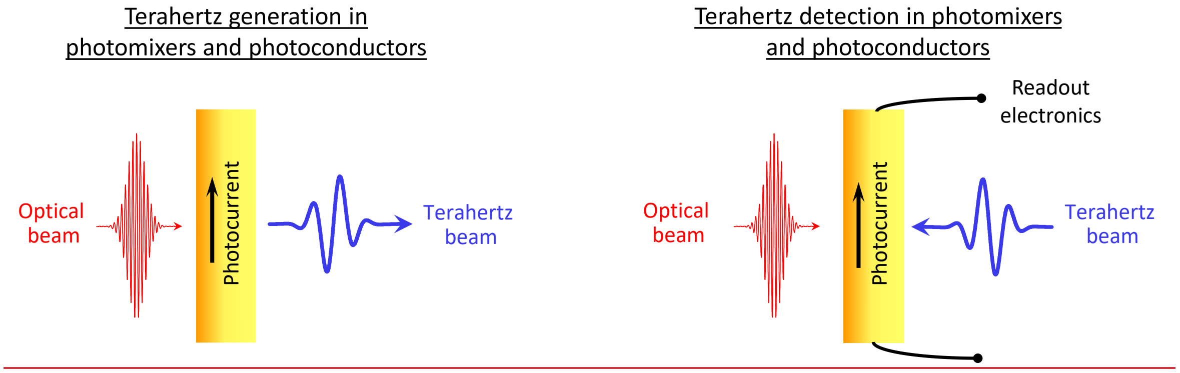

Figure 1: Terahertz generation and detection in photomixers and photoconductors.

Ultrafast dynamics of the photocarriers, with sub-picosecond to picosecond response times, are necessary to facilitate the broadband operation of terahertz photoconductors. A common approach to fulfill this requirement is to use short-carrier-lifetime semiconductors. Low-temperature-grown GaAs (LT-GaAs) was the first, and is still one of the most commonly used short-carrier-lifetime ~~semiconductor used~~semiconductors for terahertz ~~photoconductors operating~~generation and detection when working with an ~800 nm optical pump wavelength [23-26]. Growing GaAs at a low temperature (200-350 °C) and subsequent in-situ annealing at higher temperatures [24-26] induces crystal defects due to excess arsenic, which ~~capture~~captures the photocarriers within less than one picosecond. Following the same concept of defect introduction, a diversity of methods for growing short-carrier-lifetime photo-absorbing semiconductors at various optical excitation wavelengths ~~has~~have been introduced [27-34]. In particular, short-carrier-lifetime semiconductors with high photo-absorption at telecommunication wavelengths (~1550 nm) are of strong interest. This is because the combination of terahertz photoconductors with small-footprint ~~and~~together with highly reliable fiber lasers and fiber-optic components ~~lead to~~results in low-cost and compact terahertz systems. In order to create recombination centers within the semiconductor lattice, some of the techniques used for introducing defects in photo-absorbing semiconductors at ~1550 nm wavelength include incorporating rare-earth elements [35-37~~] and~~], transition metal doping [38-40] and low-temperature-grown InGaAs/InAlAs multilayer heterostructures [41-43].

While ~~most of the terahertz photoconductors are realized with~~many impressive results have been demonstrated using short-carrier-lifetime ~~semiconductors~~photoconductors, their ultrafast carrier dynamics comes with several tradeoffs in photoconductive gain, optical responsivity, carrier mobility, and thermal conductivity. In particular, due to the sub-picosecond lifetime of the photocarriers, a substantial fraction of ~~the photocarriers~~them is trapped and then recombined before contributing to the generation and detection of terahertz radiation, degrading the efficiency (i.e. the gain) of terahertz photoconductors. Also, while some growth methods for short-carrier-lifetime semiconductors like LT-GaAs are well-established processes performed by many ~~of the~~groups, most growth methods for short-carrier-lifetime semiconductors utilize non-standard processes and, in some cases rare dopant elements, that are not readily available in most molecular beam epitaxy (MBE) and metal organic chemical vapor deposition (MOCVD) facilities, limiting their accessibility and widespread usage. In addition, these methods have to be adapted for each specific semiconductor, limiting the materials and optical wavelengths that can be used for realizing terahertz photoconductors. These drawbacks have motivated the ~~realization~~emergence of alternative terahertz photoconductor and photomixer concepts that do not rely on short-carrier-lifetime semiconductors.

Rather than carrier lifetime reduction, ultrafast carrier dynamics can be realized through alternative approaches including carrier transit time reduction or utilizing novel materials with unique carrier transport properties. For example, the relatively slow hole current is suppressed in the active region of uni-traveling carrier photodiodes (UTC-PDs), leading to short carrier transit times dominated by the high-mobility electrons [44-46]. Incorporating optical cavities and plasmonic contact electrodes in terahertz photoconductors is another way to achieve short carrier transit times [47-49]. By exciting surface plasmon waves at the interface between the semiconductor substrate and the metallic nanostructures that simultaneously serve as the contact electrodes, the photocarrier concentration is significantly enhanced near the metallic nanostructures, leading to greatly reduced transport distances for the photocarriers.

This concept has been successfully applied to a diversity of terahertz emitters and detectors under both pulsed and CW operation [50-54]. In addition, the recently demonstrated spintronic terahertz emitters are based on metallic thin films that rely on optically induced ultrafast spin and charge dynamics for terahertz generation [55, 56]. By stacking a ferromagnetic (FM) material and a non-ferromagnetic (NM) material with strong spin-orbit coupling, an ultrafast spin current is generated upon femtosecond laser excitation of the FM layer. The spin current is transmitted through the FM/NM interface and is converted into an ultrafast charge current inside the NM material, which radiates broadband terahertz waves. Alternatively, the unique electrical and optical properties of many low-dimensional materials make them attractive alternatives to short-carrier-lifetime semiconductors used in terahertz photoconductors [57-59]. On the one hand, the broad optical absorption spectrum of many low-dimensional materials enable operation at a broad range of optical pump wavelengths. On the other hand, the very high carrier mobility and ultrafast carrier relaxation of many of these materials, such as graphene, can provide carrier response times in the sub-picosecond to picosecond range. Figures 2a-2d illustrate the physical mechanisms enabling ultrafast carrier dynamics in each of the aforementioned scenarios: p-i-n diode junctions, plasmonic nanostructures, spintronics, and low-dimensional materials for terahertz generation and detection.

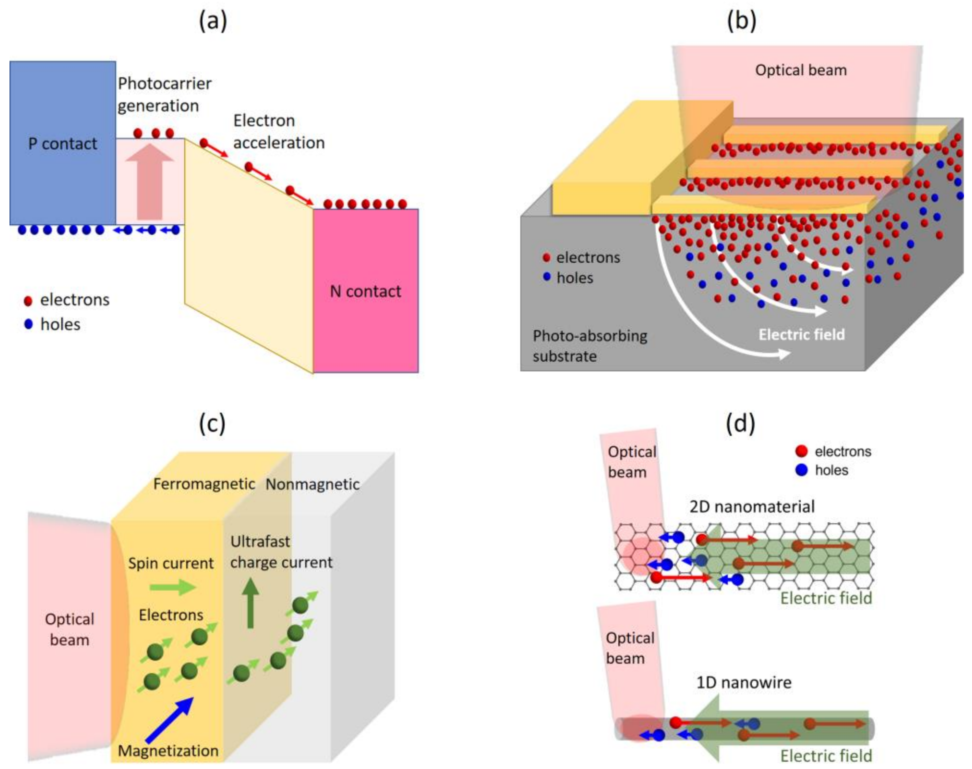

Figure 2: Schematics that illustrate the ultrafast carrier dynamics enabling terahertz generation and detection. (a) Uni-traveling carrier photodiode junctions, where electron-dominant carrier transport enables high-power CW terahertz generation. (b) Plasmonic nanostructures, where plasmonics-enhanced near-field photocarrier generation enables pulsed and CW terahertz generation and detection. (c) Spintronic materials, where ultrafast electronic spin-to-charge conversion enables broadband terahertz generation. (d) Low-dimensional materials, where high mobility photocarrier transport enables terahertz generation and detection.

This review ~~paper~~article aims to provide an overview of the above-mentioned approaches for realizing ultrafast carrier dynamics in terahertz photoconductors and photomixers without using defect-introduced short-carrier-lifetime semiconductors. Section 2 provides the theoretical foundation of the generation

and detection of terahertz radiation through terahertz photoconductors and ~~photodiodes~~photomixers. Terahertz photoconductors and photomixers utilizing the ultrafast carrier dynamics in p-i-n diode junctions, as well as plasmonic nanostructures, spintronic materials, and low-dimensional materials are described in Section 3. Finally, the conclusions and the outlook are given in Section 4.

2. Theory of terahertz photoconductors and photomixers

This section introduces ~~a revised theory of~~an updated theoretical model of the terahertz generation and detection process in photoconductors and photomixers based on the theory presented in [60~~] with a generalized model~~, 61]. This updated theoretical model is a generalization for any ~~arbitrary material~~kind of generation geometry (i.e. not limited to a specific region of the photoconductor) and any kind of optical pump signal. A detailed elaboration is presented in the supplemental material.

Photocarrier generation and transport

When electron-hole pairs are excited in a semiconductor by an optical pump signal with a photon energy higher than the semiconductor bandgap energy, $h\nu \geq$ ~~E_G~~E_{G}, and a power of ~~P_L~~$P_{\mathrm{L}}(t)$, electrons are ~~excited~~raised to the conduction band and holes are created in the valence band. After generation, they diffuse or drift within the semiconductor until they are eventually either trapped within their average carrier lifetime, ~~τ_{rec}~~τ_{rec}, or reach their respective electrode to generate an external current. We first look at a microscopic picture to calculate the infinitesimal currents generated locally within the device that is finally integrated up over the whole absorption volume in order to yield the total photocurrent. Figure ~~1~~3 illustrates the general device geometry and variables used in the subsequent equations.

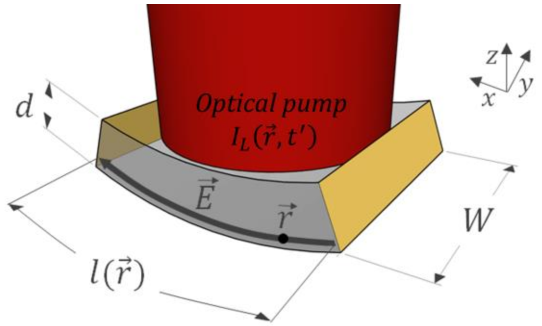

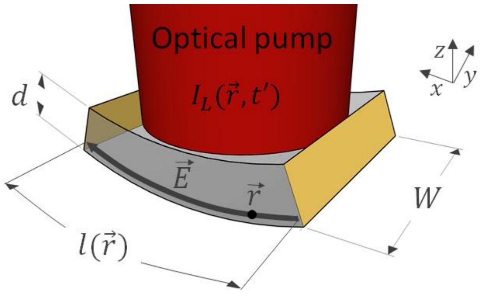

Figure ~~1~~3: The optical pump signal with an intensity ~~I_L~~$I_{\mathrm{L}}\left(\vec{r},t'\right)$ is incident from the top on a photoconductor (or p-i-n diode) and absorbed along an absorption length *d*. Yellow: contact electrodes.

The carriers are generated by the incident optical pump at a time t' at a generation rate of

$$g(\vec{r},t') = \alpha(\vec{r},\nu) \cdot \frac{I_{\mathrm{L}}(\vec{r},t')}{h\nu}, \qquad (1)$$

where $\alpha(\vec{r},\nu)$ is the laser frequency- and spatially-dependent absorption coefficient. For the case of plasmonic photoconductors (see section 3.B), a spatial dependence of α comes from the enhancement of the absorption close to the metal electrodes. ~~I_L~~$I_{\mathrm{L}}(\vec{r},t')$ is the optical intensity entering the semiconductor. Reflection at the semiconductor surface is neglected here but can be easily considered by the transmission coefficient $T = 1 - R$. For planar devices with a constant cross~~-section-~~sectional area A, the intensity reads ~~$I_L(\vec{r},t')$~~$I_{\mathrm{L}}(\vec{r},t') =$ ~~$P_L(\vec{r},t')$~~$P_{\mathrm{L}}(\vec{r},t')/A$. Initially, the optically generated charges are at rest. Prone to an external field or a built-in field, the charges are accelerated towards their respective electrode. At a time point $\tau = t - t'$ after their generation at point $\vec{r}$ and time t', they have reached an (ensemble average) velocity of $\vec{\mathrm{v}}(\vec{r},\tau)$. The details of the evolution of the velocity will be discussed later. For now, we would like to provide a very general picture of the transport process. However, we remark that the velocity does not only have an explicit dependence on the generation point $\vec{r}$ but also an implicit one in the most general case, given by the electric field strength on the field line the charge is travelling on. As the field lines represent lines of constant electric field, the implicit dependence can be mapped onto the time dependence on τ. While being transported, carriers are trapped, exponentially reducing the carrier concentration [~~61~~62] as ~~$\exp[-\tau/\tau_{rec}]$~~$\exp[-\tau/\tau_{\mathrm{rec}}]$. In summary, the current density generated within a certain volume of the photoconductor can be written as

$$\vec{j}(\vec{r},t,t') = en(\vec{r},t,t')\vec{\mathrm{v}}(\vec{r},t,t') = e\alpha(\vec{r},\nu)\cdot\frac{I_{\mathrm{L}}(\vec{r},t')}{h\nu}\vec{\mathrm{v}}(\vec{r},t-t')\exp\left[-\frac{t-t'}{\tau_{\mathrm{rec}}}\right]. \tag{2}$$

The total current density requires integration over all generation times up to the maximum transport time ~~τ_{tr}~~$\tau_{\mathrm{tr}}(\vec{r})$ that a charge travels from its generation point $\vec{r}$ to its respective electrode or trapping site. Writing the integration boundaries as Heavyside step functions, the total current density generated at point $\vec{r}$ is

$$\vec{j}(\vec{r},t) = \int_{-\infty}^{\infty} e\alpha(\vec{r},\nu) \cdot\frac{I_{\mathrm{L}}(\vec{r},t')}{h\nu}\vec{\mathrm{v}}(\vec{r},t-t')\exp\left[-\frac{t-t'}{\tau_{\mathrm{rec}}}\right]\theta(t-t')\theta(\tau_{\mathrm{tr}}(\vec{r})-(t-t'))dt' \tag{3}$$

The current density can now be re-expressed as

$$\vec{j}(\vec{r},t) = \vec{G}(\vec{r},t) * g(\vec{r},t), \tag{4}$$

where "*" represents the convolution operator. Eq. (4) holds for any type of optical excitation $g(\vec{r},t)$, pulsed or CW, where

$$\vec{G}(\vec{r},t) = e\vec{\mathrm{v}}(\vec{r},t)\exp\left[-\frac{t}{\tau_{\mathrm{rec}}}\right]\theta(t)\theta(\tau_{\mathrm{tr}}(\vec{r})-t) \tag{5}$$

is the Green's function for the current density for any kind of photomixer or photoconductor with known carrier lifetime ~~τ_{rec}~~τ_{rec} and velocity profile $\vec{\mathrm{v}}(\vec{r},t)$. Finally, the total current generated within the device requires integration over all places where charges are flowing, namely the volume V,

$$I(t) = \int_V \frac{1}{l(\vec{r})}\vec{j}(\vec{r},t)\cdot\vec{n}_E(\vec{r})dV. \tag{6}$$

where $\vec{n}_E(\vec{r})$ is the unit vector of the direction of the field accelerating the charges that were generated at point $\vec{r}$, and $l(\vec{r})$ is the length of the corresponding field line. The normalization by the length of the

field line $l(\vec{r})$ is explained in detail in the supplemental material. An alternative way to write Eq. (6) is $\int \frac{1}{l} \int \vec{j}(\vec{r},t) \cdot d\vec{A}\, dl$, where the inner integral is the usual relation between current and current density. The outer integral is necessary to take the missing third dimension of the charge generation volume into account. We remark that Eq. (6) does not account for any high power effects, such as bias field screening and radiation field screening. Both effects will impact the effective accelerating field on a sub-picosecond time scale, requiring dynamic simulations.

As the transport kinetics of electrons and holes is generally different, Eq. (6) has to be solved separately for both partial currents. Under many circumstances, however, one of the two currents, often that of the electrons, is dominant for terahertz generation and detection. In general, Eqs. (3)-(6) hold for any type of optical excitation and for any type of photomixer or photoconductor. Solving Eq. (6) usually requires numerical methods, e.g. for the cases of a photoconductor with planar electrodes on the photoconductor surface featuring an inhomogeneous electric field distribution within the sample, a plasmonic photoconductor with strongly altered, inhomogeneous optical intensity distribution, or a p-i-n diode with ballistic velocity effects. Some simplified cases for illustrative purposes will be presented later.

Finally, the velocity profile needed for the Green's function in Eq. (5) deserves a closer look. Within the terahertz-relevant time scale of the first few 100 fs after their generation, the carriers start at rest and are then accelerated by an external or internal electric field, $\vec{E}(\vec{r})$. This acceleration process is not an equilibrium process, the charges are travelling mostly at velocities strongly differing from the drift velocity given by $\vec{\mathrm{v}} = \mu\vec{E}$. For defect-induced short carrier lifetime and indirect bandgap semiconductors, as well as for holes in general, the carrier velocity is often well described by the Drude-model,

$$m^* \frac{\partial \vec{\mathrm{v}}(\vec{r},t)}{\partial t} + m^* \frac{\vec{\mathrm{v}}(\vec{r},t)}{\tau_{\mathrm{sc}}} = e\vec{E}(\vec{r}) \qquad (7)$$

where m^* is the effective mass (for direct bandgap semiconductors, in the Γ-valley, for indirect bandgap semiconductors in their respective lowest valley). In photoconductive emitters and p-i-n diodes, the direction of the electric field changes only slowly (or not at all) along the propagation path of the charge, i.e. the field line. That is, only little energy is used for changing the direction of motion, while the vast majority is used for accelerating or maintaining the velocity of the ~~charge carrier~~carriers. This allows to approximate Eq. (7), at least locally, by its scalar form where all vectors are replaced by their respective scalar quantities. Under this simplifying assumption, Eqs. (6) and (7) decouple and the charge velocity from Eq. (7) can be reduced to a one-dimensional problem~~.~~, where $|\vec{E}(\vec{r})|$ is the field strength at the point of charge generation that remains constant along the field line the charge is travelling on. The scattering time can be approximated from the DC Hall mobility ~~μ_H~~μ_{H} as ~~τ_{sc}~~$\tau_{\mathrm{sc}} =$ ~~$\frac{m^*\mu_H}{e}$~~$\frac{m^*\mu_{\mathrm{H}}}{e}$. We remark that the scattering time under illumination and under bias may be somewhat shorter than the scattering time obtained from DC Hall measurements under dark conditions as the charges see more scattering partners and the device is hotter.

For p-i-n diodes or long carrier lifetime (~ns) photoconductors that are made of direct bandgap semiconductors, however, both the scattering time ~~τ_{sc}~~τ_{sc} and the effective mass m^* cannot be considered constant for electrons: the motion of electrons starts in the Γ-valley, where they feature a comparatively small effective mass ($0.067m_0$ for GaAs and $0.041m_0$ for InGaAs, e.g. [~~62~~63]) and are then accelerated ballistically, i.e. with negligible scattering. Thus, the second term in Eq. (7) can be neglected. After the electrons reach the LO phonon energy (typically a few 10 meV), they start ~~efficiently~~ emitting phonons efficiently. But this effect is comparatively weak such that the electrons are further accelerated, yet with a somewhat reduced acceleration [~~63~~64]. Finally, they reach a kinetic energy corresponding to the energy difference between the Γ-valley and the next higher valley, which enables very efficient

scattering to the respective side valleys. At this point, they may have reached a velocity ten times higher than the saturation velocity [60]. The side valley scattering has two major consequences: (i) the effective mass in the side valley ~~m_S^*~~m_{S}^* is usually much larger than in the Γ-valley (about 13 times higher in GaAs and about 7 times higher in $In_{0.53}Ga_{0.47}As$) strongly slowing down the acceleration~~.~~, and (ii) there are several side valleys in different directions where the electrons can scatter to. This effectively randomizes the direction of motion, slowing down the average velocity to the respective drift velocity, ~~$\vec{v}_D$~~$\vec{\mathrm{v}}_{\mathrm{D}} =$ ~~μ_H~~$\mu_{\mathrm{H}}\vec{E}$. The effect of reaching velocities much higher than the saturation velocity is termed as ballistic transport or, likewise, velocity overshoot. It plays a dominant role in all direct bandgap semiconductor p-i-n diode concepts that make use of electron transport only, such as uni-travelling carrier photodiodes (see section 3.A). For such complex transport kinetics, simulations are typically indispensable. Further details can be found in [60] and in [~~63~~64]. For typical acceleration fields and semiconductor materials used in photoconductive terahertz emitters, ballistic effects last only for the first ~200 fs [~~63~~64], depending on the applied bias field and semiconductor parameters. However, this is the time scale most relevant for terahertz generation.

Simple cases with analytical solutions

To conclude the theoretical description, let us examine ~~a few~~some simple cases where analytical solutions of Eqs. (3)-(6) exist. We assume a uniform optical excitation and a decay of the optical pump power according to the Lambert-Beer's law along the light propagation direction. We also assume carrier transport with a constant velocity v in a homogeneous field, e.g. in a plane-parallel electrode layout [~~64~~65] with an electrode spacing of w_G. Under these approximations, all field lines have the same length, i.e., $l(\vec{r}) =$ ~~$w_G = const$~~$w_{\mathrm{G}} = \mathrm{const}$.

1) Short carrier lifetime photoconductor pumped by a CW optical excitation under a DC electric field (applied externally or internally) operating as a CW emitter. In this scenario, ~~τ_{rec}~~$\tau_{\mathrm{rec}} \ll$ ~~τ_{tr}~~$\tau_{\mathrm{tr}} = \frac{r}{\mathrm{v}}$ for almost all generation points, $g(\vec{r},t') = \alpha \cdot$ ~~$\frac{P_{L,0}(z)(1+\cos\omega t')}{A\cdot h\nu}$~~$\frac{P_{\mathrm{L},0}(z)(1+\cos\omega t')}{A\cdot h\nu}$ and the current in Eq. (6) becomes [~~61~~62]

$$I_{\mathrm{PC}}(t) = \frac{eP_{\mathrm{L,abs}}}{h\nu}\cdot\frac{\mathrm{v}\tau_{\mathrm{rec}}}{w_{\mathrm{G}}}\cdot\left(1+\frac{1}{\sqrt{1+\omega^2\tau_{\mathrm{rec}}^2}}\cos(\omega t+\varphi)\right), \tag{8}$$

where $\varphi =$ ~~$\arctan\omega\tau_{rec}$~~$\arctan\omega\tau_{\mathrm{rec}}$, ~~$\frac{v\tau_{rec}}{w_G}$~~$\frac{\mathrm{v}\tau_{\mathrm{rec}}}{w_{\mathrm{G}}} =$ ~~$\frac{\tau_{rec}}{\tau_{tr}^{max}}$~~$\frac{\tau_{\mathrm{rec}}}{\tau_{\mathrm{tr}}^{\mathrm{max}}} = g$ is termed as the photoconductive gain, and ~~η_{LT}~~$\eta_{\mathrm{LT}} =$ ~~$\frac{1}{\sqrt{1+(2\pi\tau_{rec}f)^2}}$~~$\frac{1}{\sqrt{1+\omega^2\tau_{\mathrm{rec}}^2}}$ is defined as the lifetime roll-off. It should be noted that the radiated power is proportional to the square of the AC current and hence rolls of as f^{-2} at high frequencies. The factor containing the absorbed fraction of the optical pump power, ~~$I_{id} = \frac{eP_{L,abs}}{h\nu} = \frac{eP_{L,0}}{h\nu}$~~$I_{\mathrm{id}} = \frac{eP_{\mathrm{L,abs}}}{h\nu} = \frac{eP_{\mathrm{L},0}}{h\nu}[1-\exp(-\alpha d)]$, is the ideal photocurrent that is only achievable with long carrier lifetime photoconductors at low frequencies.

2) Long carrier lifetime photoconductor or p-i-n diode pumped by a CW optical excitation under a DC electric field (applied externally or internally) operating as a CW emitter. In this scenario, the long carrier lifetime allows to drop the exponential term in Eq. (5~~)~~), resulting in

$$j(\vec{r},t) = e\alpha\cdot\frac{P_{\mathrm{L},0}(z)}{A\cdot h\nu}\mathrm{v}\tau_{\mathrm{tr}}\left(1+\mathrm{sinc}\frac{\omega\tau_{\mathrm{tr}}}{2}\cos\left(\omega t+\frac{\omega\tau_{\mathrm{tr}}}{2}\right)\right). \tag{9}$$

~~It~~Eq. (9) has to be evaluated separately for electrons and holes in case of photoconductors. In case of uni-travelling carrier diodes, where charges are essentially generated at a very localized position close to the p-contact, the hole current can be neglected and the total current is given by the current generated by electrons as

$$I_{\mathrm{UTC}}(t) = I_{\mathrm{id}}\left(1 + \mathrm{sinc}\frac{\omega\tau_{\mathrm{tr}}}{2}\cos\left(\omega t + \frac{\omega\tau_{\mathrm{tr}}}{2}\right)\right), \tag{10}$$

where we have used v~~τ_{tr}~~$\tau_{\mathrm{tr}} =$ ~~w_G~~$w_{\mathrm{G}} = l$. The zero crossings of the sinc term of the AC current will not be visible in real devices as the finite absorption area and the non-constant velocity of the charges smear it out. Some waviness, however, may remain. The envelope of the sinc term causes a linearly increasing loss of the AC current with increasing frequency above a 3 dB frequency roughly determined as ~~$f^{tr}_{3dB} \approx \frac{1}{b\tau_{tr}}$~~$f^{\mathrm{tr}}_{\mathrm{3dB}} \approx \frac{1}{b\tau_{\mathrm{tr}}}$, with $b \approx 2$ depending on the details of the carrier transport [60]. Therefore, the terahertz power (being proportional to the square of the AC current amplitude) decreases roughly as f^{-2} above this frequency. Independent of the type of diode or long carrier lifetime photoconductor, the transit-time roll-off is usually approximated as ~~η_{tr}~~$\eta_{\mathrm{tr}} = \frac{1}{\sqrt{1+(b\tau_{\mathrm{tr}}f)^2}}$, again with $b \approx 2$.

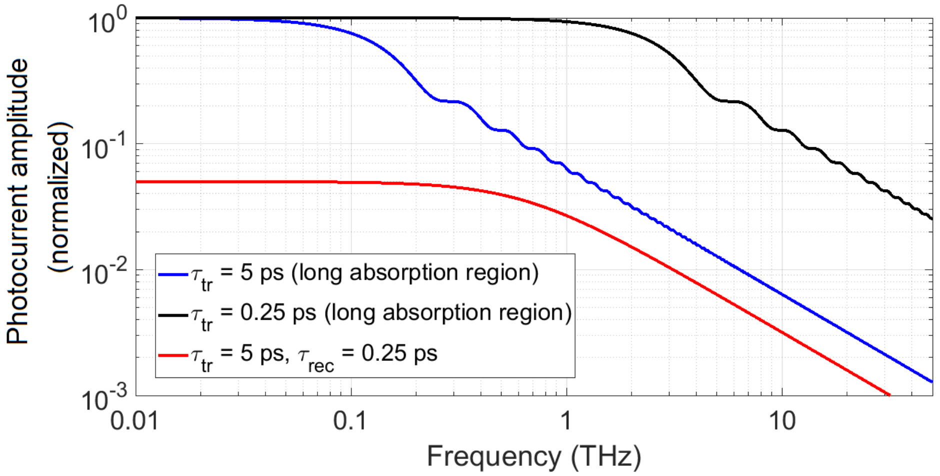

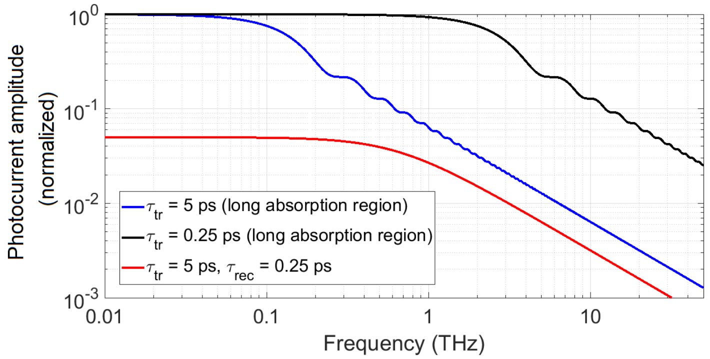

Figure ~~2~~4: Amplitude of the (electron) AC photocurrent as a function of frequency for three different cases: a long-carrier-lifetime photoconductor with $\tau_{\mathrm{tr}}^{\max} = 5$ ps, a long carrier-lifetime photoconductor with a sub-ps transit-time ~~τ_{tr}~~$\tau_{\mathrm{tr}}^{\max} = 0.25$ ps, and a short-carrier-lifetime photoconductor with a long transit time ~~τ_{tr}~~$\tau_{\mathrm{tr}}^{\max} = 5$ ps and a ~~τ_{rec} =~~$\tau_{\mathrm{rec}} =$ 0.25 ps. The amplitudes are normalized to the long lifetime cases. All cases show a f^{-1} AC current roll-off, corresponding to a f^{-2} power roll-off. The ~~low~~ short-carrier-lifetime material has a $g = 0.05$ times lower low frequency amplitude and a lower 3 dB frequency as the ~~τ_{tr}~~$\tau_{\mathrm{tr}} = 0.25$ ps long-carrier-lifetime case, but a higher one than the ~~τ_{tr}~~$\tau_{\mathrm{tr}} = 5$ ps case.

In case of long carrier lifetime photoconductors, like semi-insulating (SI) GaAs, or p-i-n diodes with extended absorber regions (of length $l =$ ~~$d_i = w_G)$~~ $d_\mathrm{i} = w_\mathrm{G})$~~,~~ currents are generated all over the ~~area~~transport region, yielding

$$I_{\mathrm{PC}}^{\tau_{\mathrm{rec}} \gg}(t) = \frac{1}{2} I_{\mathrm{id}} \left[1 + 2 \cdot \left(\frac{2}{\omega \tau_{\mathrm{tr}}^{\max}} \right)^2 \int_0^{\frac{\omega \tau_{\mathrm{tr}}^{\max}}{2}} \sin\vartheta \cdot \cos(\omega t + \vartheta)\, d\vartheta \right], \tag{11}$$

where ϑ is a unitless integration variable and ~~τ_{tr}^{max}~~ $\tau_{\mathrm{tr}}^{\max} =$ ~~$\frac{w_G}{v}$~~ $\frac{w_\mathrm{G}}{\mathrm{v}}$. The amplitude of the AC current component can be determined analytically as ~~A_{AC}~~ $A_{\mathrm{AC}}(\xi) =$ ~~$\frac{I_{id}}{2\xi^2}$~~ $\frac{I_{\mathrm{id}}}{2\xi^2} \sqrt{\sin^4(\xi) + \left(\xi - \frac{1}{2}\sin(2\xi) \right)^2}$ where $\xi =$ ~~$\frac{\omega\tau_{tr}^{max}}{2}$~~ $\frac{\omega \tau_{\mathrm{tr}}^{\max}}{2}$. It will differ for electrons and holes because they usually travel at different velocities and have different transit ~~-~~times. An example is shown in Fig. ~~2~~4 for ~~τ_{tr}^{max}~~ $\tau_{\mathrm{tr}}^{\max} = 5$ ps~~.~~ and 0.25 ps, respectively. Clearly, the nodes of the sinc have (almost) vanished. The factor $\frac{1}{2}$ in front of the DC term is due to the fact that for a long carrier lifetime photoconductor or a p-i-n diode each electron and each hole contribute on average just half of the ideal ~~current~~photocurrent as the center of mass is in the middle of the device. Summing up electron and hole currents yields again ~~I_{id}~~ I_{id}. We remark that Eq. (11) is only correct for photoconductors where light propagation and charge transport directions are orthogonal to each other. For p-i-n diodes, this is ~~generally~~frequently not the case, so Eq. (11) is only a good approximation for diodes with comparatively short absorber layers (absorption $\ll 50\%$). More details can be found in the supplemental materials.

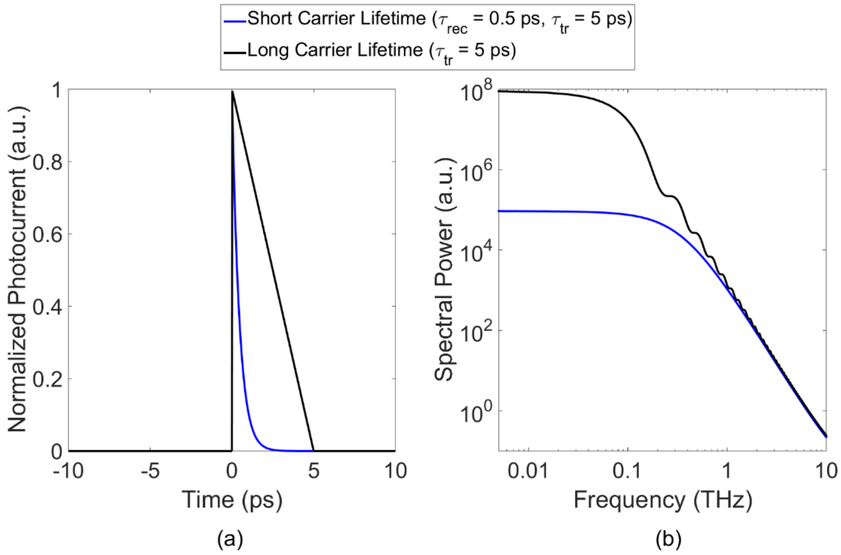

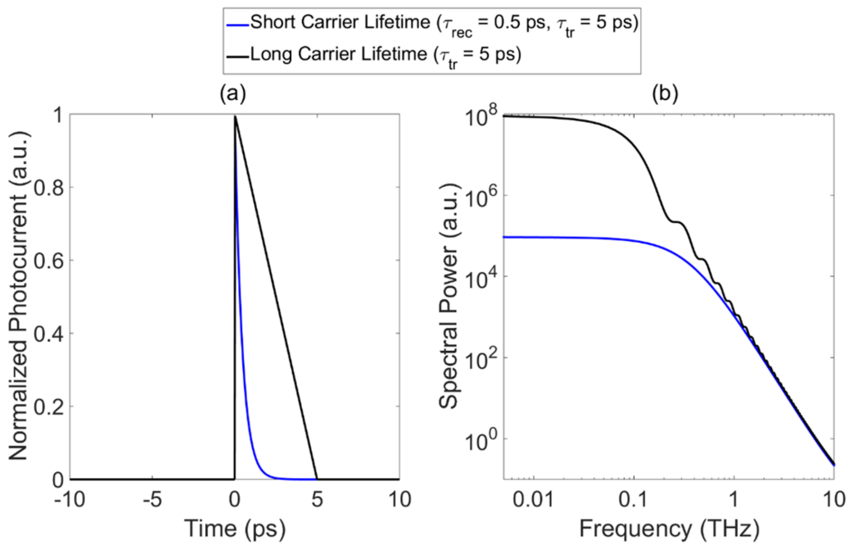

Figure ~~3~~5: Time domain trace of the photocurrent amplitude (a) and the power spectra (b) of a long carrier lifetime and a short carrier lifetime photoconductor under pulsed operation.

3) Photoconductor pumped by an ultrashort pulse optical excitation under a DC electric field (applied externally or internally) operating as a pulsed emitter. In this scenario, $g(\vec{r},t') = \alpha \cdot$ ~~$\frac{E_{L,0}(z)\delta(t')}{A \cdot h\nu}$~~ $\frac{E_{\mathrm{L},0}(z)\delta(t')}{A \cdot h\nu}$, where ~~$E_{L,0}$~~ $E_{\mathrm{L},0}(z)$ is the optical pulse energy, and the time integral in Eq. (3) yields

$$j(\vec{r},t) = e\alpha \cdot \frac{E_{\mathrm{L},0}(z)}{A \cdot h\nu} \mathrm{v} \cdot \exp\left[-\frac{t}{\tau_{\mathrm{rec}}}\right] \theta(t)\theta(\tau_{\mathrm{tr}}(\vec{r}) - t). \tag{12}$$

The current is the integral over all generation points, yielding a triangular shape for a long-carrier-lifetime photoconductor, while for a short-carrier-lifetime photoconductor it will essentially remain an exponentially decaying function,

$$I_{\mathrm{pls}}(t) = \frac{Q_{\mathrm{id}}}{\tau_{\mathrm{tr}}^{\mathrm{max}}} e^{-\frac{t}{\tau_{\mathrm{rec}}}} \left(1 - \frac{t}{\tau_{\mathrm{tr}}^{\mathrm{max}}}\right) \Theta(t)\Theta(\tau_{\mathrm{tr}}^{\mathrm{max}} - t), \tag{13}$$

where ~~Q_{id}~~ $Q_{\mathrm{id}} =$ ~~$\frac{eE_{L,abs}}{h\nu}$~~ $\frac{eE_{\mathrm{L,abs}}}{h\nu} = (1 - \exp(-\alpha d))$ ~~$\frac{eE_{L,0}}{h\nu}$~~ $\frac{eE_{\mathrm{L},0}}{h\nu}$ is the total charge generated by the optical pump. For a p-i-n diode or a photoconductor with a long carrier lifetime, the current density (Eq. 12) appears as a function with an abrupt onset, a duration of the charge transit time and then an abrupt end when the charge cloud originating from point $\vec{r}$ reaches the contact. The current turns into a saw tooth shape as shown in Fig. 5a. For a photoconductor with a short carrier lifetime, there will be an abrupt onset, followed by an exponential decay with the recombination time ~~τ_{rec}~~ τ_{rec} as also illustrated in Fig. ~~3a~~5a. In both cases, the spectrum will be dominated by the sharp onset of the current. As Eq. (13) does not depend explicitly on the photoconductive gain, a short carrier lifetime is not desperately necessary for terahertz generation in this scenario. The terahertz components are predominantly generated by the sharp current onset caused by the short laser pulse duration. The main difference between a short and a long-carrier-lifetime photoconductor is that the former decays more quickly to the dark state, while the latter and the p-i-n diode carry the generated charges for the whole transport time. That is, the DC current will be (much) higher, which may impose limits on maximum laser power before thermal destruction occurs. This feature is easy to see in the frequency domain for a short-carrier-lifetime material, where Eqs. (4), (5) and (6),

under the assumptions of a constant field and a constant carrier velocity, and for an arbitrarily shaped absorbed optical power $P^{\mathrm{abs}}_{~~P^{abs}_{L}~~\,\mathrm{L}}(t')$, yield

$$I(t) \approx \frac{1}{\tau_{\mathrm{tr}}^{\max}} \int_0^{\infty} \frac{eP_{\mathrm{L}}^{\mathrm{abs}}(t')}{h\nu} e^{\left(-\frac{t-t'}{\tau_{\mathrm{rec}}}\right)} dt' \tag{14}$$

whose Fourier transform is

$$I(\omega) = g \frac{eP_{\mathrm{L}}^{\mathrm{abs}}(\omega)}{h\nu} \cdot \frac{1}{1 - i\omega\tau_{\mathrm{rec}}}. \tag{15}$$

The DC part of the current is proportional to the DC component of the optical spectral power, yielding ~~for the DC current I_{DC}~~ $I_{\mathrm{DC}} \sim g$ ~~$P^{abs}_{L,av}$.~~ $P^{\mathrm{abs}}_{\mathrm{L,av}}$. The radiated power spectral density, however, becomes independent of the recombination time in the high frequency limit, $I(\omega) \rightarrow$ ~~$\frac{eP^{abs}_{L}(\omega)}{h\nu}$~~ $\frac{eP_{\mathrm{L}}^{\mathrm{abs}}(\omega)}{h\nu} \cdot$ ~~$\frac{i}{\omega\tau^{max}_{tr}}$.~~ $\frac{i}{\omega\tau_{\mathrm{tr}}^{\max}}$. This is demonstrated in the power spectrum illustrated in Fig. ~~3b~~5b for a delta-shaped optical pump pulse for the case of a long and a short-carrier-lifetime material, where both cases converge in the high frequency limit, while in the low frequency range the long lifetime material shows a much higher current. ~~A way out is also~~Thus, using short carrier transit times, ideally in the sub-ps range, might be beneficial for the pulsed case, as we will show in section 3.B. ~~Then~~In such a case, current ~~is~~ only ~~flowing~~flows for a very short time, pushing the onset of the roll-off towards high terahertz frequencies, making the device more efficient.

Besides the transit-time roll-off, all photoconductors and photomixers are prone to the RC roll-off caused by the finite device capacitance C in conjunction with the radiation resistance of the antenna ~~R_A~~ R_{A} and potentially further parallel and serial resistance caused by the semiconductor active region and the interconnects, respectively [60]. This leads to a power roll-off of ~~η_{RC}~~ $\eta_{\mathrm{RC}} =$ ~~$\frac{1}{1+(2\pi R_A C f)^2}$~~ $\frac{1}{1+(2\pi R_{\mathrm{A}} C f)^2}$ for antennas with a real-valued impedance ~~R_A~~ R_{A}. The equation can be generalized for any kind of antenna using the equivalent circuit diagram shown in ref. [~~65~~66].

Let us briefly compare the results for the three transport scenarios discussed above, namely, Eq. (8) for a CW operated short carrier lifetime photoconductor, Eq. (10) for a CW operated long carrier lifetime photoconductor and p-i-n diode, and Eq. (13) for a pulsed operated photoconductor and p-i-n diode. For short-carrier-lifetime photoconductors, both the DC and AC photocurrents are much smaller than the ideal photocurrent, while for long-carrier-lifetime photoconductors and p-i-n diodes, the low frequency and DC photocurrent amplitudes are identical to the ideal photocurrent, ~~$\bar{I}_{id}$~~ I_{id} . This has two major consequences:

1) The high photocurrent ~~I_{id}~~ I_{id} of long-carrier-lifetime photoconductors and p-i-n diodes in combination with an applied bias or built-in potential, U, generates a large amount of Joule heat. The maximum thermal load of a reverse-biased p-i-n diode or a photoconductive device before catastrophic thermal failure occurs [~~66~~67], is given by

$$P_{\max}^{\mathrm{Th}} = UI_{\mathrm{Ph}} + P_{\mathrm{L,abs}}. \tag{16}$$

where ~~I_{Ph}~~ $I_{\mathrm{Ph}} = g$ ~~I_{id}~~ I_{id} is the generated DC photocurrent and ~~$P_{L,abs}$~~ $P_{\mathrm{L,abs}}$ is the absorbed laser power. For the same heat spreader and material properties, these devices tolerate less optical pump power but generate more photocurrent from the incident pump signal. We remark that Joule heating is a less severe

boundary condition for the pulsed case as the device can relax to its dark state in between the consecutive pulses and most of the radiated power is generated in a short period of time during the optical excitation.

2) A long recombination time causes the transit-time roll-off to be limited by the carrier transit-time. In order to shift the transit-time 3 dB frequency beyond 100 GHz, comparatively short transport lengths (electrode gap sizes for photoconductors or intrinsic layer lengths for p-i-n diodes) are required, typically of the order of 150-300 nm (as opposed to gap sizes of ~2 µm normally used for short carrier lifetime CW photoconductors, e.g.). For classic electrode designs, this severely increases the capacitance of the device reducing the RC 3-dB frequency. For a p-i-n diode, for example, the capacitance is given by $C =$ ~~$\frac{\epsilon_0 \epsilon_r A}{d_i}$,~~ $\frac{\epsilon_0 \epsilon_\mathrm{r} A}{d_\mathrm{i}}$, where ~~$\epsilon_r$~~ $\epsilon_\mathrm{r} \sim 11 - 13$ for a typical semiconductor, ~~d_i~~ d_i is the transport layer length and A is the device cross section. For the p-i-n diode, the only way out is using smaller device cross sections, A, in order to counter-fight the increase of the capacitance. Unfortunately, a small device cross section limits the maximum current through the device, not only because of Joule heating, but also due to the saturation of the AC photocurrent as a result of the carrier screening effect. Therefore, transport-time and RC 3 dB frequency cannot be generally optimized independently. They are typically linked by the transport length, that should be short for transport-time optimization and long for reducing the capacitance in order to increase the RC 3 dB frequency. Still, for CW operation, p-i-n diodes excel short-carrier-lifetime photoconductors in the lower terahertz range. Due to the vast number of different devices with different parameters and designs, a strict rule on when to use a short-carrier-lifetime photoconductor and when to use a p-i-n diode for CW terahertz generation cannot be provided. In many cases, the break-even point is around 1 THz, i.e. p-i-n diodes perform better below 1 THz, while short carrier lifetime photoconductors outperform at higher frequencies. For photoconductors, however, there is an alternative for decoupling both roll-offs terms, at least partially. Reducing the transit distance by employing plasmonic absorption enhancement close to one electrode only, reduces considerably the transit-time (section 3.B), potentially to values shorter than the recombination time ~~τ_{rec}.~~ τ_rec. Since the electrode gap does not need to be reduced, the capacitance remains relatively low. In turn, the short transit-time instead of a short recombination time governs the high frequency performance as illustrated in Fig. ~~2~~4, where a long-carrier-lifetime material with short transit time and ~~a case for~~ a short-carrier-lifetime material are compared. The AC terahertz photocurrent gets closer to the ideal photocurrent ~~I_{id}~~ I_id, requiring considerably less optical pump power in order to obtain the same terahertz power as a short-carrier-lifetime (i.e. low gain) photoconductor that delivers a photocurrent amplitude of ~~I_{ph}~~ $I_\mathrm{ph} = g$ ~~I_{id}~~ I_id, at best. In turn, the lower optical pump power also relieves thermal effects given by Eq. (16).

An often-neglected consideration is photocurrent saturation in ~~photoconductor~~photoconductors and p-i-n based terahertz emitters. On the one hand, a photocurrent close to the ideal photocurrent enables high-efficiency terahertz generation at low optical pump powers. On the other hand, a large amount of charges generated in the ~~device~~device's active region at high optical pump powers screens the built-in or applied DC field [60]. At large photocurrents, the screening causes regions with negligible accelerating field, leading to the saturation or even reduction of the generated radiation (lower terahertz power despite higher optical pump power). Saturation generally sets in at photocurrent densities between 20 kA/cm² and 100 kA/cm², corresponding to photocurrents in the range of 2-20 mA for typical device geometries. Still, this permits terahertz powers in the several mW range. The situation is severely aggravated under pulsed operation, where all charges are generated within the pulse duration that is typically much shorter than the carrier transit-time and recombination time. The large charge density quickly saturates p-i-n diodes [~~63~~64], therefore, they are hardly used in pulsed operation. Long-carrier-lifetime materials are typically not used under CW operation as fields on the order of several 10 kV/cm, ideally >50 kV/cm, are required for efficient charge separation. With typical planar electrode gaps of the

order of 2 µm, this corresponds to DC biases in the range of 10 V. Combined with a photocurrent close to the ideal photocurrent, the thermal load (Joule heat in Eq. (16)) heavily limits the laser power. For pulsed operation, however, the photoconductor relaxes to the dark state between consecutive optical pump pulses. As the high frequency part of the emitted spectra of short carrier lifetime and long carrier lifetime materials are similar, long carrier lifetime materials can indeed be used for pulsed terahertz generation. However, they saturate earlier and have a more pronounced low frequency spectrum, due to the large amount of travelling charges.

For photoconductive detectorsPhotoconductive terahertz detection

Photoconductors are also excellent homodyne or heterodyne detectors. Instead of applying a DC field, the terahertz field $\vec{E}_{THz}(\vec{r},t)$ biases the metal-semiconductor-metal junction while it is (usually) illuminated with the same laser signal also used for generating the terahertz wave. Consequently, the laser signal at the receiver and the received terahertz signal are mutually coherent and phase-locked. In the small signal regime, the same calculations as for the generation case apply, except that the carrier velocity in Eq. (5) has to be replaced by $\vec{\mathrm{v}}(\vec{r},t) = \mu_{AC}\vec{E}_{THz}(\vec{r},t)$, where μ_{AC} is the AC mobility of the respective carrier. In turn, the photoconductor delivers a current of $I_D(t)\sim P_L(t) * E_{THz}(t)$, where the "*" denotes the convolution operator as in Eq. (4). In most cases, only the low frequency (i.e. the rectified) component of the detected current is read out, $I_D =< I_D(t) >_T = \mathcal{R}(f)E_{THz}(\Delta t)$, where $<>_T$ denotes the time average and Δt is the relative time difference between the terahertz signal and the laser signal. $\mathcal{R}(f)$ is the laser power and transport-kinetics-dependent current responsivity and determines how much current is generated by an incident terahertz field at frequency f. The key performance parameter of a detector is the noise-equivalent power, NEP which is defined as the input power equal to the noise floor for a detection bandwidth of 1 Hz. For a photoconductor, it is given by $NEP\sim(I_N/\mathcal{R})^2$, where I_N is the noise-equivalent current (in units $\mathrm{A}/\sqrt{\mathrm{Hz}}$). For a low NEP, both the responsivity $\mathcal{R}$ has to be maximized as well as the noise current I_N minimized. Similar to sources, an ultrafast response time – no matter whether it originates from a short recombination time or a short transit-time – is essential for two reasons: 1) improved high frequency performance and 2) low detector noise. For CW operation, a long presence of carriers within the semiconductor material will create an accumulation of charges as they are constantly generated. The currents generated by these charges will interfere causing the (transit-time or lifetime (or transit-time) roll-off. In turn, the detector responsivity drops at) and 2) improved noise performance.

For point (1), the same formulas developed in the previous subsection apply to estimate the high frequencies as f^{-2}. The large amount of charges present in the material will also severely reduce the DC resistance of the device, R_{ttt}. This makes the device very sensitive to stray fields and other perturbations. More importantly, a photoconductor is a photocurrent detector, i.e. it generates a photocurrent proportional to the incident terahertz field. The low resistance increases thermal noise severely according to $I_N = \sqrt{< I_N^2 >} = \sqrt{\frac{4k_BT}{R_{ttt}}}$. This, in turn, increases the noise equivalent power, $NEP \sim < I_N^2 > = \frac{4k_BT}{R_{ttt}}$, which can only be counter-fought at frequencies below -frequency performance in photoconductive detectors. Equivalently, beyond the lifetime (or transit-time) 3dB frequency with a higher AC current amplitude given by a higher gain. Similar to cut-off frequency, its value also rolls off as $\mathcal{R}(f)\sim f^{-2}$. Like in the case of emitters, devicesdetectors engineered for a sub-ps carrier transit -time may outperform existing concepts based on short carrier recombination times, as they feature a high transit-time 3-dB cut-off frequency andwhile yielding a high rectified photocurrent, however, only under gain.

For point (2) the ~~premises that~~situation becomes a bit more intricate. One the ~~RC roll-off does not degrade due to the~~one hand, a high gain (e.g. by a very short carrier transit-time ~~optimization. Similar considerations hold for the pulsed operation, again relaxed by the criterion that the devices usually return to the dark resistance between pulses, as typical transit-times or recombination times are several orders of~~ and a long carrier lifetime) ensures a high responsivity. On the other hand, it reduces the illuminated resistance R_{ill} of the device as carriers are efficiently removed from the semiconductor material. The latter has detrimental effects on the thermal noise current which is the lower limit of the noise current I_{N}. The magnitude of the thermal noise current is given by $I_{\mathrm{N}}^{th} = \sqrt{< \left(I_{\mathrm{N}}^{th}\right)^2 >} = \sqrt{\frac{4k_{\mathrm{B}}T}{R_{\mathrm{ill}}}}$, which sets the lower limit for the noise equivalent power [68],

$$NEP \sim < \left(\frac{I_{\mathrm{N}}^{th}}{\mathcal{R}}\right)^2 > = \frac{4k_{\mathrm{B}}T}{\mathcal{R}^2 R_{\mathrm{ill}}}. \tag{17}$$

~~smaller than the time between two consecutive~~In the low laser power limit, the responsivity scales as $\mathcal{R}(f) \sim g\eta_{tr}(f)$ and the illuminated resistance is $R_{\mathrm{ill}} \sim g^{-1}$, i.e. a higher gain helps to improve the $NEP \sim g^{-1}$ in the low frequency limit. For conventional photoconductor layouts, however, a high gain engineered by long carrier lifetimes causes a low transport-time 3 dB frequency compromising the high frequency performance as discussed in point (1). This usually leads to drastically worse performance in the terahertz range. For this reason, conventional photoconductive detectors use short carrier lifetime material. A way out is presented in section 3.B using plasmonic structures with very short transit-time.

A further problem arises for high laser power levels, where the responsivity may substantially deviate from the linear dependence on the photoconductive gain. Further, high currents increase the semiconductor temperature which further reduces R_{ill}. For short transit-time detectors that feature a high photoconductive gain, and hence a much lower value of R_{ill}, the effect is conveniently compensated by the use of lower optical ~~pump pulses. This~~powers, which in the end results in ~~a higher average illuminated resistance and less current noise.~~similar, or superior, signal-to-noise ratios.

An open issue that still requires further investigation is the influence that the laser noise plays in the noise of both short-carrier lifetime and short transit-time photoconductive detectors for CW and pulsed operation.

3. Terahertz photomixers and photoconductors without short-carrier-lifetime semiconductors

3.A Photomixers based on p-i-n diode junctions

P-i-n diodes are mostly used for CW terahertz generation as they produce a photocurrent close to the ideal photocurrent (i.e. unity gain) while still offering transit-time roll-off 3dB frequencies > 100 GHz. P-i-n diodes operating in the terahertz range require dedicated high frequency optimization. In section 2 we have shown that the transit-time roll-off 3 dB frequency is determined as $f_{tr}^{3dB} \approx \frac{1}{2\tau_{tr}} f_{\mathrm{tr}}^{3\mathrm{dB}} \approx \frac{1}{2\tau_{\mathrm{tr}}} = \frac{\bar{\mathrm{v}}}{d_i}\frac{\bar{\mathrm{v}}}{d_{\mathrm{i}}}$, where ~~$d_i$~~$d_{\mathrm{i}}$ is the length of the transport section, mostly the intrinsic layer of the device. The high effective mass of holes and the absence of the velocity overshoot causes holes to be considerably slower than electrons, at least in direct bandgap semiconductors. Therefore, typical terahertz p-i-n diodes are designed to absorb the optical power somewhere close to or even within the p-contact layer such that charge separation is dominated by the electron transport. The p-i-n diodes are usually small, lumped

element devices with sizes between ~2 and 100 µm² attached to an antenna. A variety of p-i-n diode concepts has evolved in the meantime. The concepts discussed in the following focus on telecom-wavelength (i.e. ~1550 nm) compatible devices although the discussed concepts can (and to a certain degree have been) also be transferred to 800 nm operated GaAs-based devices.

Operation of uni-travelling carrier photodiodes

The widest spread version is the uni-travelling carrier (UTC-PD) photodiode or its variants, modified UTC-PD (MUTC-PD) and other trade names [45, 46, ~~67~~ 69-71]. The dark and illuminated UTC band structure of a typical 1550 nm design is illustrated in Fig. ~~4a~~6. The structure begins with a high band gap p-type material such as p-InP or p-InAlGaAs, followed by a p-type $In_{0.53}Ga_{0.47}As$ absorber layer. As both layers are p-type, the Fermi level is pinned, aligning the valence band edges. This way, the high band gap layer acts as a diffusion block layer for electrons but permits holes to travel to the p-contact shown on its left side. The absorption of the ~1550 nm optical pump signal takes place in the low band gap InGaAs layer. The next part is a weakly n-doped or intrinsic sequence of InGaAs via quarternary InGaAsP to InP, often termed as cliff layer. Although a continuously graded transition from InGaAs to InP would cause less scattering and more homogeneous electric field distribution, it is fairly difficult to grade InGaAs via quarternary InGaAsP to InP while ~~remaining~~maintaining lattice-matching to InP and thus high crystalline quality. The main obstacle is stabilizing the group V elements during growth. The transition is followed by a weakly n-type doped or intrinsic InP transport layer and finally the n-InP contact.

The UTC structure features the following advantages: The absorption within the p-contact ensures that the charge separation and carrier transport is governed by the electrons that have to travel across the intrinsic layer. Electrons strongly benefit from ballistic effects that holes do not show, particularly in InP with an energy difference between the Γ and L-valley (being the next higher side valley) as large as 0.59 eV [~~70~~72]. Further, the InP transport layer features a comparatively high thermal conductivity of 0.68W/(~~cmK~~cm·K), approximately 12 times higher than that of InGaAs, enabling high optical power densities before thermal destruction of the device occurs. Usually, the transport saturates before the device breaks down thermally. Saturation originates from the fact that the separation of optical charges generates an electric field that opposes the built-in field [60]. To a certain degree, this field can be compensated with external bias (~~see~~ Fig. ~~4a~~6), however, charges within the transport layer alter the field locally which then bends the transport layer upwards. The strength of this effect depends on the optical power. At highest optical power levels, the field distribution within the intrinsic layer becomes so severely distorted, that the region close to the p-contact does not show a noticeable acceleration field anymore (current blocking) while the field at the n-contact side is so high that severe side valley scattering sets in, reducing the electron speed to the comparatively slow saturation velocity at best. To a certain degree this effect can be mitigated by slightly n-doping the transport layer, thus causing a bend in the opposite direction. This has enabled peak current densities of the order of 70-100 kA/cm² [~~69,~~ 71~~-~~, 73-75], corresponding to photocurrents on the order of 10-20 mA for devices operating around 1 THz. At lower frequencies, larger devices may be used, thus also permitting higher currents.

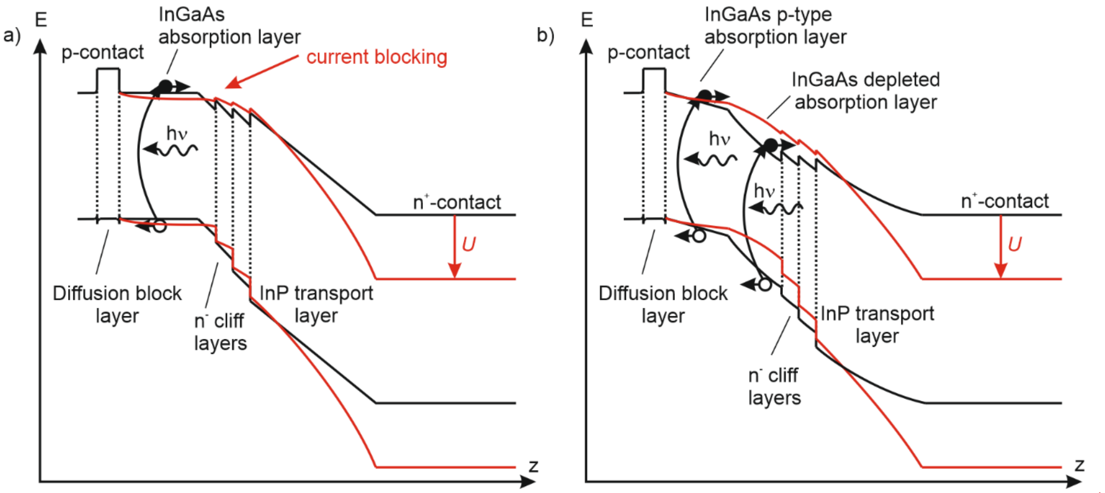

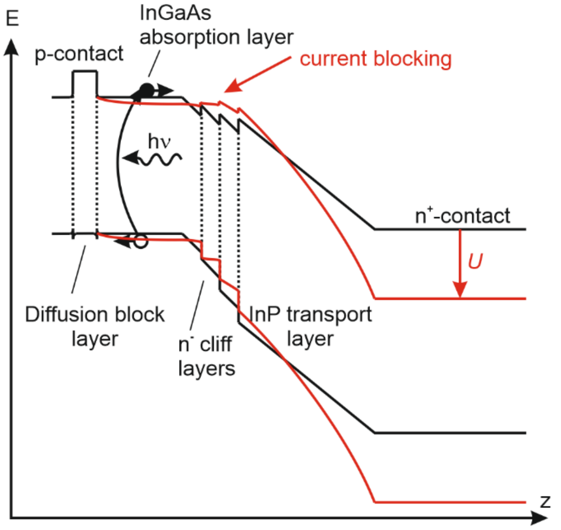

Figure ~~4~~6: Band structure of an UTC ~~(a) and an example of a MUTC~~ diode ~~(b)~~ in the dark (unbiased, black) and illuminated conditions (reverse-biased, red). The vertical axis represents the energy level *E*, while the horizontal axis the spatial z-coordinate. *U* represents the applied bias. ~~The MUTC features an n-type background in the transport layer in order to counterfight charge screening effects. The doping level and band bending under dark conditions are exaggerated for better visibility.~~ The UTC diode suffers from current blocking at high optical power levels. The effect is less severe if the transport layer ~~and depleted absorber layer are~~is slightly n-type.

The main downside of UTC diodes is their flat, almost field-free absorber layer. Photoexcited electrons generated in this layer first have to diffuse out towards the transport layer where they are then accelerated by the built-in field. The drift process is fairly slow, causing a severe increase of the transit-time and therefore a reduction of the 3-dB frequency [~~68~~70]. Different variants of MUTC diodes aim for mitigating this issue. One option is to split the absorber layer between a flat low-p-doped part and an intrinsic or slightly n-doped (but depleted) InGaAs section that is part of the transport layer ~~as illustrated in Fig. 4b~~. The AC current is then composed of three constituents: (i) The diffusion current of electrons that are absorbed in the (shorter) flat section of the absorber. These electrons are comparatively slow. (ii) the electron current that is generated within the absorber section in the transport layer. Electrons are accelerated by the local field right away to ballistic velocities resulting in a very short transit-time. (iii) the hole current generated within the absorber of the transport layer. Though holes feature about an order of magnitude higher effective mass in InGaAs and do not show any velocity overshoot, the ~~length of the~~ absorber layer length in the transport region is usually chosen short enough to allow for holes reaching

the p-contact within a comparatively short time. Theoretical combined transport 3 dB frequencies lie in the range of 600 GHz [~~68]. ~~70].

Further design optimizations include a graded doping profile in the flat absorber layer. By decreasing the p-type doping towards the transport layer, moving the Fermi energy away from the valence band, a quasi-field is formed that accelerates electrons towards the transport layer. A further positive effect arises when the device is operated close to saturation requiring an external reverse bias to restore the transport field. Part of the bias drops also at the weakly doped flat absorber, further increasing the accelerating field towards the transport layer. The highest so far reported output power levels with UTC or MUTC diodes are 10-16 mW at 100 GHz (resonant) [~~74, 75~~76, 77] and about ~~10µW~~10 µW at 1 THz (broadband) [~~76~~78]. A second issue with standard UTC diodes is their comparatively low optical absorption coefficient. The transit-time optimization dictates a short absorber region of the order of 50-150 nm, depending on the design operation frequency. For InGaAs around 1550 nm, the absorption coefficient is of the order of 6000-8000 cm^{-1} [~~77~~79], somewhat depending on the doping level due to band gap shrinkage at high doping levels. For vertical illumination (i.e. along the semiconductor growth direction), a 100 nm absorber only captures 6-8% of the incident light, corresponding to a responsivity of only 0.048 A/W- 0.064 A/W.

The way out is integration of the photodiode with a passive optical waveguide (POW). The POW is typically within the bottom contact layer of the diode composed of a quarternary InGaAsP layer that serves as waveguide core and InP as waveguide cladding towards the substrate side and an air as cladding towards the air side [~~75, 78, 79~~77, 80, 81] as illustrated in Fig. ~~5~~7. Like in an optical fiber, the 1550 nm wave is guided along the waveguide that is fed by a (sometimes lensed) fiber at the chip's edge. When the light arrives at the terahertz diode that is mounted on top of the waveguide, the high refractive index of the diode (the refractive indices of InGaAs, InP and respective quarternaries are all of the order of 3.1-3.7) causes the guided wave to leak upwards into the diode. The light is thus absorbed along the diode, perpendicular to the carrier transport direction. In turn, the diode length, not just the absorber thickness, determines the overall absorption. Device lengths of the order of 10 µm suffice to absorb more than 50% of the optical power. Narrow but long diodes allow to keep the device cross section area at a reasonable level, mitigating the RC roll-off. The main limitation on the waveguide length ~~to the active device ~~is free carrier absorption as the waveguide layers usually are also used as bottom contact layers. Although the bandgap of the used POW materials is too large for inter-band absorption, the required doping to form a low-resistance bottom contact causes free carrier absorption, imposing limits on either the waveguide length or the maximum doping. As free carrier absorption is more severe for p-type III-V semiconductors, the bottom contact is usually chosen to be an n-contact with a moderate doping. Further, the POWs are kept as short as possible.

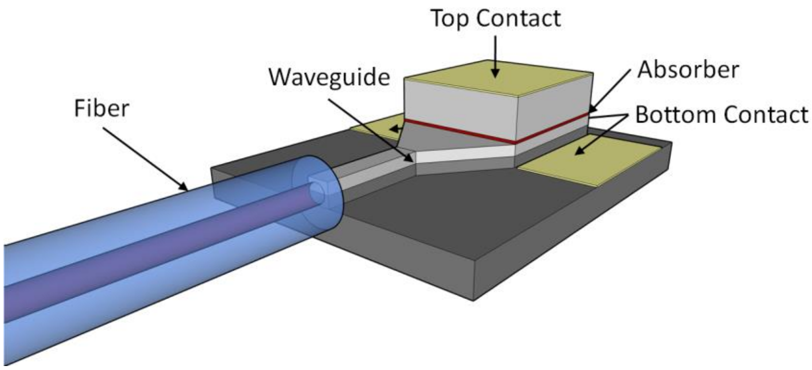

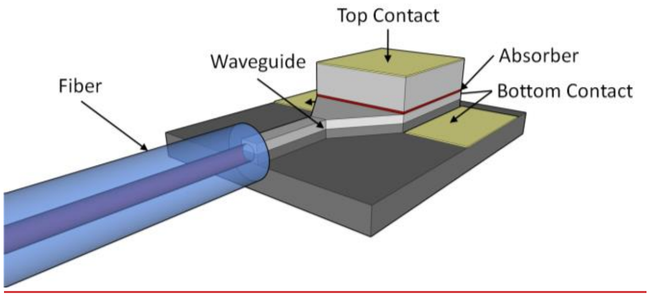

Figure ~~5~~7: POW-integrated terahertz diode. The fiber is not to scale.

Examples of POW-integrated diodes are classical UTC/MUTC designs with a reported 1550 nm responsivity up to 0.53 A/W delivering about 5 µW at 1 THz [~~72~~74], and triple transit region diodes with 0.5 A/W and a terahertz power of ~1 mW at 0.11 THz at 16 mA photocurrent [~~78~~80], while the theoretical maximum 1550 nm responsivity is 1.25 A/W.

Another alternative, though technologically much more challenging, is fabricating the UTC-PD in an asymmetric resonant Fabry-Pérot cavity [~~80~~82]. The sample is illuminated along the surface normal. The top contact is semi-transparent, with a defined reflection coefficient. Via substrate transfer, the diode is lifted off its host substrate and mounted on a gold-coated host. The gold layer acts as bottom reflector, causing the light to travel several times through the absorber. If the correct device height is chosen and the top contact reflection and the loss of power via absorption within a round trip are balanced to cause perfect destructive interference for any outcoupled light, theoretically all optical power can be absorbed within the device. The authors of ref. [~~80~~82] achieved a peak optical responsivity of 0.29A/W under DC operation with low optical power levels at the optimum wavelength. Under terahertz operation, the responsivity dropped to 0.12 A/W offering a terahertz power of 0.75 mW at 300 GHz with a photocurrent of 9.8 mA (optical power of 83 mW).

Other diode-based terahertz sources

Besides UTC and UTC-like designs, also double heterostructure diodes are implemented at terahertz frequencies. As there are also hole currents in the intrinsic layer, they saturate earlier [~~76~~78]. However, a considerably short intrinsic layer still enables efficient operation at terahertz frequencies. Integrated with a POW these "waveguide integrated diodes" offer an optical responsivity of 0.27 A/W. They offered a terahertz power of 4 µW at 1 THz, just about 3 dB less than a direct comparison to a state-of-the-art UTC diode [~~76~~78]. Both devices, compared in ref. [~~76~~78], are commercially available. The other extreme are fully ballistic diodes, where absorption takes place only in a graded intrinsic layer without any flat absorber and any cliff layer. The band gap is linearly increased by adding up a few percent Aluminum to InGaAs towards the n-contact [~~81~~83]. This way, the absorption region is also confined close to the p-contact with a minor part of the current due to holes that have to travel on the order of 40 nm only, while electrons travel ballistically ~160 nm. Cascading such diodes leads to the n-i-pn-i-p superlattice concept [~~82~~84]. In between the cascaded diodes, efficient ErAs-enhanced recombination diodes are implemented in order to reduce the bias drop at the np junctions emerging between two subsequent periods. While transit-time-3dB frequencies around 0.85 THz were reported [~~63~~64], the comparatively poor thermal conductivity of the required InGaAs to InAlGaAs grading limits the maximum photocurrent density to about 20 kA/cm². The highest output power achieved at 1 THz so far is around 0.8 µW ~~(-31 dBm)~~ with a broadband design [~~61~~62]. For further details on 1550 nm operated III-V UTC diodes and terahertz diodes the reader is referred to ref. [~~83~~85].

Although the optical~~-~~ and transport properties of indirect bandgap semiconductors are not favorable for terahertz generation, silicon-integrated germanium [~~84~~86] and SiGe [~~85~~87] diodes are technologically very interesting as they may become comparatively inexpensive and can be directly integrated with high frequency SiGe electronic circuits. So far, silicon-integrated germanium diodes have demonstrated operation up to 1.3 THz with a responsivity of 1.2 A/W and a power of 25 µW at 100 GHz with only 4 mW of 1550 nm laser power [~~84~~86]. Another example of a SiGe diode has achieved 16 µW at 200 GHz [~~85~~87].

In summary, p-i-n diodes

- Feature a close-to-ideal laser power to photocurrent conversion (gain ~1).
- Frequently make use of ballistic charge transport.
- Offer high terahertz power at considerably low laser power, at least below 1 THz.
- Offer excellent performance under CW operation but saturate under pulsed operation.
- Feature an overall roll-off 3 dB frequency below 0.5 THz.
- Can (mostly) be manufactured by standard growth techniques.
- Perform very poorly as terahertz detectors due to their built-in field.

3.B Photoconductors and photomixers based on plasmonic nanostructures

As explained in the previous sections, the use of long carrier lifetime photoconductors for CW terahertz generation is generally limited due to severe Joule heating. However, long carrier lifetime photoconductors have been successfully used for realizing pulsed terahertz emitters [~~86-89~~88-91], since the photoconductor can relax to the dark state between consecutive optical pump pulses. In addition, the use of long carrier lifetime photoconductors for terahertz detection in both pulsed and CW operation modes is generally limited by the relatively long duration of the photoconductor's impulse response.

It was recently shown that the use of plasmonic nanostructures can reduce the impulse response duration of long carrier lifetime photoconductors, enabling the realization of CW terahertz emitters and pulsed/CW terahertz detectors through these photoconductors [~~90-95~~92-97]. This is because the concentration of the optical pump beam and photo-generated carriers in the photoconductor active area can be manipulated by the use of plasmonic nanostructures so that the transport path length of the majority of the photocarriers is significantly reduced.

Plasmonic nanostructures are usually designed as periodic metallic patterns that, under momentum matching conditions, enable the excitation of surface plasmon waves at the metal-dielectric interface by an incident transverse magnetic optical beam. The existence of surface plasmon waves leads to strongly confined optical near field, which has already enabled the development of numerous high performance terahertz emitters and detectors [47-54, ~~93-101~~95-103], among other advancements in nonlinear optics [~~102~~104], near field imaging and spectroscopy [~~103, 104~~105, 106], as well as electromagnetic waves detection and manipulation [~~105-~~107-109].

Photoconductive terahertz generation and detection enhanced by plasmonic nanocavities

Plasmonic enhancement of the optical pump absorption in a thin photoconductor layer (<200 nm thickness) through the utilization of plasmonic nanocavities is a promising method to provide an ultrafast response for the photo-generated carriers without requiring a short-carrier-lifetime substrate. To achieve a sub-picosecond transit time for all of the photo-generated carriers, the plasmonic nanocavities should

be designed to confine the optical absorption in small volumes near the terahertz radiating elements. This results in a significant reduction of the average carrier transport path length and consequently all photo-generated electrons can drift to antenna electrodes within a sub-picosecond time scale. By increasing the quantum efficiency and ultrafast operation simultaneously, terahertz detectors and emitters based on nanocavities can offer significantly enhanced sensitivity levels and optical-to-terahertz conversion efficiencies over a large terahertz operation bandwidth.

In ref. [48] a high-sensitivity terahertz detector without using a short-carrier-lifetime substrate was developed, based on an optical nanocavity with a resonance wavelength of ~770 nm wavelength consisting of an array of plasmonic gold nanoantennas and a distributed Bragg reflector (DBR). A 190-nm-thick GaAs layer was used as the photoconductive layer sandwiched between the plasmonic gold nanoantennas and the DBR. 25 alternating pairs of AlAs and $Al_{0.33}Ga_{0.67}As$ layers were used to form the DBR~~ (Fig. 6a).~~. The plasmonic gold nanoantennas featured a 200 nm periodicity, a 100 nm gap, and~~ a~~ 80 nm height, with a 350 nm Si_3N_4 antireflection coating such that the structure exhibited an optical absorption of 80% within the thin GaAs layer at 770 nm. Characterization of the detector was performed in a terahertz time-domain spectroscopy setup, where terahertz pulses were generated from another photoconductive emitter excited by a femtosecond Ti-sapphire laser with a pulse width of 130 fs. ~~As shown in Fig. 6b, ~~This nanocavity-based detector prototype is capable of detecting terahertz pulses over a 0.1–4.5 THz frequency band with more than a 100 dB peak dynamic range under only a 5 mW optical power, exhibiting a comparable performance with the state-of-the-art photoconductive terahertz detectors based on short-carrier-lifetime LT-GaAs substrates.

In a follow up work, the ultrafast performance of terahertz detectors based on plasmonic nanocavities were further improved by designing a nanocavity that provides more than 50% optical absorption within only 60 nm depth inside the photoconductive layer below the plasmonic nanoantennas (Fig. ~~6c~~8a) [49]. In addition to the modified optical absorption profile, to further reduce the carrier transit time, the modified detector design incorporated a 100-nm-thick $In_{0.05}Ga_{0.95}As$ as the photoconductive layer instead of traditionally used GaAs, to obtain a higher carrier mobility and a larger optical absorption coefficient. By doing so, the terahertz detector offered very high sensitivity levels even at sub-mW optical pump power levels. With a terahertz emitter based on large-area plasmonic nanoantenna arrays [~~101~~103], a 100 dB peak dynamic range over a 0.1-6 THz usable frequency band was demonstrated through this plasmonic-nanocavity-based detector at average optical pump power levels as low as 0.1 mW~~ (~~, as shown in Fig. ~~6d).~~8b.

A similar plasmonic nanocavity enhancement mechanism was also used to implement high-power, high-efficiency terahertz emitters [50]. Like ref. ~~1,~~[48], a 190-nm-thick GaAs photoconductive layer and a DBR consisting of 25 alternating pairs of AlAs and $Al_{0.33}Ga_{0.67}As$ were used to form the plasmonic nanocavity (Fig. ~~6e~~8c). The plasmonic nanoantennas deposited on the photoconductive layer were specifically designed to maximize the optical absorption in the GaAs layer at ~ 770 nm wavelength, leading to an optimized radiation efficiency at this wavelength. As a result, the nanocavity-based terahertz emitter provided a 4 mW average pulsed terahertz radiation power over 0.1-5 THz, demonstrating more than 20 times higher optical-to-terahertz conversion efficiency compared to a similar plasmonic nanoantenna terahertz emitter fabricated on LT-GaAs substrate (Fig. ~~6f~~8d).

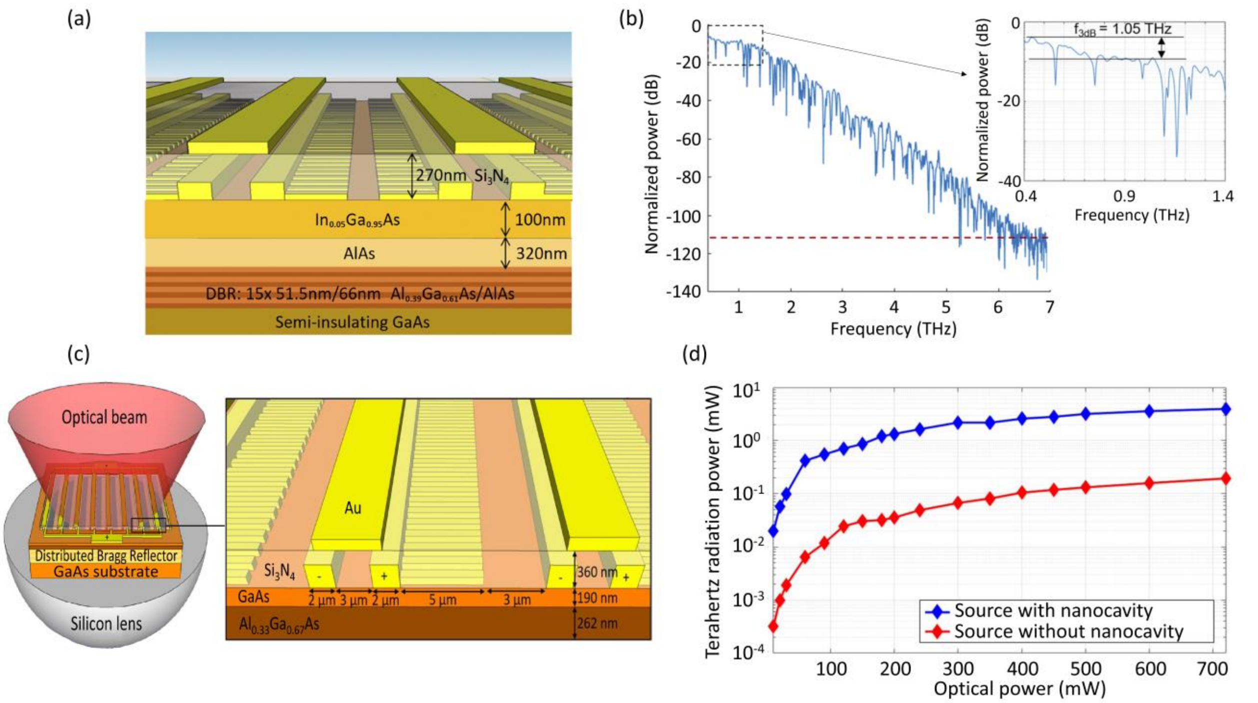

Figure 8: (a) A modified terahertz detector based on plasmonic nanocavities, which is designed to maximize the photo-generated carrier concentration within a 60 nm depth below the plasmonic nanoantennas. (b) The frequency-domain power spectrum obtained with this detector at 0.1 mW optical pump power level. Adapted from [49]. (c) A terahertz emitter based on plasmonic nanocavities and (d) its radiation power compared with a plasmonic nanoantenna array based on a short-carrier-lifetime LT-GaAs photoconductor as a function of the optical pump power. Adapted from [50].

The use of plasmonic electrodes fabricated on a thin photoconductive layer for confined optical generation can be easily scaled to other pump wavelengths to realize high-performance terahertz devices without short carrier lifetime semiconductors. To demonstrate the wide applicability of the concept, terahertz detector prototypes optimized for operation at 1040 nm and 1550 nm optical pump wavelengths were implemented [93, 9495, 96]. These are two important optical bands where fiber-optic technologies are commercially available with high reliability, low cost, and compact footprints. As shown in Figs. 7a9a and 7c9c, both detector prototypes are fabricated on an InGaAs photo-absorbing layer no more than 200 nm thick, with Indium concentrations of 24% and 53% for the 1040 nm and 1550 nm detectors, respectively, to adjust the bandgap energy for achieving high optical absorption. In both cases, a 200 nm AlAs buffer layer is first grown on a SI-GaAs substrate in order to reduce the dark conductivity and the noise level of the detectors, followed by the growth of the InGaAs layer. Due to the higher conductivity of the $In_{0.53}Ga_{0.47}As$ layer as a result of its smaller bandgap energy, the 1550 nm detector utilized a spiral-antenna-based design with a small active area (1 µm × 10 µm), as opposed to the nanoantenna array design of the 1040 nm detector. The plasmonic electrode geometries for both detectors (280 nm periodicity, 80 nm gap, and 3/77 nm Ti/Au thickness, with 380 nm Si_3N_4 for the 1040 nm detector, and 460 nm periodicity, 80 nm gap, and 3/77 nm Ti/Au thickness, with 240 nm Si_3N_4 for the 1550 nm detector) are optimized according to their respective optical pump wavelengths to maximize the optical absorption within the thin InGaAs layer, leading to greatly reduced carrier transit time. Detection bandwidths of 4 THz and 3.6 THz are demonstrated with the detector prototypes operating at 1040 nm and 1550 nm, respectively (Figs. 7b9b and 7d9d). These demonstrations validate the broad applicability of carrier transit time reduction through the use of plasmonic nanostructures on a thin photo-absorbing

layer and, hence, they open up new possibilities of designing and realizing high-performance terahertz devices without being limited by the availability of short-carrier-lifetime semiconductors.

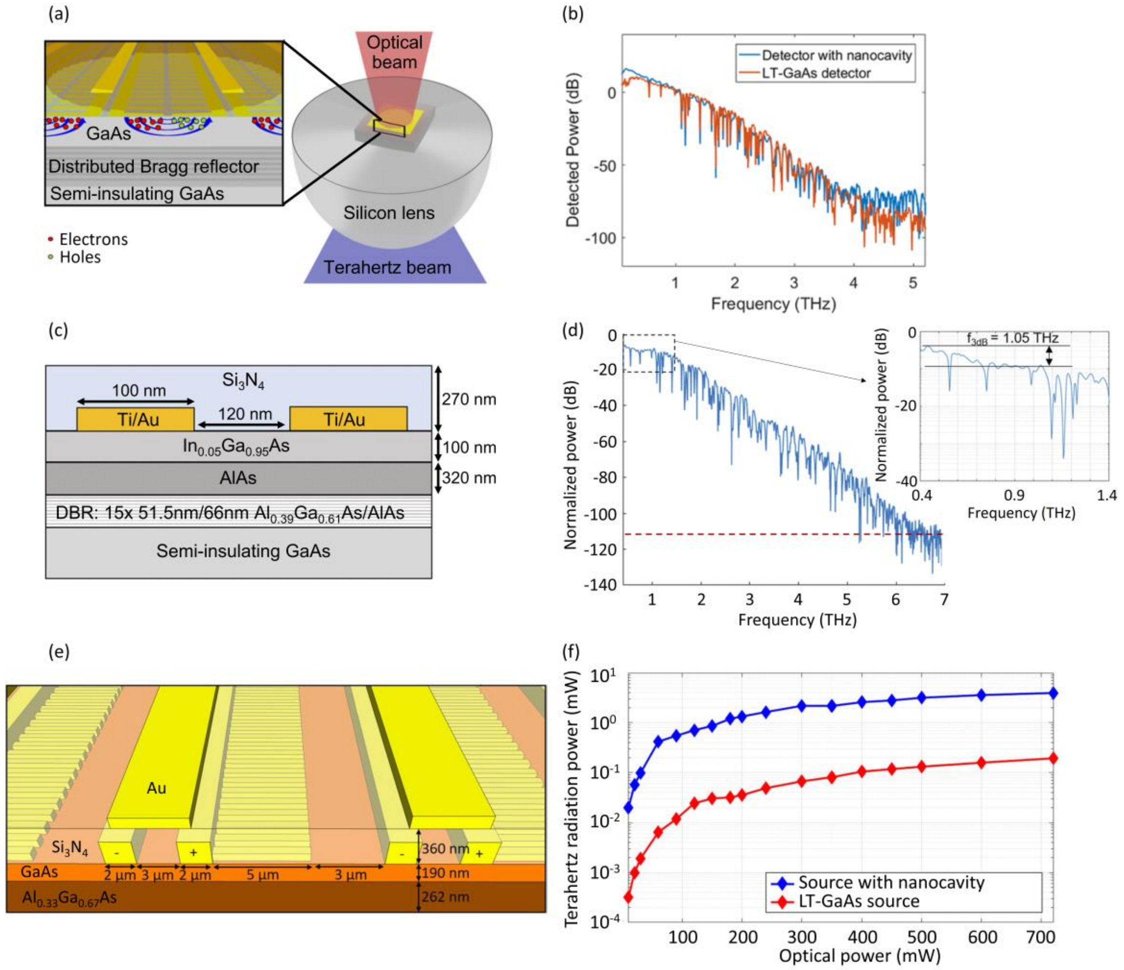

Figure 6. (a) A first-generation terahertz detector based on plasmonic nanocavities and (b) a comparison of its detected power spectrum with a state-of-the-art LT-GaAs detector. (c) A modified terahertz detector based on plasmonic nanocavities, which is designed to maximize the photo-generated carrier concentration within a 60 nm depth below the plasmonic nanoantennas. (d) The frequency-domain power spectrum obtained with this detector at a 0.1 mW optical pump power level. (e) A terahertz emitter based on plasmonic nanocavities and (f) its radiation power compared with a plasmonic nanoantenna array based on a short-carrier-lifetime LT-GaAs photoconductor as a function of the optical pump power.

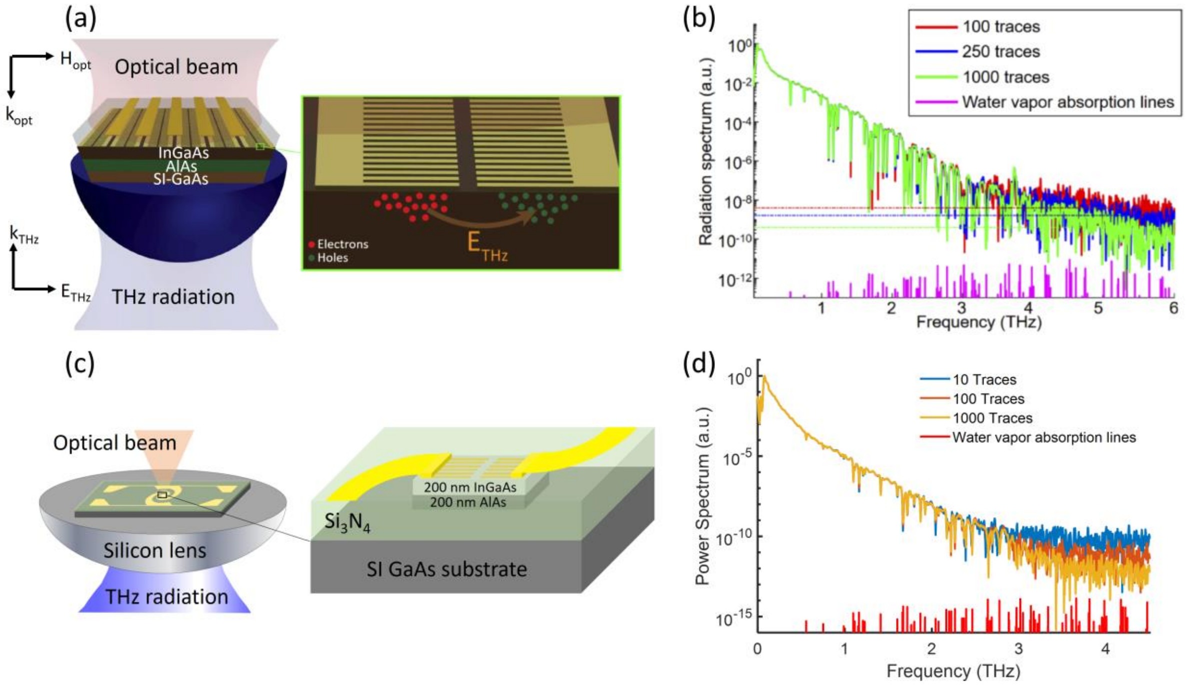

~~Figure 7~~

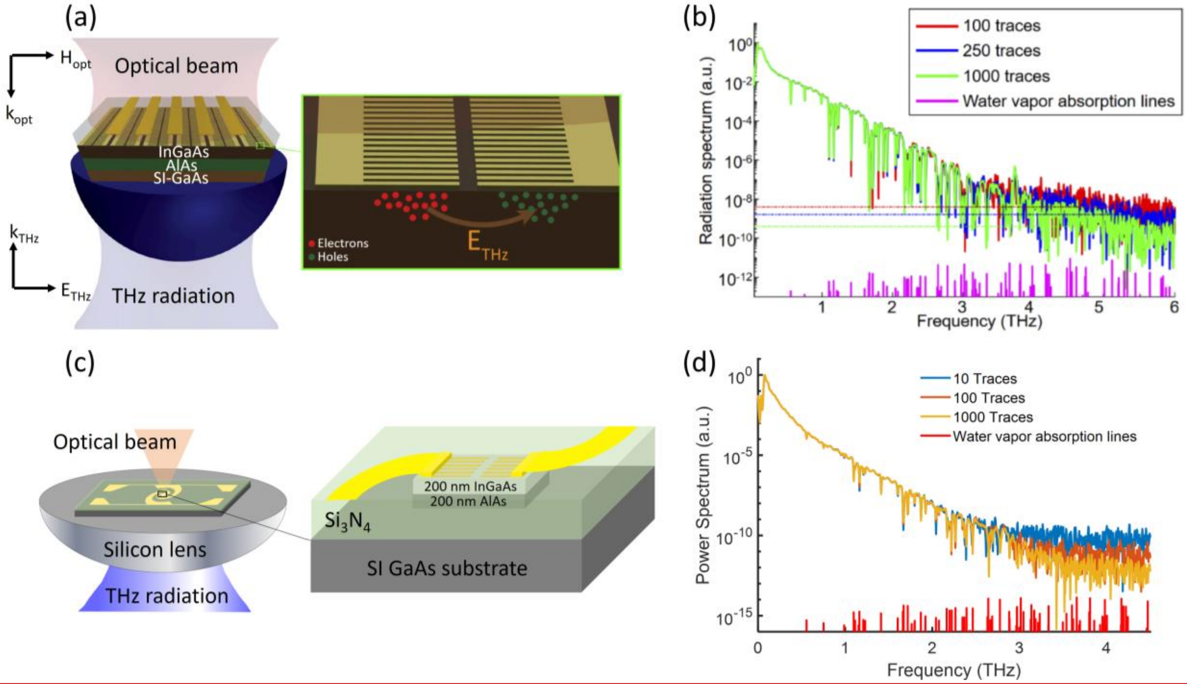

Figure 9: (a) Schematic diagram and the (b) detected power spectra under different number of averaged traces for the 1040 nm detector at an incident average terahertz power of 6.8 μW. Reprinted by permission from [95]. Copyright (2020) by The Optical Society. (c) Schematic diagram and the (d) detected power spectra under different number of averaged traces for the 1550 nm detector at an incident average terahertz power of 206 μW. Reprinted by permission from [96]. Copyright (2020) by The Optical Society.

Plasmonics-enhanced bias-free terahertz sources

The use of plasmonic nanostructures has been also very effective for enhancing the terahertz radiation power from bias-free terahertz emitters that utilize the naturally-induced built-in electric field

on the semiconductor surface. When the semiconductor lattice is terminated, the break in the lattice symmetry results in the formation of electronic states with energies lying between the conduction band and the valance band of the semiconductor. These states can act either as donors or acceptors which carry a positive or negative charge when ionized, respectively. The ionized states at the semiconductor surface push the Fermi energy to the charge neutrality level of the surface states, i.e. to the position where the net charge of the states is zero. This Fermi level pinning results in a band bending between the bulk and the surface of a semiconductor material as illustrated in Fig. ~~8a [108-111~~10a [110-113]. As a result, a built-in electric field is formed between the semiconductor surface and the bulk. The electric field present at the semiconductor surface can be used for ultrafast transit of the photocarriers and generation of terahertz radiation. When the pump photons are absorbed in the semiconductor, the generated electron-hole pairs close to the semiconductor surface can be accelerated by this built-in field and generate an ultrafast current within the semiconductor, realizing photomixing within the substrate.

In a conventional surface-field emitter, optical excitation is directed under an angle with respect to the semiconductor surface normal such that terahertz waves normal to the semiconductor surface can be generated to allow more effective collection and utilization of the radiation. Terahertz generation as a result of the transported carriers through the surface built-in field (~~illustrated in~~ Fig. ~~8a~~10a) should not be confused with the photo-Dember effect (~~illustrated in~~ Fig. ~~8b~~10b). When an optical pump beam is absorbed in the semiconductor, photogenerated electrons and holes diffuse from the surface, where most carriers are generated according to Lambert Beer's law, towards the bulk due to the formation of a concentration gradient. The higher effective mass (lower mobility) of holes causes a difference between the hole and electron diffusion currents, resulting in the formation of a dipole inside the substrate, giving rise to terahertz generation (Fig. ~~8b~~10b). This process is referred to photo-Dember effect. The polarity of the generated terahertz electric field can be used to determine whether carrier drift or diffusion dominates the terahertz generation process. In most n-type semiconductors, the built-in field direction is towards the surface, drifting the photogenerated electrons towards the bulk, and holes towards the surface. In most p-type semiconductors, the built-in field direction is towards the semiconductor, drifting the electrons towards the surface and holes towards the substrate. Therefore, the polarity of this drift current flips when the semiconductor doping type is changed, resulting in a polarity change in the generated terahertz electric field. In contrast, the net direction of electron diffusion is always from the semiconductor surface towards the substrate. Therefore, the direction of the current is independent of the doping type of the semiconductor.

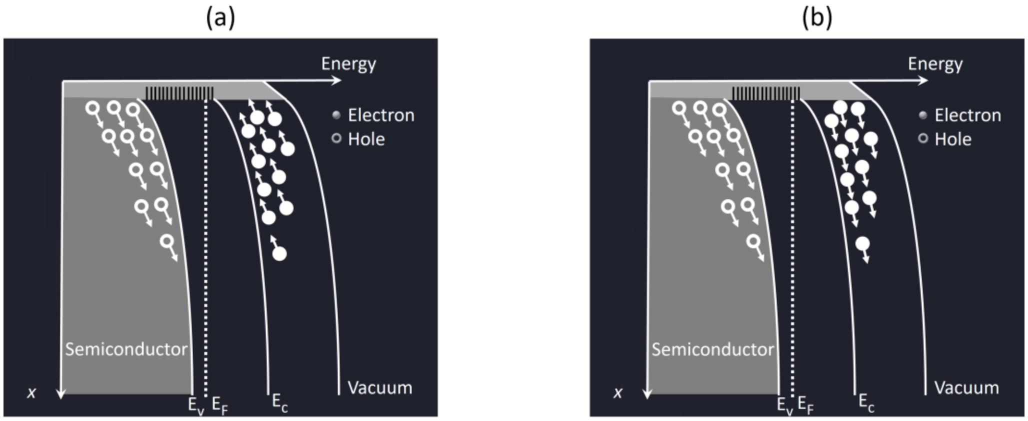

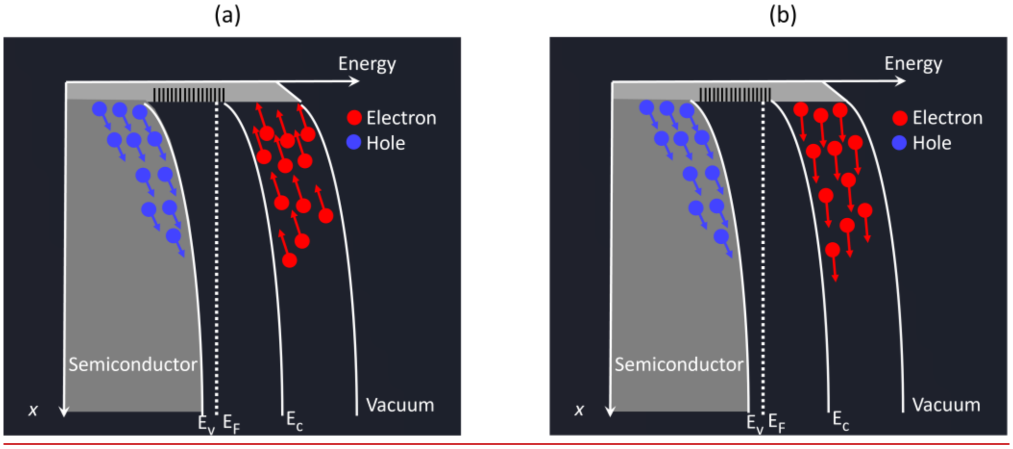

Figure ~~8~~10: The band structure of a p-type semiconductor surface emitter utilizing (a) the built-in electric field induced near the semiconductor surface and (b) photo-Dember effect.

Semiconductor surface states may induce very high built-in electric fields thanks to the Fermi level pinning at the surface. However, the extent of the built-in electric field is shallow in many semiconductors, limiting the amplitude of the generated ultrafast photocurrent for terahertz generation [~~112~~114]. It was recently shown that plasmonic nanoantennas can be used to efficiently couple the incident optical pump beam to the semiconductor surface states, enabling a high concentration of photocarrier generation near the semiconductor surface where the intensity of the built-electric field is maximized, leading to high-efficiency terahertz generation.

Figure ~~9a~~11a shows the schematic diagram and operation principles of a bias-free terahertz emitter design based on the plasmon-coupled surface states concept. An epitaxially grown 100 nm/500 nm undoped/p~~-~~$^{+}$-doped (p = 10^{19} cm^{-3}) InAs photoconductive layer grown on a SI-GaAs substrate is used to optimize the built-in electric field extent and strength near the surface [~~95~~97]. The nanoantenna geometry of 440 nm periodicity, 80 nm gap, 100 nm height, and covered with 240 nm Si_3N_4 anti-reflection coating is chosen to excite surface plasmon waves at a 1550 nm optical pump wavelength while maximizing the spatial overlap between the optical generation profile and the built-in electric field distribution. Confinement of optical generation mostly within the undoped InAs layer allows the photocarriers to travel with strong acceleration and high mobility. Additionally, the nanoantenna length of 2 µm is designed to maximize the induced ultrafast current density on the plasmonic nanoantenna array, which maximizes the generated terahertz radiation. Fig. ~~9b~~11b shows the fabricated fiber-coupled passive terahertz emitter prototype based on the described design, which generates terahertz radiation with a 4 THz bandwidth and a 105 dB peak dynamic range in conjunction with an ErAs:InGaAs-based photoconductive detector, under excitation by 150 fs optical pulses (Fig. ~~9c) [95~~11c) [97]. The achieved optical-to-terahertz conversion efficiencies by this emitter exceed previously demonstrated bias-free optically pumped terahertz emitters based on non-linear optical processes, spintronics, and photo-Dember effect, as detailed in Fig. ~~9d.~~ 11d.

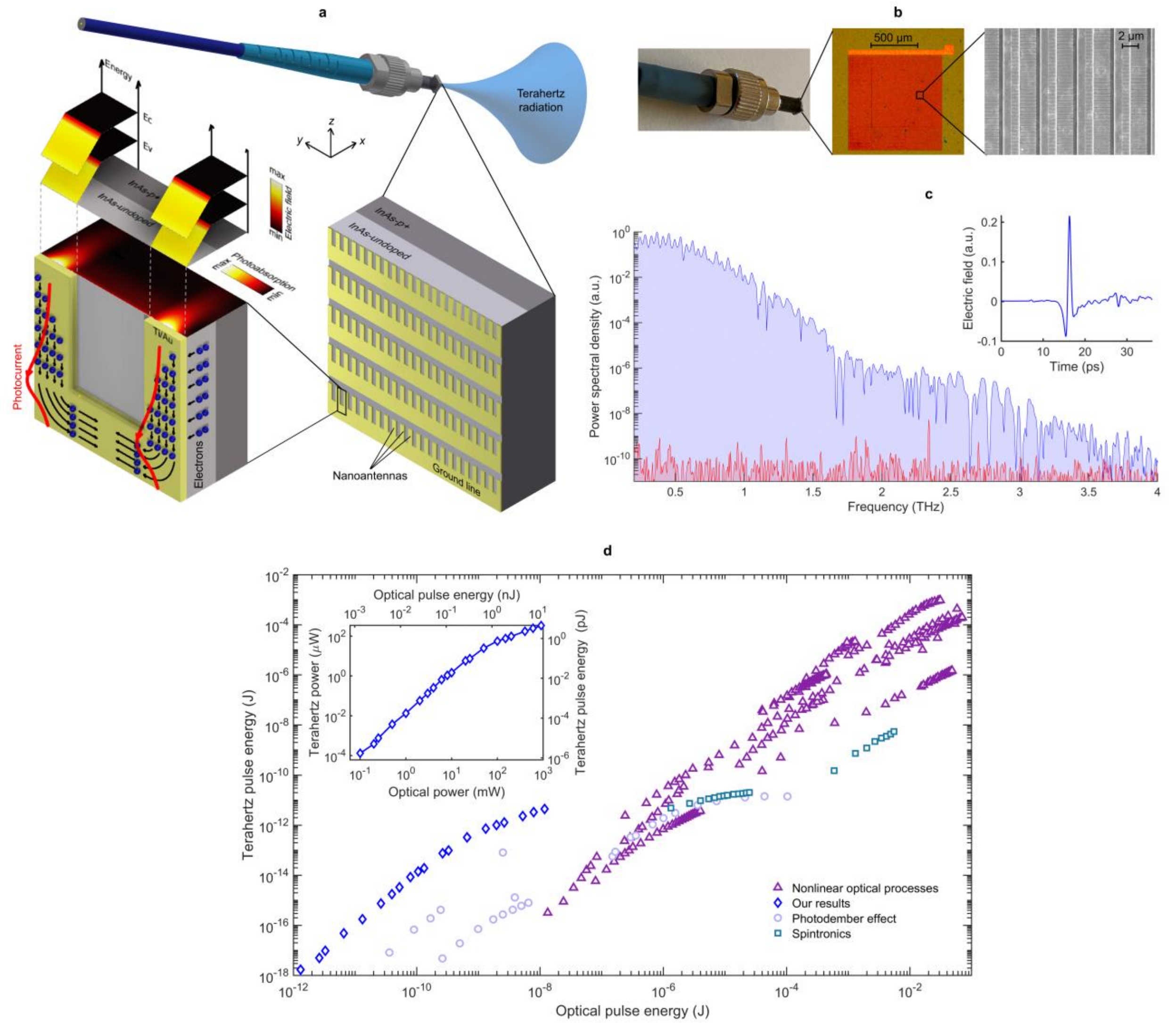

Figure 11: (a) Schematic of the bias-free terahertz emitter based on plasmon-coupled surface states. (b) Photograph, microscopy, and scanning electron microscopy images of a fabricated emitter prototype. (c) Measured terahertz radiation power (in blue) and noise (in red) spectra generated from the fiber-coupled emitter. (d) Measured terahertz pulse energy/power from the fabricated emitter as a function of the optical pulse energy/power (inset) in comparison with other passive optical-to-terahertz converters reported in the literature. Adapted from [97].

Engineering the doping profile of the photo-absorbing semiconductor is not the only way to realize the bias-free emitter concept. It has been demonstrated that the use of a graded bandgap ~~also~~ leads to a built-in electric field that extends throughout the entire photoconductive layer, allowing all of the photo-generated carriers to be accelerated towards the nanoantennas at the surface [~~113~~115]. In particular, an InGaAs layer with a linearly graded Indium composition from 60% to 100% was epitaxially grown on an SI-GaAs substrate. This design results in a stronger photocurrent and, hence, a ~~~four~~4 times higher radiated terahertz power than achieved in ~~[95~~ref. [97]. However, due to a lower electric field intensity near the surface, which leads to slower peak carrier velocity and slightly reduced high frequency radiation, the enhancement of total power comes with a small trade-off in its bandwidth. Further improvements in the

performance of this bias-free emitter concept are anticipated to be possible by the combination of the two design approaches.

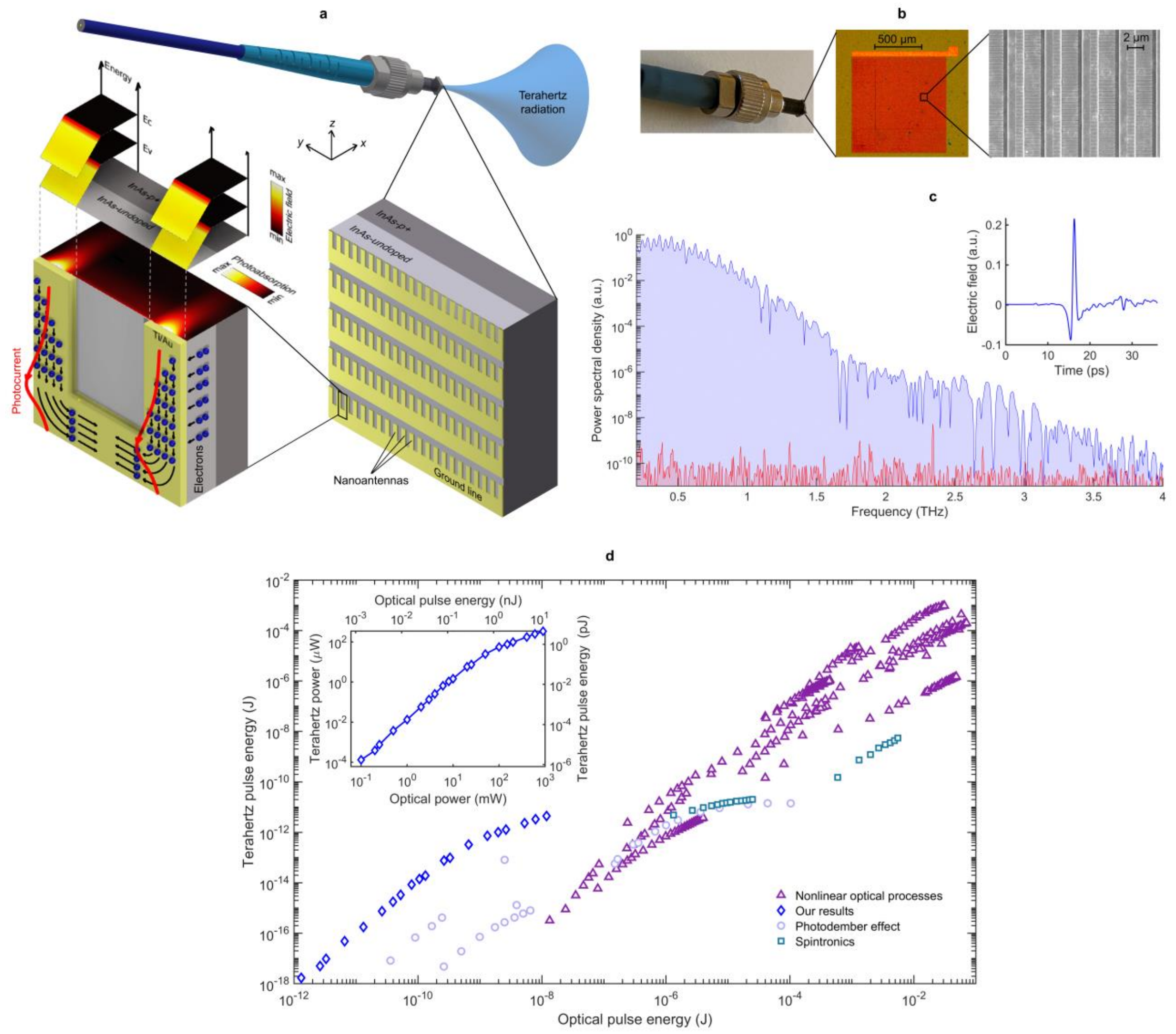

Figure 9: (a) Schematic of the bias-free terahertz emitter based on plasmon-coupled surface states. (b) Photograph, microscopy, and scanning electron microscopy images of a fabricated emitter prototype. (c) Measured terahertz radiation power (in blue) and noise (in red) spectra generated from the fiber-coupled emitter. (d) Measured terahertz pulse energy/power from the fabricated emitter as a function of the optical pulse energy/power (inset) in comparison with other passive optical-to-terahertz converters reported in the literature [95].

In summary, photoconductors and photomixers with embedded plasmonic nanostructures

- Enable broadband terahertz generation and detection under high photoconductive gain.
- Enable record-high-power pulsed terahertz generation compared to the state-of-the-art photoconductive sources [101, 115].
- Enable record-high-sensitivity pulsed terahertz detection at very low optical pump powers compared to the state-of-the-art photoconductive detectors [48, 49].
- Are widely used for both pulsed and CW operation at different optical excitation wavelengths, through a wide range of semiconductor substrates without requiring defect engineering.
- Require more demanding nanofabrication processes to be fabricated.

3.C Photomixers enabled by spintronics

The research field of spintronics aims at complementing and enhancing the functionality of conventional electronic devices, which are based on the charge of the electron, by the spin degree of freedom of the electron [~~114~~116]. In terms of applications in terahertz photonics, spintronic effects have been shown to be highly useful for the generation and amplitude modulation of terahertz electromagnetic waves [55, 56, ~~115~~117]. In this section, we will focus on spintronic terahertz emitters (STEs) because they take advantage of ultrafast optically induced electron and spin dynamics.

Operation of spintronic terahertz emitters

Recently, STEs have emerged as a novel concept for efficient and broadband generation of terahertz pulses [56]. STEs are based on two spintronic key phenomena (Fig. ~~10a~~12a): optically induced terahertz spin transport ~~(TST) [116~~[118] and spin-to-charge-current conversion (S2C) [~~117]. TST~~119]. Terahertz spin transport can be driven by gradients of temperature or spin voltage [~~118~~120]. The latter is also known as spin accumulation, which can be understood as an excess of spin density. S2C relies on spin-orbit interaction~~ (SOI),~~, which makes the motion of electrons spin-dependent. An important example is the inverse spin Hall effect~~ (ISHE),~~, which transforms a spin current into a perpendicularly oriented charge current [~~119~~121]. As with any optically driven terahertz emitters, STEs transform a driving femtosecond laser pulse into an ultrafast charge-current burst that acts as a source of an electromagnetic pulse. A typical STE [56] consists of nonmagnetic (NM) and ferromagnetic (FM) metallic layers that are just a few nanometers thick~~.~~ (Figs. 12a and 12b). The FM layer has an in-plane magnetization M and is made of Fe, Co or Ni or alloys thereof. The NM layer is usually made of a heavy metal with large ~~ISHE~~inverse spin Hall effect, for instance Pt or W. Illumination of the FM|NM stack with a femtosecond laser pulse triggers ~~TST~~terahertz spin transport from the FM layer into the NM layer. The resulting out-of-plane spin current j_s is converted into an ultrashort in-plane charge current j_c, predominantly by the ~~ISHE~~inverse spin Hall effect in the NM layer. This emerging charge-current burst emits electric-dipole radiation with frequencies extending into and beyond the terahertz range.

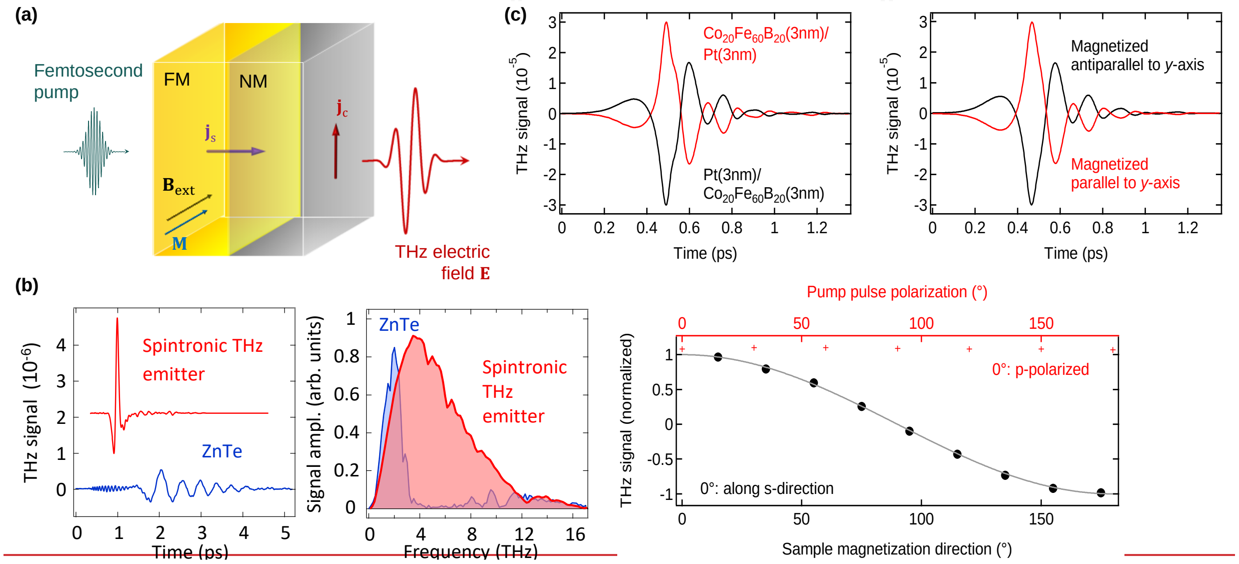

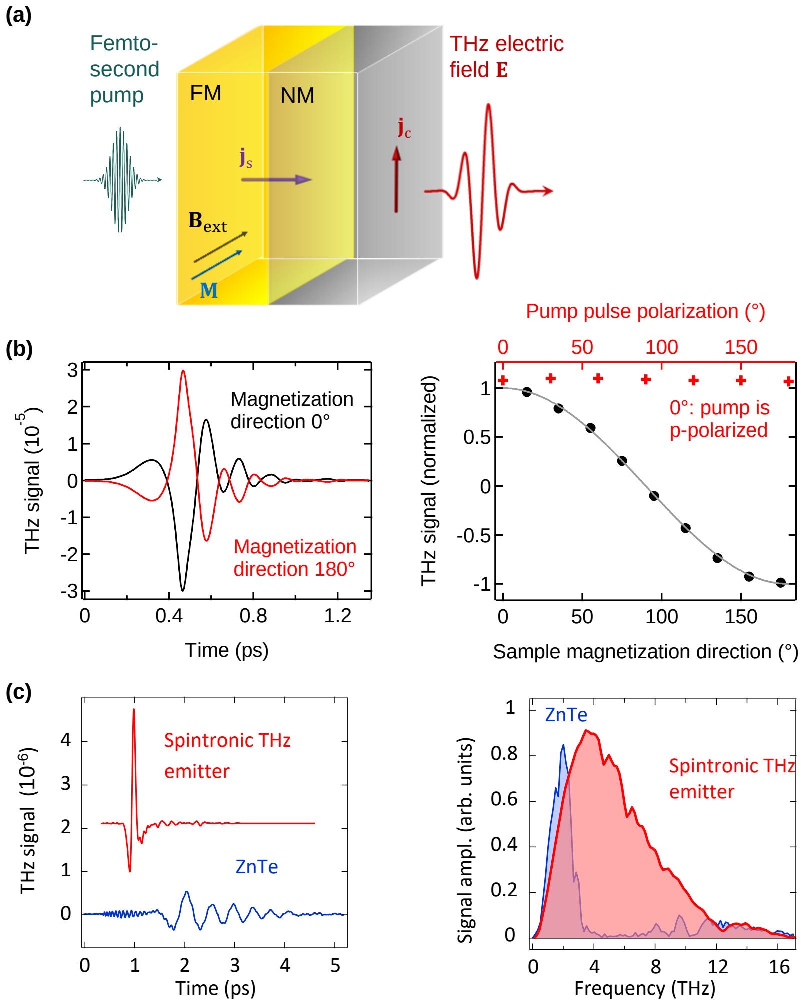

Figure ~~10~~12: (a) Schematic of ~~an~~ a spintronic terahertz emitter (STE~~.~~). A femtosecond pump pulse drives ~~an out-of-plane~~terahertz spin ~~current j_s~~transport from the ferromagnetic (FM) into the nonmagnetic (NM) metallic layer. ~~Its~~The spin polarization of the resulting out-of-plane spin current is determined by the in-plane sample magnetization ~~M~~, which is set by the external magnetic field ~~B_{ext}.~~. Spin-to-charge-current conversion~~,~~ (S2C), predominantly by the inverse spin Hall effect, converts ~~j_s~~ into an in-plane charge current ~~j_c,~~, which emits a terahertz pulse. (b) Terahertz signal symmetries. Impact of opposite sample magnetizations (left panel) and of the pump polarization (right panel, red symbols) and the in-plane sample magnetization orientation on the emitted terahertz-pulse polarization (black symbols). (c) Typical terahertz signal waveforms and their spectra as obtained from a STE and a conventional ZnTe terahertz emitter under identical conditions. The waveforms were detected by electrooptic sampling in a 70 ~~μm thick LAPC sensor [56]. (c) Terahertz signal symmetries. Impact of growth direction of the spintronic bilayers, that is FM|NM vs NM|FM stacks (top left panel), of opposite sample magnetizations (top right panel) and of the pump polarization (bottom panel, red symbols) and the in-plane sample magnetization orientation on the emitted terahertz-pulse polarization (black symbols). Figures are adapted from [56].~~ μm thick sensor made of Lemke/amorphous polycarbonate. Reprinted (adapted) by permission from [56]. Copyright (2016) by Springer Nature.

Fig. ~~10b~~12c shows a typical terahertz waveform and its spectrum as obtained from a W(2 nm)|CoFeB(1.8 nm)|Pt(2 nm) trilayer STE with laser pulses (duration 10 fs, center wavelength 800 nm, pulse energy ~1 nJ, repetition rate 80 MHz) from a Ti:sapphire oscillator. As compiled in the following, the STE concept offers unique features that make this class of terahertz emitters very promising for applications in the field of terahertz photonics~~.~~:

1. ~~Ultrabroadband~~Ultra-broadband operation in the range from ~1 to 40 THz without spectral gaps [56].

2. Optical-to-terahertz conversion efficiencies exceeding those of standard nonlinear terahertz emitters such as GaP or ZnTe (Fig. ~~10b~~12c) [56].
3. Independence of the pump-pulse wavelength and polarization (Fig. ~~10c~~12b) making STEs suitable for excitation with low-cost fiber laser-systems [~~120, 121~~122, 123].
4. Excellent long-term stability.
5. Linearly polarized terahertz output (Fig. ~~10c~~12b), whose polarization plane can be easily set by an external magnetic field at rates of 10 kHz and above [~~115~~117].
6. Capability of realizing more complex spatial terahertz polarization patterns by tuning the magnetization landscape inside STEs [~~122~~124].
7. Collinear pump and terahertz beam propagation as well as inheritance of any pump beam characteristics such as divergence and beam profile.
8. Possibility of material growth on large-scale, curved and/or flexible substrates by well-established thin-film fabrication techniques [~~123~~125]. Using upscaling, large terahertz peak field strengths of 300 kV/cm were demonstrated [~~124~~126].
9. Straightforward microstructuring of the thin metal films, enabling photonic tuning of the pump-absorption and terahertz-emission characteristics of STEs [~~125, 140~~127, 142].
10. Enabling a promising near-field imaging approach with a spatial resolution that is only limited by the spatial resolution of the pump (rather than by the terahertz wavelength) [~~126~~128].

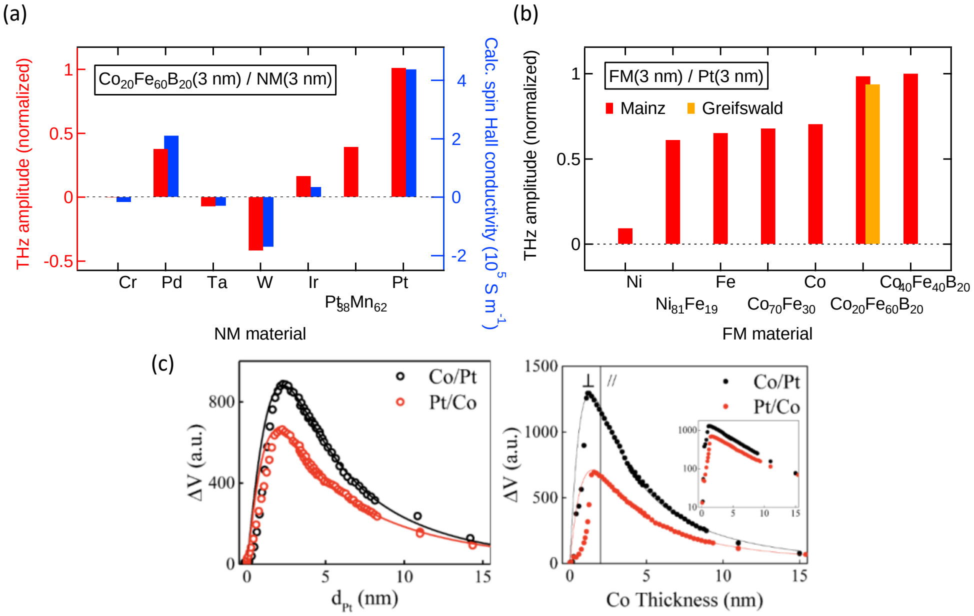

Figure ~~11~~13: (a) NM material variation. Dependence of the terahertz emission amplitude from $Co_{20}Fe_{60}B_{20}$|NM bilayers on the NM material (red bars), along with the calculated values of the spin-Hall conductivity (blue bars). (b) FM material variation. Dependence of the terahertz emission amplitude from spintronic bilayers on the FM material. Reprinted (adapted) by permission from [56]. Copyright (2016) by Springer Nature. (c) Thickness dependence. Terahertz-emission amplitude for varying NM (left) and FM (right) thicknesses for FM/NM and reversely grown bilayers. ~~Figures are~~ Reprinted (adapted) by permission from [~~56, 127].~~129]. Copyright (2018) by American Physical Society.

In more detail, the terahertz emission process indicated by Fig. ~~10a~~12a can be divided into 4 steps [~~128~~130]: (i) pump-light absorption, (ii) spin-current generation and injection into the NM layer, (iii) S2C by the ~~ISHE~~inverse spin Hall effect and (iv) electromagnetic-wave emission of the resulting charge current into the far-field. For the NM and FM layer film thicknesses much smaller than the terahertz attenuation

length and the terahertz wavelength, the terahertz electric field directly behind the STE (i.e. in air) can be calculated in the frequency domain by a generalized Ohm's law [56]:

$$E(\omega) = eZ(\omega) \int \gamma(z,\omega) j_{\mathrm{s}}(z,\omega)\,\mathrm{d}z. \tag{18}$$

Here, $j_{\mathrm{s}}(z,\omega)$ is the spin-current density (dimension electrons/m^2 s) as a function of spatial position z (along the surface normal) and frequency $\omega/2\pi$. It captures steps (i) and (ii). S2C results in the charge-current density $\gamma(z,\omega)j_{\mathrm{s}}(z,\omega)$, capturing step (iii), and its local strength is quantified by $\gamma(z,\omega)$. When S2C is dominated by the ~~ISHE~~inverse spin Hall effect, γ is called spin Hall angle and approximately constant between 1 and 40 THz [~~129~~131]. Finally, $Z(\omega)$ is the STE impedance and quantifies step (iv), the current-to-electric-field conversion. It is given by [56]

$$Z(\omega) = \frac{Z_0}{n_1(\omega) + n_2(\omega) + Z_0 G(\omega)}, \tag{19}$$

with the free-space impedance $Z_0 \approx 377\ \Omega$, the refractive indices $n_1(\omega) \approx 1$ and $n_2(\omega)$ of air and the substrate, respectively, and the terahertz sheet conductance $G(\omega)$ of the metal stack.

We note that the previous consideration refers to stack-like STEs with in-plane homogeneity (Fig. ~~10a~~12a). It is still approximately valid for pump-spot diameters smaller than the terahertz vacuum wavelength $\lambda = 2\pi c/\omega$, where c is the vacuum speed of light [~~129~~131]. For more complicated geometries, for example, sub-λ-sized STEs combined with antenna structures, a more elaborate modeling of the emission process (iii) is required [~~140~~142].

The STE model summarized by Eqs. ~~17~~18 and ~~18~~19 shows that the spintronic terahertz-pulse-generation performance is strongly material-dependent. More specifically, the material parameters that determine the terahertz-emission strength are $n_1(\omega)$, $n_2(\omega)$, $\gamma(\omega)$ and $G(\omega) = \int_0^{d_{\mathrm{FM}}+d_{\mathrm{NM}}} \sigma(z,\omega)\mathrm{d}z$, that is, the metal film thicknesses d_{FM} and d_{NM} as well as their respective conductivity σ. The factor $j_{\mathrm{s}}(z,\omega)$ depends on the efficiency of spin current is generation upon deposition of pump-pulse energy, on the spin-current relaxation length λ_{NM} inside the NM material and on the FM/NM interface spin transmissivity. Accordingly, a manifold of recent studies aimed at maximizing the STE performance by tuning one of these parameters.

1) Optimization of FM and NM: It turned out that in terms of S2C efficiency, the ~~ISHE NM~~spin-Hall materials Pt and W are the best choice for NM (Fig. ~~11a~~13a) [56, ~~123~~125]. On the FM side, Fe, Co, Ni and their alloys were studied. As of now, $Co_{40}Fe_{40}B_{20}$ alloys show the best performance as the STE's FM material (Fig. ~~11b~~13b) [56]. Yet, it should be noted that, for both, the NM or the FM material, a large variety of different materials were tested. For the S2C material, these materials cover topological insulators [~~130~~132], transition-metal dichalcogenides [~~131~~133], antiferromagnetic metals [~~132, 133~~134, 135] or inverse Rashba-Edelstein systems such as Bi/Ag interfaces [~~127, 134~~129, 136]. For the FM layer, also more complex magnetic materials beyond simple 3d ferromagnets and their alloys were studied, for instance ferrimagnets [~~135~~137] and magnetic insulators [~~136, 137~~138, 139].

2) Thickness optimization: According to Eqs. ~~17~~18 and ~~18~~19, the NM and FM layer thicknesses should be optimized with respect to $Z(\omega)$ and the optical excitation density that determines the magnitude of j_{s}. Importantly, as typical values of λ_{NM} are found to be in the range of a few nanometers only for NM materials with a large S2C efficiency, j_{s} is localized on a similar length scale around the FM/NM interface.

Thus, the optimal thicknesses for the FM and NM layers are found in the range between 2 to 5 nm (Fig. ~~11c~~13c) [56, ~~123,~~ 125, ~~128, 138, 139~~127, 130, 140, 141]: they maximize $Z(\omega)$ and are still thick enough to prevent sizeable spin-current back-reflections at the STE/substrate or STE/air interfaces and, thus, reduction of j_s.

3) Nano- and microstructuring: STEs are made of thin metal films and, thus, perfectly suited for nano- and microstructuring approaches. Accordingly, trilayer STEs of the form NM1|FM|NM2 were found to boost the STE efficiency by a factor of almost two. The reason for this increased performance is that both the forward- and backward-travelling spin currents can contribute to the terahertz emission, in contrast to the simple bilayer STE. The best STE performance was reported for trilayers W|$Co_{40}Fe_{40}B_{20}$|Pt that can compete with conventional nonlinear optical terahertz emitters such as ZnTe and GaP in terms of terahertz amplitude yet with a drastically enhanced terahertz bandwidth [56]. Microstructuring in the plane of the STE allows to incorporate terahertz antenna designs that were demonstrated to enhance the emitter performance in certain spectral ranges [~~125, 140, 141~~127, 142, 143].

Applications of spintronic terahertz emitters

In the following, we briefly summarize the most representative applications of STEs. STEs are an ideal source to cover the entire range between 1 and 30 THz within a single experiment. Accordingly, STEs were successfully applied for broadband linear terahertz transmission spectroscopy of dielectrics such as Teflon tape [56], several perovskites [~~142, 143~~144, 145], quartz [~~144~~146] and water [~~145~~147]. They were also used to probe the terahertz conductivity and the S2C conversion efficiency in ferrimagnetic metals [~~129~~131] and the terahertz anisotropic magnetoresistance of ferromagnetic metals [~~146~~148]. Spatial and temporal modulation of the external magnetic field was shown to allow for generating doughnut-type terahertz beams [~~122~~124], tuning the helicity of the terahertz radiation [~~147-~~149-151], 10-kHz modulation of the terahertz field orientation [~~115~~117], or the change of the terahertz beam divergence by the growth on flexible substrates [~~123~~125]. Near-field imaging approaches promise deep subwavelength spatial resolution and are easily realized within the STE concept. In this way, a spatial resolution of 6.5 µm was demonstrated, which is about 50 times smaller than the wavelength at 1 THz [~~126~~128]. Related approaches succeeded in sensing cancer cells on the STE surface [~~150~~152] or used the STE as a local probe of static magnetic fields with millitesla and submillimeter resolution [~~151~~153]. Nonlinear terahertz spectroscopy with the STE was enabled by large-scale terahertz emitters [~~124~~126] with peak electric fields of 300 kV/cm. Finally, the combination of the STE with a scanning tunneling microscope [~~152~~154] enabled atomic spatial and femtosecond temporal resolution to probe emerging phenomena at the nanoscale.

The spintronic principles behind STEs allow for a rapid implementation of novel material classes into this terahertz emitter class. Examples include semiconductors that feature outstandingly large values of λ_{NM} [~~153~~155], topological materials that may boost S2C efficiencies by interface engineering [~~130~~132] as well as two-dimensional materials such as transition-metal dichalcogenides that would enable novel S2C phenomena such as the valley Hall effect [~~154~~156]. Eventually, antiferromagnetic materials promise intrinsic terahertz dynamics [~~155~~157], which might allow one to engineer tunable narrow-band STEs in the future [~~156~~158]. These and other improvements of STEs including photonic engineering [47, ~~157~~159] or interface tailoring [~~158~~160] along with the recent commercial availability of STEs (TeraSpinTec GmbH) point toward promising upcoming developments in the field of spintronic terahertz photonics. On a short-term basis, open directions in the field include enhancing the STE efficiency to reach the performance of photoconductive antennas at terahertz frequencies below 3 THz, demonstrating a CW operation and full

implementation of STEs into on-chip designs [~~159~~161]. Last but not least, realizing a spintronic terahertz detector, yet being elusive, would complement the ultrabroadband spintronic terahertz-spectroscopy toolbox.

In summary, spintronic terahertz photomixers

- Enable ultra-broadband terahertz radiation (1-40 THz) with optical-to-terahertz conversion efficiencies exceeding conventional nonlinear optical terahertz emitters.
- Operate independent of the incident optical wavelength and polarization, while the emitted terahertz polarization can be easily controlled by an external magnetic field.
- Are easy to fabricate using mature thin-film growth techniques, allowing straightforward microstructuring to optimize optical absorption and/or terahertz emission characteristics.
- Have yet to provide comparable terahertz power levels with state-of-the-art photoconductive emitters under the same optical pump power.
- Have yet to enable CW terahertz generation and CW/pulsed terahertz detection.

3.D Photoconductors and photomixers based on low-dimensional nanomaterials

Many low-dimensional nanomaterials provide unique properties that can be utilized for terahertz applications. In particular, graphene's broadband optical absorption, high carrier mobility (up to 70000 $cm^2/V/s$ under room temperature [~~160~~162]), as well as ultrafast hot carrier dynamics [~~161~~163] have been of great interest, enabling the realization of terahertz emitters and detectors [57, ~~162-165~~164-167]. Moreover, many low-dimensional nanomaterials, such as graphene and nanowires, are easily transferrable to other semiconductor substrates, facilitating their integration with other optical and electronic systems.

Low dimensional nanomaterials have been used as nanoelectrodes to boost the performance of conventional photoconductive antennas. In ~~[166]~~ref. [168], the authors utilized silver nanowires as electrodes to provide high current handling capability and considerably reduced electrode capacitance for an LT-GaAs photoconductive antenna, as a substitute for the usual finger structure used for CW operation. Further incorporation of n^+ single layer graphene flakes deposited randomly on the photoconductive material enhanced the optical pump absorption due to plasmonic effects. Overall, a 7-fold increase in the photocurrent and a 2-fold increase in the radiated terahertz power were obtained compared to a reference photoconductive antenna from the same material with finger electrodes.

Using low dimensional nanomaterials as the active photoconductive layer is an alternative design approach to exploit their unique optical and transport properties. In a recent work, single-layer graphene and ultrathin graphite were optically gated to sample an incoming terahertz field, resulting in more than 2 THz detection bandwidth (Fig. ~~12a) [164~~14a) [166]. With much higher optical pump absorption, the ultrathin-graphite-based detector provided higher sensitivity compared to the single-layer graphene detector. Importantly, the ultrathin graphite films were realized by a transfer-free direct deposition process, which brings about much better scalability. In addition to free space terahertz devices, guided-wave graphene-based terahertz spectroscopic systems have also been realized in order to achieve stronger terahertz field confinement. In [~~165~~167], on-chip terahertz generation and detection through Goubau transmission lines were demonstrated. Thanks to the ultrafast dynamics of the photogenerated hot carriers, the graphene terahertz detector is shown to offer a similar performance compared to an LT-

GaAs-based terahertz detector, both with ~ 0.6 THz bandwidth as shown in Fig. ~~12b~~14b. The bandwidth limitation is attributed to the dispersion of the on-chip transmission lines that broadens~~ of~~ the detected terahertz pulses.

Similar to graphene, black phosphorus is another two-dimensional material that has been intensively studied for terahertz emission and detection [~~167-~~169-171]. A coherent photoconductive detector based on exfoliated multilayer black phosphorus has been demonstrated with a usable bandwidth up to 0.2 THz and a peak dynamic range of 40 dB in conjunction with a GaAs-based photoconductive emitter [~~169~~171].

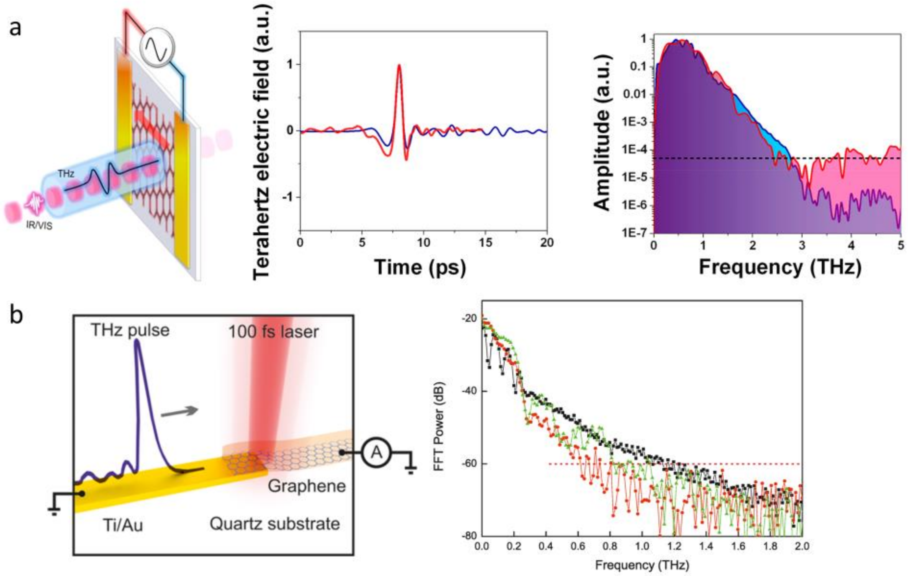

Figure ~~12~~14: (a) Schematic of the graphitic photoconductive terahertz detector (left), the measured time-domain electric field (middle), and the amplitude spectra (right) from the graphitic detector (in red) as compared to the reference data measured by conventional electro-optic sampling (in blue). The dashed black line indicates the noise level of the graphitic detector measurement. Reprinted (adapted) by permission from [166]. Copyright (2019) by American Chemical Society. (b) Schematic of the guided-wave graphene photoconductive terahertz detector (left) and the measured terahertz power spectra (right) at the transmission line input by LT-GaAs (black squares), at the transmission line output by graphene (red circles), and at the transmission line output by LT-GaAs (green triangles). The dashed red line indicates the noise level. ~~Figures are~~ Adapted from [~~164, 165~~167].

Semiconductor nanowires have also been utilized to realize terahertz detectors. Similar to bulk III-V semiconductors, most III-V semiconductor nanowires possess a direct bandgap [~~170~~172] and high carrier mobility [~~171, 172~~173-175]. Moreover, due to nanometer scale of their width, they offer very high dark resistivity and high spatial resolution for terahertz detection [59, ~~173~~176]. The first demonstration of a single nanowire terahertz detector in a time-domain spectroscopy system incorporated GaAs/AlGaAs core–shell nanowires and provided up to a 0.6 THz detection bandwidth as shown in Fig. ~~13a~~15a, where the bandwidth limitation ~~originates~~originated from the detector design rather than the nanowire response [59]. A later work developed a terahertz detector using InP nanowires with high crystal quality [~~173~~176]. Combined with an optimized antenna geometry, the detector offered a usable detection bandwidth of 2 THz (Fig. ~~13b~~15b). It is important to note that the detection bandwidth is not limited by the long carrier lifetime of the nanowire (1.71 ns) since the incoming terahertz electric field can still be recovered knowing that the detector acts as an electric field integrator with an optically-gated ultrafast switch-on transition. Further improvement of the device was achieved by engineering the axial doping profile of the nanowire, leading to a significantly reduced contact resistance and a 2.5-fold increase in the signal-to-noise ratio [~~174~~177]. As shown in Fig. ~~13c~~15c, the high integrability of nanowire terahertz

detectors was recently demonstrated with a monolithic three-dimensional cross-nanowire network that was able to sample the orthogonal components of the terahertz electric field simultaneously [~~175~~178]. Using two pairs of electrically isolated perpendicular InP nanowires, the complete polarization state of an incoming terahertz radiation was accurately determined within a single time-domain scan with little cross-talk. These demonstrations show the great potentials of cross-nanowire detectors for advancing terahertz ellipsometry, anisotropic spectroscopy, as well as polarization-resolved spectral imaging.

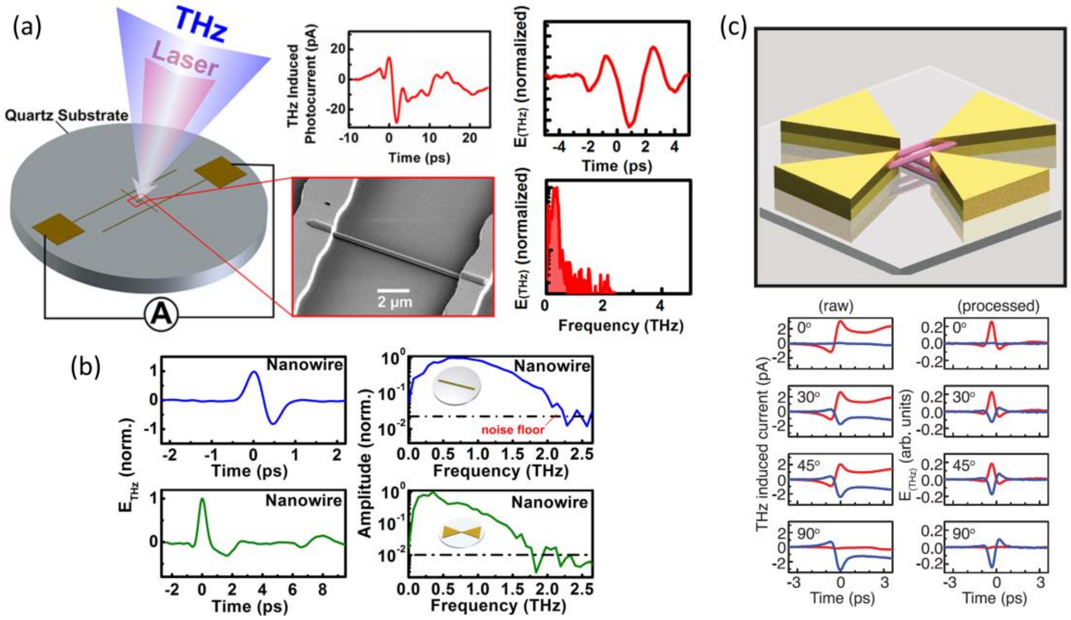

Figure ~~13~~15: (a) Schematic and scanning electron microscopy image of the GaAs/AlGaAs core−shell nanowire detector, as well as the experimentally measured terahertz induced photocurrent (top middle), extracted time-domain electric field (top right), and amplitude spectrum (bottom right). Adapted from [59]. (b) Extracted time-domain electric field and amplitude spectra of InP nanowire detectors based on a strip-line antenna (blue) and a bowtie antenna (green). Adapted from [176]. (c) Schematic of the cross-nanowire detector (top) and the measured induced photocurrents as well as extracted electric field under different incident linear terahertz polarization. Red and blue colors indicate the response from the horizontal and vertical detection channels, respectively. ~~Figures are adapted from [59, 174, 176].~~Reprinted (adapted) by permission from [178]. Copyright (2020) by American Association for the Advancement of Science.

In summary, low-dimensional nanomaterials

- Enable high carrier mobility and/or ultrafast relaxation dynamics that facilitate both terahertz generation and detection.
- Are easily transferrable to a wide range of substrates, allowing integration with other optical and electronic systems.
- Are so far limited in operation bandwidth, emission power, and detection sensitivity due to their low quantum efficiency.

4. Conclusion and outlook

In this review, we provided a thorough overview of various photonics-driven physical mechanisms that give rise to ultrafast carrier dynamics to be utilized for terahertz generation and detection without relying on conventional defect-introduced short-carrier-lifetime semiconductors. ~~In~~While quite a few excellent results have been achieved by using conventional short-carrier-lifetime semiconductors, the high defect concentrations ~~are~~ intentionally incorporated in the active photo-absorbing material~~, leading~~

lead to lower carrier mobility and substantial degradation of photoconductive gain due to a large number of photocarriers being scattered, trapped and recombined. Additionally, ~~they often~~many realizations of short-carrier-lifetime materials have limited accessibility due to the need for non-standard processes, as well as rare elements as defect-introducing dopants. Therefore, alternative approaches to realize terahertz emitters and detectors have attracted increasing attention, where this review has covered the most representative categories, including carrier transit time reduction in ~~p-i-n junctions~~UTC-PD structures, plasmonic enhancement and confinement of optical generation, spin-to-charge current conversion, as well as fast relaxation and low-noise sensing in low-dimensional materials. The state-of-the-art device performance based on each mechanism is discussed and summarized, with an emphasis on the key physical processes that enable terahertz generation and detection~~. In principle, these terahertz devices convert the frequency components of the optical pump beam into the terahertz frequency range – the so-called photomixing process – facilitated by the ultrafast transport of photocarriers. As a result, they can take advantage of the extremely wide bandwidth of the optical frequency range and the mature technology of optical lasers in both pulsed and CW operation. Especially, many of the terahertz photomixers discussed in this review allow a broad range of operation wavelengths, which enables a large design flexibility and the applicability of the devices. It is anticipated that~~, as well as how they differ from conventional defect-introduced short-carrier-lifetime semiconductors. Specifically, the use of UTC-PDs and plasmonic nanostructures provide high photoconductive gain and efficient utilization of photocarriers, leading to record-high terahertz power levels in CW and pulsed operation. On the other hand, spintronic materials and low-dimensional nanomaterials provide major advantages for terahertz operation due to their unique properties that are not attainable in bulk semiconductors. In particular, thanks to the ultrafast spin dynamics, spintronic emitters feature ultra-broadband radiation (1-40 THz) without spectral gaps, with optical-to-terahertz conversion efficiencies exceeding conventional nonlinear optical terahertz emitters. As for low-dimensional nanomaterials, their high carrier mobility and/or ultrafast carrier relaxation dynamics have enabled the implementation of various emitters and detectors operating in the lower end of the terahertz spectrum.

Further investigation and improvement of these design approaches will continue revealing more potentials. For example, so far UTC-PDs and spintronic devices have been exclusively used for CW and pulsed terahertz generation, respectively. The lack of terahertz detection capability could limit their use for certain applications that require monolithic integration of emitters and detectors on the same chip. In this regard, plasmonic-enhanced devices offer great flexibility to be used for both CW/pulsed terahertz generation and detection. However, while UTC-PDs and spintronic devices can be manufactured with standard and less demanding processes, fabrication of plasmonic-enhanced devices relies on sophisticated nanofabrication techniques (e.g., electron beam lithography) due to their submicron feature sizes. This requirement increases their fabrication cost and lowers their fabrication throughput, unless cheaper and more scalable alternative nano-manufacturing techniques become mature and widely available. Lastly, while the relatively low optical absorption in low-dimensional nanomaterials has so far limited their use for high-performance terahertz emission and detection, new innovations on enhanced light-matter interaction are expected to gradually alleviate this limitation. It is highly anticipated that all of the design approaches covered in this review will continue to be further developed and optimized, hence extending the scope and widespread usage of terahertz technology to many practical applications.

Acknowledgments

WeJarrahi’s group gratefully acknowledgeacknowledges the financial support of the US Department of Energy (grant # DE-SC0016925). Kampfrath’s group acknowledges funding by the German Research Foundation through the collaborative research center SFB TRR 227 “Ultrafast spin dynamics” (projects B02 and A05), the European Union H2020 program through the project CoG TERAMAG/Grant No. 681917, and financial support from the Horizon 2020 Framework Program of the European Commission under FET-Open Grant No. 863155 (s-Nebula).

References

[1] M. Van Exter, C. Fattinger, and D. Grischkowsky. "Terahertz time-domain spectroscopy of water vapor." *Optics letters* 14, no. 20 (1989): 1128-1130.

[2] A. Markelz, S. Whitmire, J. Hillebrecht, and R. Birge. "THz time domain spectroscopy of biomolecular conformational modes." *Physics in Medicine & Biology* 47, no. 21 (2002): 3797.

[3] Y. C. Shen, T. Lo, P. F. Taday, B. E. Cole, W. R. Tribe, and M. C. Kemp. “Detection and identification of explosives using terahertz pulsed spectroscopic imaging." *Applied Physics Letters* 86, no. 24 (2005): 241116.

[4] D. M. Mittleman, R. H. Jacobsen, R. Neelamani, R. G. Baraniuk, and M. C. Nuss. "Gas sensing using terahertz time-domain spectroscopy." *Applied Physics B* 67, no. 3 (1998): 379-390.

[5] M. Kato, S. R. Tripathi, K. Murate, K. Imayama, and K. Kawase. "Non-destructive drug inspection in covering materials using a terahertz spectral imaging system with injection-seeded terahertz parametric generation and detection." *Optics express* 24, no. 6 (2016): 6425-6432.

[6] A. Redo-Sanchez, B. Heshmat, A. Aghasi, et al. "Terahertz time-gated spectral imaging for content extraction through layered structures." *Nature communications* 7, no. 1 (2016): 1-7.

[7] M. C. Kemp, P. F. Taday, B. E. Cole, J. A. Cluff, A. J. Fitzgerald, and W. R. Tribe. “Security applications of terahertz technology." In *Terahertz for Military and Security Applications*, vol. 5070, pp. 44-52. International Society for Optics and Photonics, 2003.

[8] R. Ulbricht, E. Hendry, J. Shan, T. F. Heinz, and M. Bonn. "Carrier dynamics in semiconductors studied with time-resolved terahertz spectroscopy." *Reviews of Modern Physics* 83, no. 2 (2011): 543.

[9] S. E. Ralph, S. Perkowitz, N. Katzenellenbogen, and D. Grischkowsky. "Terahertz spectroscopy of optically thick multilayered semiconductor structures." *JOSA B* 11, no. 12 (1994): 2528-2532.

[10] B. B. Hu, and M. C. Nuss. "Imaging with terahertz waves." *Optics letters* 20, no. 16 (1995): 1716-1718.

[11] M. Tonouchi. "Cutting-edge terahertz technology." *Nature photonics* 1, no. 2 (2007): 97-105.

[12] B. Ferguson, and X.-C. Zhang. "Materials for terahertz science and technology." *Nature materials* 1, no. 1 (2002): 26-33.

[13] P. U. Jepsen, D. G. Cooke, and M. Koch. "Terahertz spectroscopy and imaging–Modern techniques and applications." *Laser & Photonics Reviews* 5, no. 1 (2011): 124-166.

[14] E. Pickwell, and V. P. Wallace. "Biomedical applications of terahertz technology." *Journal of Physics D: Applied Physics* 39, no. 17 (2006): R301.

[15] S. Fan, Y. He, B. S. Ung, and E. Pickwell-MacPherson. "The growth of biomedical terahertz research." *Journal of Physics D: Applied Physics* 47, no. 37 (2014): 374009.

[16] R. M. Woodward, B. E. Cole, V. P. Wallace, et al. "Terahertz pulse imaging in reflection geometry of human skin cancer and skin tissue." *Physics in Medicine & Biology* 47, no. 21 (2002): 3853.

[17] V. P. Wallace, P. F. Taday, A. J. Fitzgerald, et al. "Terahertz pulsed imaging and spectroscopy for biomedical and pharmaceutical applications." *Faraday Discussions* 126 (2004): 255-263.

[18] E. Castro-Camus, M. Palomar, and A. A. Covarrubias. "Leaf water dynamics of Arabidopsis thaliana monitored in-vivo using terahertz time-domain spectroscopy." *Scientific reports* 3, no. 1 (2013): 1-5.

[19] F. Rutz, M. Koch, S. Khare, M. Moneke, H. Richter, and U. Ewert. "Terahertz quality control of polymeric products." *International Journal of Infrared and Millimeter Waves* 27, no. 4 (2006): 547-556.

[20] M. Scheller, C. Jansen, and M. Koch. "Analyzing sub-100-μm samples with transmission terahertz time domain spectroscopy." *Optics Communications* 282, no. 7 (2009): 1304-1306.

[21] S. Krimi, J. Klier, J. Jonuscheit, G. Von Freymann, R. Urbansky, and R. Beigang. "Highly accurate thickness measurement of multi-layered automotive paints using terahertz technology." *Applied Physics Letters* 109, no. 2 (2016): 021105.

[22] P. C. Ashworth, E. Pickwell-MacPherson, E. Provenzano, et al. "Terahertz pulsed spectroscopy of freshly excised human breast cancer." *Optics express* 17, no. 15 (2009): 12444-12454.

[23] F. W. Smith, A. R. Calawa, C-L. Chen, M. J. Manfra, and L. J. Mahoney. "New MBE buffer used to eliminate backgating in GaAs MESFETs." *IEEE Electron Device Letters* 9, no. 2 (1988): 77-80.

[24] A. C. Warren, N. Katzenellenbogen, D. Grischkowsky, J. M. Woodall, M. R. Melloch, and N. Otsuka. "Subpicosecond, freely propagating electromagnetic pulse generation and detection using GaAs: As epilayers." *Applied physics letters* 58, no. 14 (1991): 1512-1514.

[25] E. R. Brown, F. W. Smith, and K. A. McIntosh. "Coherent millimeter-wave generation by heterodyne conversion in low-temperature-grown GaAs photoconductors." *Journal of Applied Physics* 73, no. 3 (1993): 1480-1484.

[26] S. Gupta, M. Y. Frankel, J. A. Valdmanis, et al. "Subpicosecond carrier lifetime in GaAs grown by molecular beam epitaxy at low temperatures." *Applied Physics Letters* 59, no. 25 (1991): 3276-3278.

[27] F. Ganikhanov, G-R. Lin, W-C. Chen, C-S. Chang, and C-L. Pan. "Subpicosecond carrier lifetimes in arsenic-ion-implanted GaAs." *Applied physics letters* 67, no. 23 (1995): 3465-3467.

[28] J. Lloyd-Hughes, E. Castro-Camus, M. D. Fraser, C. Jagadish, and M. B. Johnston. "Carrier dynamics in ion-implanted GaAs studied by simulation and observation of terahertz emission." *Physical Review B* 70, no. 23 (2004): 235330.

[29] J. F. O'Hara, J. M. O. Zide, A. C. Gossard, A. J. Taylor, and R. D. Averitt. "Enhanced terahertz detection via ErAs: GaAs nanoisland superlattices." *Applied physics letters* 88, no. 25 (2006): 251119.

[30] M. Griebel, J. H. Smet, J. Kuhl, et al. "Picosecond carrier dynamics in ErAs: GaAs superlattices." In *Ultrafast Electronics and Optoelectronics*, p. UWC5. Optical Society of America, 2001.

[31] V. Pačebutas, A. Bičiūnas, S. Balakauskas, et al. "Terahertz time-domain-spectroscopy system based on femtosecond Yb:fiber laser and GaBiAs photoconducting components." *Applied Physics Letters* 97, no. 3 (2010): 031111.

[32] V. Pačebutas, A. Bičiūnas, K. Bertulis, and A. Krotkus. "Optoelectronic terahertz radiation system based on femtosecond 1 μm laser pulses and GaBiAs detector." *Electronics Letters* 44, no. 19 (2008): 1154-1155.

[33] M. S. Kong, J. S. Kim, S. P. Han, et al. "Terahertz radiation using log-spiral-based low- temperature-grown InGaAs photoconductive antenna pumped by mode-locked Yb-doped fiber laser." *Optics express* 24, no. 7 (2016): 7037-7045.

[34] G. Matthäus, B. Ortaç, J. Limpert, et al. "Intracavity terahertz generation inside a high-energy ultrafast soliton fiber laser." *Applied Physics Letters* 93, no. 26 (2008): 261105.

[35] M. Sukhotin, E. R. Brown, D. Driscoll, M. Hanson, and A. C. Gossard. "Picosecond photocarrier-lifetime in ErAs: InGaAs at 1.55 μ m." *Applied physics letters* 83, no. 19 (2003): 3921-3923.

[36] U. Nandi, J. C. Norman, A. C. Gossard, H. Lu, and S. Preu. "1550-nm driven ErAs: In (Al) GaAs photoconductor-based terahertz time domain system with 6.5 THz bandwidth." *Journal of Infrared, Millimeter, and Terahertz Waves* 39, no. 4 (2018): 340-348.

[37] ~~ADJ F.~~A.D.J. Fernandez Olvera, H. Lu, A. C. Gossard, and S. Preu. "Continuous-wave 1550 nm operated terahertz system using ErAs: In (Al) GaAs photo-conductors with 52 dB dynamic range at 1 THz." *Optics Express* 25, no. 23 (2017): 29492-29500.

[38] B. Globisch, R. J. B. Dietz, R. B. Kohlhaas, et al. "Iron doped InGaAs: Competitive THz emitters and detectors fabricated from the same photoconductor." *Journal of Applied Physics* 121, no. 5 (2017): 053102.

[39] R. B. Kohlhaas, B. Globisch, S. Nellen, et al. "Rhodium doped InGaAs: A superior ultrafast photoconductor." *Applied Physics Letters* 112, no. 10 (2018): 102101.

[40] R. B. Kohlhaas, S. Breuer, L. Liebermeister, et al. "637 μW emitted terahertz power from photoconductive antennas based on rhodium doped InGaAs." *Applied Physics Letters* 117, no. 13 (2020): 131105.

[41] B. Sartorius, H. Roehle, H. Künzel, et al. "All-fiber terahertz time-domain spectrometer operating at 1.5 µm telecom wavelengths." *Optics express* 16, no. 13 (2008): 9565-9570.

[42] R. J. B. Dietz, B. Globisch, M. Gerhard, et al. "64 μW pulsed terahertz emission from growth optimized InGaAs/InAlAs heterostructures with separated photoconductive and trapping regions." *Applied Physics Letters* 103, no. 6 (2013): 061103.

[43] R. J. B. Dietz, B. Globisch, H. Roehle, D. Stanze, T. Göbel, and M. Schell. "Influence and adjustment of carrier lifetimes in InGaAs/InAlAs photoconductive pulsed terahertz detectors: 6 THz bandwidth and 90dB dynamic range." *Optics express* 22, no. 16 (2014): 19411-19422.

[44] T. Ishibashi, N. Shimizu, S. Kodama, H. Ito, T. Nagatsuma, and T. Furuta. "Uni-traveling-carrier photodiodes." In *Ultrafast Electronics and Optoelectronics*, p. UC3. Optical Society of America, 1997.

[45] H. Ito, F. Nakajima, T. Futura, and T. Ishibashi. "Continuous THz-wave generation using antenna-integrated uni-travelling-carrier photodiodes." *Semiconductor Science and Technology* 20, no. 7 (2005): S191.

[46] E. Rouvalis, C. C. Renaud, D. G. Moodie, M. J. Robertson, and A. J. Seeds. "Traveling-wave uni-traveling carrier photodiodes for continuous wave THz generation." *Optics Express* 18, no. 11 (2010): 11105-11110

[47] C. W. Berry, N. Wang, M. R. Hashemi, M. Unlu, and M. Jarrahi. "Significant performance enhancement in photoconductive terahertz optoelectronics by incorporating plasmonic contact electrodes." *Nature communications* 4, no. 1 (2013): 1-10.

[48] N. T. Yardimci, D. Turan, S. Cakmakyapan, and M. Jarrahi. "A high-responsivity and broadband photoconductive terahertz detector based on a plasmonic nanocavity." *Applied Physics Letters* 113, no. 25 (2018): 251102.

[49] N. T. Yardimci, D. Turan, and M. Jarrahi. "Efficient photoconductive terahertz detection through photon trapping in plasmonic nanocavities." *APL Photonics* 6, no. 8 (2021): 080802.

[50] N. T. Yardimci, S. Cakmakyapan, S. Hemmati, and M. Jarrahi. "A high-power broadband terahertz source enabled by three-dimensional light confinement in a plasmonic nanocavity." *Scientific reports* 7, no. 1 (2017): 1-8.

[51] N. T. Yardimci, and M. Jarrahi. "High sensitivity terahertz detection through large-area plasmonic nano-antenna arrays." *Scientific reports* 7, no. 1 (2017): 1-8.

[52] N. T. Yardimci, and M. Jarrahi. "Nanostructure-Enhanced Photoconductive Terahertz Emission and Detection." *Small* 14, no. 44 (2018): 1802437.

[53] C. W. Berry, M. R. Hashemi, S. Preu, H. Lu, A. C. Gossard, and M. Jarrahi. "High power terahertz generation using 1550 nm plasmonic photomixers." *Applied Physics Letters* 105, no. 1 (2014): 011121.

[54] N. Wang, S. Cakmakyapan, Y-J. Lin, H. Javadi, and M. Jarrahi. "Room-temperature heterodyne terahertz detection with quantum-level sensitivity." *Nature Astronomy* 3, no. 11 (2019): 977-982.

[55] T. Kampfrath, M. Battiato, P. Maldonado, et al. "Terahertz spin current pulses controlled by magnetic heterostructures." *Nature nanotechnology* 8, no. 4 (2013): 256-260.

[56] T. Seifert, S. Jaiswal, U. Martens, et al. "Efficient metallic spintronic emitters of ultrabroadband terahertz radiation." *Nature photonics* 10, no. 7 (2016): 483-488.

[57] L. Prechtel, L. Song, D. Schuh, P. Ajayan, W. Wegscheider, and A. W. Holleitner. "Time-resolved ultrafast photocurrents and terahertz generation in freely suspended graphene." *Nature communications* 3, no. 1 (2012): 1-7.

[58] M. Mittendorff, S. Winnerl, and T. E. Murphy. "2D THz Optoelectronics." *Advanced Optical Materials* 9, no. 3 (2021): 2001500.

[59] K. Peng, P. Parkinson, L. Fu, et al. "Single nanowire photoconductive terahertz detectors." *Nano letters* 15, no. 1 (2015): 206-210.

[60] S. Preu, G. H. Döhler, S. Malzer, L. Wang, and A. C. Gossard, "Tunable, continuous-wave terahertz photomixer sources and applications." *Journal of Applied Physics* 109, no. 6 (2011): 4.

[61] S. Preu. "A unified derivation of the terahertz spectra generated by photoconductors and diodes." *Journal of Infrared, Millimeter, and Terahertz Waves*, 35, (2014): 998-1010.

~~[61]~~[62] G. Carpintero, E. Garcia-Munoz, H. Hartnagel, S. Preu, and A. Raisanen, (2015). *Semiconductor terahertz technology: devices and systems at room temperature operation*. John Wiley & Sons., chapter 2
~~[62]~~[63] http://www.ioffe.ru/SVA/NSM/Semicond/ accessed Nov. 30th, 2021
~~[63]~~[64] C. Müller-Landau, S. Malzer, H.B. Weber, et al. "Terahertz generation with ballistic photodiodes under pulsed operation." *Semiconductor Science and Technology* 33, no. 11 (2018): 114015.
~~[64]~~[65] H. Roehle, R. J. B. Dietz, H. J. Hensel, et al. "Next generation 1.5 µm terahertz antennas: mesa-structuring of InGaAs/InAlAs photoconductive layers." *Optics express* 18, no. 3 (2010): 2296-2301.
~~[65]~~[66] E.R. Brown. "THz Generation by Photomixing in Ultrafast Photoconductors." *Int. J. High Speed Electron. and Systems* 13, 497-545 (2003)
~~[66]~~[67] S. Verghese, K.A. McIntosh, and E.R. Brown. "Optical and terahertz power limits in the low-temperature-grown GaAs photomixers." *Applied Physics Letters*, 71, no. 19 (1997): 2743-2745.
[68] A.D.J. Fernandez Olvera, A. Roggenbuck, K. Dutzi, et al. "International System of Units (SI) traceable noise equivalent power and responsivity characterization of continuous wave terahertz detectors." *Photonics* 13, no. 1 (2019): 15.
~~[67]~~[69] G. Ducournau, Y. Yoshimizu, S. Hisatake, et al. "Coherent THz communication at 200 GHz using a frequency comb, UTC-PD and electronic detection." *Electronics letters* 50, no. 5 (2014): 386-388.
~~[68]~~[70] T. Ishibashi, Y. Muramoto, T. Yoshimatsu and H. Ito. "Unitraveling-Carrier Photodiodes for Terahertz Applications." *IEEE Journal of Selected Topics in Quantum Electronics* 20, no. 6 (2014): 79-88.
~~[69]~~[71] Q. Li, K. Li, Y. Fu, et al. "High-Power Flip-Chip Bonded Photodiode With 110 GHz Bandwidth." *Journal of Lightwave Technology* 34, no. 9 (2016): 2139-2144.
~~[70]~~[72] http://www.ioffe.ru/SVA/NSM/Semicond/InP/bandstr.html accessed Nov. 30th, 2021
~~[71]~~[73] A. Beck, G. Ducournau, M. Zaknoune, et al. "High-efficiency uni-travelling-carrier photomixer at 1.55 mm and spectroscopy application up to 1.4 THz." *Electronics Letters* 44, no. 22 (2008): 1320-1322.
~~[72]~~[74] E. Rouvalis, C. C. Renaud, D. G. Moodie, M. J. Robertson and A. J. Seeds. "Continuous Wave Terahertz Generation from Ultra-Fast InP-Based Photodiodes." *IEEE Transactions on microwave theory and techniques* 60, no. 3 (2012): 509-517.
~~[73]~~[75] C. C. Renaud, M. Natrella, C. Graham, J. Seddon, F. Van Dijk and A. J. Seeds. "Antenna Integrated THz Uni-Traveling Carrier Photodiodes." *IEEE Journal of Selected Topics in Quantum Electronics* 24, no. 2 (2017): 1-11.
~~[74]~~[76] H. Ito, Y. Muramoto, T. Furuta, and Y. Hirota. "High-speed and high-output-power uni-traveling-carrier photodiodes." In *2005 IEEE LEOS Annual Meeting Conference Proceedings*, pp. 456-457. IEEE, 2005.
~~[75]~~[77] G. Zhou, P. Runge, S. Keyvaninia, et al. "High-Power InP-Based Waveguide Integrated Modified Uni-Traveling-Carrier Photodiodes." *Journal of Lightwave Technology* 35, no. 4 (2017): 717-721.
~~[76]~~[78] S. Nellen, T. Ishibashi, A. Deninger, et al. "Experimental Comparison of UTC- and PIN-Photodiodes for Continuous-Wave Terahertz Generation." *Journal of Infrared, Millimeter, and Terahertz Waves* (2019): 1-12.
~~[77]~~[79] O. I. Dosunmu, D. D. Cannon, M. K. Emsley, et al. "Resonant cavity enhanced Ge photodetectors for 1550 nm operation on reflecting Si substrates." *IEEE Journal of selected topics in quantum electronics* 10, no. 4 (2004): 694-701.
~~[78]~~[80] V. Rymanov, A. Stöhr, S. Dülme, and T. Tekin, "Triple transit region photodiodes (TTR-PDs) providing high millimeter wave output power." *Optics Express* 22, no. 7 (2014): 7550-7558.
~~[79]~~[81] S. Nellen, R. Kohlhaas, L. Liebermeister, S. Breuer, B. Globisch, and M. Schell. "Continuous wave terahertz generation from photodiode-based emitters with up to 200 µW terahertz power." In *2018 43rd International Conference on Infrared, Millimeter, and Terahertz Waves (IRMMW-THz)*, pp. 1-2. IEEE, 2018.
~~[80]~~[82] P. Latzel, F. Pavanello, M. Billet, et al. "Generation of mW level in the 300-GHz band using resonant-cavity-enhanced unitraveling carrier photodiodes." *IEEE Transactions on Terahertz Science and Technology* 7, no. 6 (2017): 800-807.
~~[81]~~[83] M. M. Aller, P. Lu, V. Rymanov, A. Stöhr, and S. Preu. "Side-illuminated fully ballistic pin diode-based photomixer at 1550 nm." In *2020 45th International Conference on Infrared, Millimeter, and Terahertz Waves (IRMMW-THz)*, pp. 1-2. IEEE, 2020.
~~[82]~~[84] S. Preu, F. H. Renner, S. Malzer, et al. "Efficient Terahertz emission from ballistic transport enhanced n-i-p-n-i-p superlattice photomixers." *Applied physics letters* 90, no. 21 (2007): 212115.
~~[83]~~[85] J. P. Seddon, M. Natrella, X. Lin, C. Graham, C. C. Renaud and A. J. Seeds. "Photodiodes for Terahertz Applications." *IEEE Journal of Selected Topics in Quantum Electronics* 28, no. 2 (2021): 1-12.
~~[84]~~[86] P. Offermans, L. Zhang, P. De Heyn, et al. "Continuous wave generation up to 1.3 THz using antenna-coupled silicon-integrated Ge photodiodes." In *2018 11th UK-Europe-China Workshop on Millimeter Waves and Terahertz Technologies (UCMMT)*, vol. 1, pp. 1-2. IEEE, 2018.
~~[85]~~[87] E. Lacombe, F. Gianesello, C. Durand, G. Ducournau, C. Luxey and D. Gloria, "Sub-THz source integrated in industrial silicon Photonic technology targeting high data rate wireless applications." In *2017 IEEE 17th Topical Meeting on Silicon Monolithic Integrated Circuits in RF Systems (SiRF)*, pp. 26-29. IEEE, 2017.
~~[86]~~[88] M. Tani, S. Matsuura, K. Sakai, and S. I. Nakashima. "Emission characteristics of photoconductive antennas based on low-temperature-grown GaAs and semi-insulating GaAs." *Applied optics* 36, no. 30 (1997): 7853-7859.
~~[87]~~[89] M. Beck, Hanjo Schäfer, Gregor Klatt, et al. "Impulsive terahertz radiation with high electric fields from an amplifier-driven large-area photoconductive antenna." *Optics Express* 18, no. 9 (2010): 9251-9257.
~~[88]~~[90] M. Venkatesh, K. S. Rao, T. S. Abhilash, S. P. Tewari, and A. K. Chaudhary. "Optical characterization of GaAs photoconductive antennas for efficient generation and detection of Terahertz radiation." *Optical Materials* 36, no. 3 (2014): 596-601.
~~[89]~~[91] P. J. Hale, J. Madeo, C. Chin, et al. "20 THz broadband generation using semi-insulating GaAs interdigitated photoconductive antennas." *Optics express* 22, no. 21 (2014): 26358-26364.
~~[90]~~[92] A. Abbes, P-K. Lu, P. Nouvel, et al. "280 GHz Radiation Source Driven by a 1064nm Continuous-Wave Dual-Frequency Vertical External Cavity Semiconductor Laser." In *2021 46th International Conference on Infrared, Millimeter and Terahertz Waves (IRMMW-THz)*, pp. 1-2. IEEE, 2021.

~~[91]~~[93] P-K. Lu, and Mona Jarrahi. "A Continuous-wave Terahertz Self-heterodyne Spectroscopy System without Using Short-carrier-lifetime Photoconductors." In *2021 46th International Conference on Infrared, Millimeter and Terahertz Waves (IRMMW-THz)*, pp. 1-2. IEEE, 2021.
~~[92]~~[94] W. Wang, P-K. Lu, A. K. Vinod, et al. "High spectral purity chip-scale tunable THz radiation source." In *CLEO: Applications and Technology*, pp. ATu2T-3. Optical Society of America, 2021.
~~[93]~~[95] D. Turan, N. T. Yardimci, and M. Jarrahi. "Plasmonics-enhanced photoconductive terahertz detector pumped by Ytterbium-doped fiber laser." *Optics express* 28, no. 3 (2020): 3835-3845.
~~[94]~~[96] P-K. Lu, D. Turan, and M. Jarrahi. "High-sensitivity telecommunication-compatible photoconductive terahertz detection through carrier transit time reduction." *Optics Express* 28, no. 18 (2020): 26324-26335.
~~[95]~~[97] D. Turan, P-K. Lu, N. T. Yardimci, et al. "Wavelength conversion through plasmon-coupled surface states." *Nature communications* 12, no. 1 (2021): 1-8.
~~[96]~~[98] S. G. Park, Y. Choi, Y-J. Oh, and K-H. Jeong. "Terahertz photoconductive antenna with metal nanoislands." *Optics express* 20, no. 23 (2012): 25530-25535.
~~[97]~~[99] A. Jooshesh, L. Smith, M. Masnadi-Shirazi, et al. "Nanoplasmonics enhanced terahertz sources." *Optics express* 22, no. 23 (2014): 27992-28001.
~~[98]~~[100] N. T. Yardimci, H. Lu, and M. Jarrahi. "High power telecommunication-compatible photoconductive terahertz emitters based on plasmonic nano-antenna arrays." *Applied physics letters* 109, no. 19 (2016): 191103.
~~[99]~~[101] D. Turan, S. C. ~~W. Berry, M. R. Hashemi~~Corzo-Garcia, N. T. Yardimci, E. Castro-Camus, and M. Jarrahi. ~~"Generation~~"Impact of ~~high power pulsed terahertz~~ the metal adhesion layer on the radiation ~~using a~~power of plasmonic photoconductive ~~emitter array with logarithmic spiral antennas." *Applied Physics Letters* 104~~terahertz sources." *Journal of Infrared, Millimeter, and Terahertz Waves* 38, no. ~~8 (2014): 081122.~~ 12 (2017): 1448-1456.
~~[100]~~[102] S. H. Yang, M. R. Hashemi, C. W. Berry, and M. Jarrahi. "7.5% optical-to-terahertz conversion efficiency offered by photoconductive emitters with three-dimensional plasmonic contact electrodes." *IEEE Transactions on Terahertz Science and Technology* 4, no. 5 (2014): 575-581.
~~[101]~~[103] N. T. Yardimci, S. H. Yang, C. W. Berry, and M. Jarrahi. "High-power terahertz generation using large-area plasmonic photoconductive emitters." *IEEE Transactions on Terahertz Science and Technology* 5, no. 2 (2015): 223-229.
~~[102]~~[104] J. A. Schuller, E. S. Barnard, W. Cai, Y. C. Jun, J. S. White, and M. L. Brongersma. "Plasmonics for extreme light concentration and manipulation." *Nature materials* 9, no. 3 (2010): 193-204.
~~[103]~~[105] A. Hartschuh, E. J. Sánchez, X. S. Xie, and L. Novotny. "High-resolution near-field Raman microscopy of single-walled carbon nanotubes." *Physical Review Letters* 90, no. 9 (2003): 095503.
~~[104]~~[106] H. G. Frey, S. Witt, K. Felderer, and R. Guckenberger. "High-resolution imaging of single fluorescent molecules with the optical near-field of a metal tip." *Physical review letters* 93, no. 20 (2004): 200801.
~~[105]~~[107] T. Ishi, J. Fujikata, K. Makita, T. Baba, and K. Ohashi. "Si nano-photodiode with a surface plasmon antenna." *Japanese Journal of Applied Physics* 44, no. 3L (2005): L364
~~[106]~~[108] L. Tang, S. E. Kocabas, S. Latif, et al. "Nanometre-scale germanium photodetector enhanced by a near-infrared dipole antenna." *Nature Photonics* 2, no. 4 (2008): 226-229.
~~[107]~~[109] M. R. Hashemi, S. H. Yang, T. Wang, N. Sepúlveda, and M. Jarrahi. "Electronically-controlled beam-steering through vanadium dioxide metasurfaces." *Scientific reports* 6, no. 1 (2016): 1-8.
~~[108]~~[110] W. Mönch. "On the physics of metal-semiconductor interfaces." *Reports on Progress in Physics* 53, no. 3 (1990): 221.
~~[109]~~[111] W. Mönch. "Barrier heights of real Schottky contacts explained by metal-induced gap states and lateral inhomogeneities." *Journal of Vacuum Science & Technology B: Microelectronics and Nanometer Structures Processing, Measurement, and Phenomena* 17, no. 4 (1999): 1867-1876
~~[110]~~[112] J. Tersoff. "Schottky barriers and semiconductor band structures." *Physical Review B* 32, no. 10 (1985): 6968.
~~[111]~~[113] L. F. J. Piper, T. D. Veal, M. J. Lowe, and C. F. McConville. "Electron depletion at InAs free surfaces: Doping-induced acceptorlike gap states." *Physical Review B* 73, no. 19 (2006): 195321.
~~[112]~~[114] K. Liu, J. Xu, T. Yuan, and X. C. Zhang. "Terahertz radiation from InAs induced by carrier diffusion and drift." *Physical Review B* 73, no. 15 (2006): 155330.
~~[113]~~[115] P-K. Lu, D. Turan, and M. Jarrahi~~,~~. "High-Power Terahertz Pulse Generation from Bias-Free Nanoantennas on Graded Composition InGaAs Structures." ~~In Review~~*Optics Express* 30, no. 2 (2022): 1584-1598.
~~[114]~~[116] A. Hirohata, K. Yamada, Y. Nakatani, et al. "Review on spintronics: Principles and device applications." *Journal of Magnetism and Magnetic Materials* 509 (2020): 166711.
~~[115]~~[117] O. Gueckstock, L. Nádvorník, T. S. Seifert, et al. "Modulating the polarization of broadband terahertz pulses from a spintronic emitter at rates up to 10 kHz." *Optica* 8, no. 7 (2021): 1013-1019.
~~[116]~~[118] S. M. Rouzegar, L. Brandt, L. Nadvornik, et al. "Laser-induced terahertz spin transport in magnetic nanostructures arises from the same force as ultrafast demagnetization." arXiv, 2021: p. 2103.11710.
~~[117]~~[119] J. Sinova, S.O. Valenzuela, J. Wunderlich, et al. "Spin Hall effects." *Reviews of modern physics* 87, no. 4 (2015): 1213.
~~[118]~~[120] G. E. Bauer, E. Saitoh, and B. J. van Wees. "Spin caloritronics." *Nature materials* 11, no. 5 (2012): 391-399.
~~[119]~~[121] Y. K. Kato, R. C. Myers, A. C. Gossard, and D. D. Awschalom. "Observation of the spin Hall effect in semiconductors." *science* 306, no. 5703 (2004): 1910-1913.
~~[120]~~[122] R. I. Herapath, S. M. Hornett, T. S. Seifert, et al. "Impact of pump wavelength on terahertz emission of a cavity-enhanced spintronic trilayer." *Applied Physics Letters* 114, no. 4 (2019): 041107.
~~[121]~~[123] E. T. Papaioannou, G. Torosyan, S. Keller, et al. "Efficient Terahertz Generation Using Fe/Pt Spintronic Emitters Pumped at Different Wavelengths." *IEEE Transactions on Magnetics* 54, no. 11 (2018): 1-5.
~~[122]~~[124] M. T. Hibberd, D. S. Lake, N. A. B. Johansson, et al. "Magnetic-field tailoring of the terahertz polarization emitted from a spintronic source." *Applied Physics Letters* 114, no. 3 (2019): 031101.

~~[123]~~[125] Y. Wu, M. Elyasi, X. Qiu, et al. “High-Performance THz Emitters Based on Ferromagnetic/Nonmagnetic Heterostructures.” *Advanced Materials* 29, no. 4 (2017): 1603031.
~~[124]~~[126] T. S. Seifert, S. Jaiswal, M. Sajadi, et al. “Ultrabroadband single-cycle terahertz pulses with peak fields of 300 kV cm^{-1} from a metallic spintronic emitter.” *Applied Physics Letters* 110, no. 25 (2017): 252402.
~~[125]~~[127] D. Yang, J. Liang, C. Zhou, et al. “Powerful and Tunable THz Emitters Based on the Fe/Pt Magnetic Heterostructure.” *Advanced Optical Materials* 4, no. 12 (2016): 1944-1949.
~~[126]~~[128] S.-C. Chen, Z. Feng, J. Li, et al. “Ghost spintronic THz-emitter-array microscope.” *Light: Science & Applications* 9, no. 1 (2020): 1-9.
~~[127]~~[129] C. Zhou, Y. P. Liu, Z. Wang, et al. “Broadband Terahertz Generation via the Interface Inverse Rashba-Edelstein Effect.” *Physical review letters* 121, no. 8 (2018): 086801.
~~[128]~~[130] T. S. Seifert, N. M. Tran, O. Gueckstock, et al. “Terahertz spectroscopy for all-optical spintronic characterization of the spin-Hall-effect metals Pt, W and $Cu_{80}Ir_{20}$.” *Journal of Physics D: Applied Physics* 51, no. 36 (2018): 364003.
~~[129]~~[131] T. S. Seifert, U. Martens, F. Radu, et al. “Frequency-Independent Terahertz Anomalous Hall Effect in $DyCo_5$, $Co_{32}Fe_{68}$, and $Gd_{27}Fe_{73}$ Thin Films from DC to 40 THz.” *Advanced Materials* 33, no. 14 (2021): 2007398.
~~[130]~~[132] F. Bonell, M. Goto, G. Sauthier, et al. "Control of Spin–Orbit Torques by Interface Engineering in Topological Insulator Heterostructures." *Nano Letters* 20, no. 8 (2020): 5893-5899.
~~[131]~~[133] L. Cheng, X. Wang, W. Yang, et al. "Far out-of-equilibrium spin populations trigger giant spin injection into atomically thin MoS_2." *Nature Physics* 15, no. 4 (2019): 347-351.
~~[132]~~[134] X. Zhou, B. Song, X. Chen, et al. “Orientation-dependent THz emission in non-collinear antiferromagnetic Mn3Sn and Mn3Sn-based heterostructures.” *Applied Physics Letters* 115, no. 18 (2019): 182402.
~~[133]~~[135] P. Stremoukhov, A. Safin, M. Logunov, S. Nikitov, and A. Kirilyuk. “Spintronic terahertz-frequency nonlinear emitter based on the canted antiferromagnet-platinum bilayers.” *Journal of Applied Physics* 125, no. 22 (2019): 223903.
~~[134]~~[136] M. B. Jungfleisch, Q. Zhang, W. Zhang, et al. “Control of terahertz emission by ultrafast spin-charge current conversion at Rashba interfaces." *Physical review letters* 120, no. 20 (2018): 207207.
~~[135]~~[137] T. S. Seifert, U. Martens, S. Günther, et al. “Terahertz Spin Currents and Inverse Spin Hall Effect in Thin-Film Heterostructures Containing Complex Magnetic Compounds.” In *Spin*, vol. 7, no. 03, p. 1740010. World Scientific Publishing Company, 2017.
~~[136]~~[138] T. S. Seifert, S. Jaiswal, J. Barker, et al. “Femtosecond formation dynamics of the spin Seebeck effect revealed by terahertz spectroscopy.” *Nature communications* 9, no. 1 (2018): 1-11.
~~[137]~~[139] P. Jiménez-Cavero, O. Gueckstock, L. Nádvorník, et al. “Tuning laser-induced terahertz spin currents from torque-to conduction-electron-mediated transport.” *arXiv preprint arXiv:2110.05462* (2021).
~~[138]~~[140] H.S. Qiu, K. Kato, K. Hirota, et al. “Layer thickness dependence of the terahertz emission based on spin current in ferromagnetic heterostructures.” *Optics express* 26, no. 12 (2018): 15247-15254.
~~[139]~~[141] J. P. Ferrolino, N. I. Cabello, A. De Los Reyes, et al. “Thickness dependence of the spintronic terahertz emission from Ni/Pt bilayer grown on MgO via electron beam deposition.” *Applied Physics Express* 14, no. 9 (2021): 093001.
~~[140]~~[142] U. Nandi, M. S. Abdelaziz, S. Jaiswal, et al. “Antenna-coupled spintronic terahertz emitters driven by a 1550 nm femtosecond laser oscillator.” *Applied Physics Letters* 115, no. 2 (2019): 022405.
~~[141]~~[143] M. Talara, D. S. Bulgarevich, C. Tachioka, et al. “Efficient terahertz wave generation of diabolo-shaped Fe/Pt spintronic antennas driven by a 780 nm pump beam.” *Applied Physics Express* 14, no. 4 (2021): 042008.
~~[142]~~[144] C. L. Davies, J. Borchert, C. Q. Xia, et al. “Impact of the Organic Cation on the Optoelectronic Properties of Formamidinium Lead Triiodide.” *The journal of physical chemistry letters* 9, no. 16 (2018): 4502-4511.
~~[143]~~[145] R. L. Milot, M. T. Klug, C. L. Davies, et al. “The Effects of Doping Density and Temperature on the Optoelectronic Properties of Formamidinium Tin Triiodide Thin Films.” *Advanced Materials* 30, no. 44 (2018): 1804506.
~~[144]~~[146] C. L. Davies, J. B. Patel, C. Q. Xia, et al. “Temperature-Dependent Refractive Index of Quartz at Terahertz Frequencies.” *Journal of Infrared, Millimeter, and Terahertz Waves* 39, no. 12 (2018): 1236-1248.
~~[145]~~[147] V. Balos, P. Müller, G. Jakob, M. Kläui, and M. Sajadi. “Imprinting the complex dielectric permittivity of liquids into the spintronic terahertz emission.” *Applied Physics Letters* 119, no. 9 (2021): 091104.
~~[146]~~[148] L. Nádvorník, M. Borchert, L. Brandt, et al. “Broadband Terahertz Probes of Anisotropic Magnetoresistance Disentangle Extrinsic and Intrinsic Contributions.” *Physical Review X* 11, no. 2 (2021): 021030.
~~[147]~~[149] H. Zhao, X. Chen, C. Ouyang, et al. “Generation and manipulation of chiral terahertz waves in the three-dimensional topological insulator Bi2Te3.” *Advanced Photonics* 2, no. 6 (2020): 066003.
~~[148]~~[150] L. Braun, G. Mussler, A. Hruban, et al. “Ultrafast photocurrents at the surface of the three-dimensional topological insulator Bi2Se3.” *Nature communications* 7, no. 1 (2016): 1-9.
~~[149]~~[151] H. Qiu, L. Wang, Z. Shen, et al. “Magnetically and electrically polarization-tunable THz emitter with integrated ferromagnetic heterostructure and large-birefringence liquid crystal.” *Applied Physics Express* 11, no. 9 (2018): 092101.
~~[150]~~[152] Z. Bai, Y. Liu, R. Kong, et al. “Near-field Terahertz Sensing of HeLa Cells and Pseudomonas Based on Monolithic Integrated Metamaterials with a Spintronic Terahertz Emitter.” *ACS Applied Materials & Interfaces* 12, no. 32 (2020): 35895-35902.
~~[151]~~[153] D. S. Bulgarevich, Y. Akamine, M. Talara, et al. “Terahertz Magneto-Optic Sensor/Imager.” *Scientific reports* 10, no. 1 (2020): 1-7.
~~[152]~~[154] M. Muller, N. M. Sabanés, T. Kampfrath, and M. Wolf. “Phase-Resolved Detection of Ultrabroadband THz Pulses inside a Scanning Tunneling Microscope Junction.” *ACS photonics* 7, no. 8 (2020): 2046.
~~[153]~~[155] J. M. Kikkawa, and D. D. Awschalom. “Lateral drag of spin coherence in gallium arsenide.” *Nature* 397, no. 6715 (1999): 139-141.
~~[154]~~[156] J. Lee, K. F. Mak, and J. Shan. “Electrical control of the valley Hall effect in bilayer MoS_2 transistors.” *Nature nanotechnology* 11, no. 5 (2016): 421-425.
~~[155]~~[157] T. Kampfrath, A. Sell, G. Klatt, et al. “Coherent terahertz control of antiferromagnetic spin waves.” *Nature Photonics* 5, no. 1 (2011): 31-34.
~~[156]~~[158] O. Gomonay, T. Jungwirth, and J. Sinova. “Narrow-band tunable terahertz detector in antiferromagnets via staggered-field and antidamping torques.” *Physical Review B* 98, no. 10 (2018): 104430.

~~[157]~~[159] G. Ramakrishnan, N. Kumar, G. K. Ramanandan, A. J. Adam, R. W. Hendrikx, and P. C. Planken. “Plasmon-enhanced terahertz emission from a semiconductor/metal interface.” *Applied Physics Letters* 104, no. 7 (2014): 071104.

~~[158]~~[160] O. Gueckstock, L. Nadvornik, M. Gradhand, et al. “Terahertz Spin-to-Charge Conversion by Interfacial Skew Scattering in Metallic Bilayers.” *Advanced Materials* 33, no. 9 (2021): 2006281.

~~[159]~~[161] W. Hoppe, J. Weber, S. Tirpanci, O. Gueckstock, T. Kampfrath, and G. Woltersdorf. “On-Chip Generation of Ultrafast Current Pulses by Nanolayered Spintronic Terahertz Emitters.” *ACS Applied Nano Materials* 4, no. 7 (2021): 7454-7460.

~~[160]~~[162] D. De Fazio, D. G. Purdie, A. K. Ott, et al. “High-Mobility, wettransferred graphene grown by chemical vapor deposition.” *ACS Nano* 13, no. 8 (2019): 8926-8935.

~~[161]~~[163] M. Breusing, C. Ropers, and T. Elsaesser. "Ultrafast carrier dynamics in graphite." *Physical review letters* 102, no. 8 (2009): 086809.

~~[162]~~[164] G. Ramakrishnan, R. Chakkittakandy, and P. C. Planken. "Terahertz generation from graphite." *Optics express* 17, no. 18 (2009): 16092-16099.

~~[163]~~[165] Y-M. Bahk, G. Ramakrishnan, J. Choi, et al. "Plasmon enhanced terahertz emission from single layer graphene." *ACS nano* 8, no. 9 (2014): 9089-9096.

~~[164]~~[166] P. A. Obraztsov, P. A. Chizhov, T. Kaplas, et al. "Coherent detection of terahertz radiation with graphene." *ACS Photonics* 6, no. 7 (2019): 1780-1788.

~~[165]~~[167] N. Hunter, A. S. Mayorov, C. D. Wood, et al. "On-chip picosecond pulse detection and generation using graphene photoconductive switches." *Nano letters* 15, no. 3 (2015): 1591-1596.

~~[166]~~[168] A. J. Jumaah, S. Al-Daffaie, O. Yilmazoglu, and T. Kusserow. "Continuous-wave terahertz emitter with hybrid nanoelectrodes based on graphene and nanowire." *OSA continuum* 3, no. 7 (2020): 1826-1833.

~~[167]~~[169] L. Zhu, Y. Huang, C. He, et al. "Transient Anisotropic Photocurrent Induced Terahertz Emission from the Surface of Black Phosphorus." *physica status solidi (RRL)–Rapid Research Letters* 15, no. 11 (2021): 2100413.

~~[168]~~[170] M. H. Doha, J. I. Santos Batista, A. F. Rawwagah, et al. "Integration of multi-layer black phosphorus into photoconductive antennas for THz emission." *Journal of Applied Physics* 128, no. 6 (2020): 063104.

~~[169]~~[171] M. Mittendorff, R. J. Suess, E. Leong, and T. E. Murphy. "Optical gating of black phosphorus for terahertz detection." *Nano letters* 17, no. 9 (2017): 5811-5816.

~~[170]~~[172] J. Wu, B. M. Borg, D. Jacobsson, K. A. Dick, and L. E. Wernersson. "Control of composition and morphology in InGaAs nanowires grown by metalorganic vapor phase epitaxy." *Journal of crystal growth* 383 (2013): 158-165.

~~[171]~~[173] H. J. Joyce, C. J. Docherty, Q. Gao, et al. "Electronic properties of GaAs, InAs and InP nanowires studied by terahertz spectroscopy." *Nanotechnology* 24, no. 21 (2013): 214006.

~~[172]~~[174] J. L. Boland, G. Tütüncüoglu, J. Q. Gong, et al. "Towards higher electron mobility in modulation doped GaAs/AlGaAs core shell nanowires." *Nanoscale* 9, no. 23 (2017): 7839-7846.

[175] L. Balaghi, S. Shan, I. Fotev, et al. "High electron mobility in strained GaAs nanowires." *Nature communications* 12, no. 1 (2021): 1-11.

~~[173]~~[176] K. Peng, P. Parkinson, J. L. Boland, et al. "Broadband phase-sensitive single InP nanowire photoconductive terahertz detectors." *Nano Letters* 16, no. 8 (2016): 4925-4931.

~~[174]~~[177] K. Peng, P. Parkinson, Q. Gao et al. "Single n+-i-n+ InP nanowires for highly sensitive terahertz detection." *Nanotechnology* 28, no. 12 (2017): 125202.

~~[175]~~[178] K. Peng, D. Jevtics, F. Zhang, et al. "Three-dimensional cross-nanowire networks recover full terahertz state." *Science* 368, no. 6490 (2020): 510-513.
